# Site selective C–H functionalization of *Mitragyna* alkaloids reveals a molecular switch for tuning opioid receptor signaling efficacy

**DOI:** 10.1038/s41467-021-23736-2

**Published:** 2021-06-22

**Authors:** Srijita Bhowmik, Juraj Galeta, Václav Havel, Melissa Nelson, Abdelfattah Faouzi, Benjamin Bechand, Mike Ansonoff, Tomas Fiala, Amanda Hunkele, Andrew C. Kruegel, John. E. Pintar, Susruta Majumdar, Jonathan A. Javitch, Dalibor Sames

**Affiliations:** 1 Department of Chemistry, Columbia University, New York, NY USA; 2 Institute of Organic Chemistry and Biochemistry of the Czech Academy of Sciences (IOCB Prague), 160 00 Prague 6, Czech Republic; 3 Department of Psychiatry, and Department of Molecular Pharmacology and Therapeutics, Columbia University, New York, NY USA; 4 Division of Molecular Therapeutics, New York State Psychiatric Institute, New York, NY USA; 5 Center for Clinical Pharmacology, St Louis College of Pharmacy and Washington University School of Medicine, St Louis, MO 63110 USA; 6 University of California San Diego, La Jolla, CA 92161 USA; 7 Department of Neuroscience and Cell Biology, Rutgers University, New Jersey, NJ 08854 USA; 8 Laboratory of Organic Chemistry, ETH Zürich, 8093 Zürich, Switzerland; 9 Department of Neurology and Molecular Pharmacology, Memorial Sloan Kettering Cancer Center, New York, NY 10021 USA; 10 NeuroTechnology Center at Columbia University, New York, NY USA; 11 The Zuckerman Mind Brain Behavior Institute at Columbia University, New York, NY USA

**Keywords:** Natural products, Receptor pharmacology, Synthetic chemistry methodology

## Abstract

Mitragynine (MG) is the most abundant alkaloid component of the psychoactive plant material “kratom”, which according to numerous anecdotal reports shows efficacy in self-medication for pain syndromes, depression, anxiety, and substance use disorders. We have developed a synthetic method for selective functionalization of the unexplored C11 position of the MG scaffold (C6 position in indole numbering) via the use of an indole-ethylene glycol adduct and subsequent iridium-catalyzed borylation. Through this work we discover that C11 represents a key locant for fine-tuning opioid receptor signaling efficacy. 7-Hydroxymitragynine (7OH), the parent compound with low efficacy on par with buprenorphine, is transformed to an even lower efficacy agonist by introducing a fluorine substituent in this position (11-F-7OH), as demonstrated in vitro at both mouse and human mu opioid receptors (mMOR/hMOR) and in vivo in mouse analgesia tests. Low efficacy opioid agonists are of high interest as candidates for generating safer opioid medications with mitigated adverse effects.

## Abstract (web summary)

Mitragynine (MG) is an indole alkaloid from kratom plant that binds opioid receptors and as such presents a scaffold for the development of atypical opioid receptor modulators. Here, the authors report a synthetic method for selective functionalization of the C11 position of MG, and show that this position is essential for fine-tuning opioid receptor signaling efficacy.

## Introduction

**Fig. 1:**
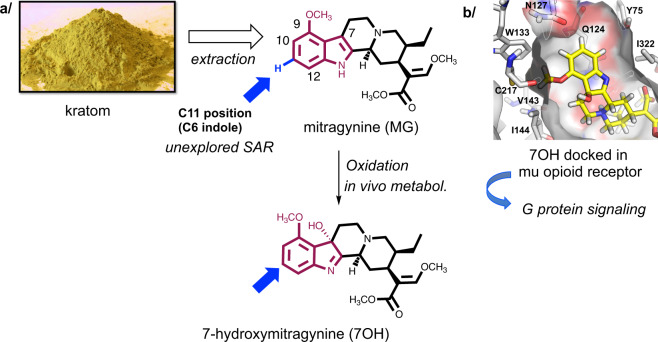
The rationale for selective C–H bond functionalization of mitragynine (MG) and 7-hydroxymitragynine (7OH). **a** MG is readily obtained in multigram quantities from kratom powder by extraction, hence there is an incentive to develop selective functionalization of MG and related scaffolds via late-stage C–H bond functionalization. Specifically, the C11 position has not been explored in terms of mu-opioid receptor (MOR) signaling and other biological effects, due to the lack of chemistries for functionalization of this position. **b** The top-right panel shows a docking pose of 7OH in human MOR highlighting the aromatic ring and the proposed binding pocket in the receptor.

In search of molecules with robust clinical effects in the area of central nervous system (CNS) disorders and ability to repair synaptic function in the brain, we have been led to atypical modulators of endogenous opioid signaling^1–4^. In this context, we became interested in the psychoactive plant *Mitragyna speciosa* that has been used for centuries in Southeast Asia for treatment of pain, fatigue, opium dependence, and a number of other ailments. In the US, the use of the dry leaf material, known as “kratom”, has been on the rise in the last decade, along with the number of anecdotal reports that point to the efficacy of kratom in a range of disorders with limited therapeutic options, including opioid dependence, treatment-resistant depression, anxiety, and pain syndromes^5–12^. A number of alkaloids in this plant, including mitragynine (MG) and its oxidation product 7-hydroxymitragynine (7OH, Fig. 1), have been found to bind to opioid receptors and represent molecular scaffolds for the development of opioid receptor modulators^13,14^. MG, as an indole alkaloid, shows no structural resemblance to traditional morphine-type compounds and represents an atypical opioid ligand with distinct signaling properties and physiological effects compared to clinically used opioid analgesics. For example, we have shown that MG is a partial MOR agonist (mu-opioid receptor agonist) with a potential bias for G protein signaling (showing no *β*-arrestin-2 recruitment) in cell-based assays^15,16^. Further, we have found that 7OH is a more potent and efficacious partial MOR agonist compared to MG, and that it acts as a potent analgesic in mice^15,16^. The relative activities of MG and 7OH are highly relevant, as we recently reported that 7OH is formed in vivo from MG and mediates MG’s analgesic effects in mice^17^. In additional preclinical studies, others have found that MG exhibits antinociceptive effects in dogs comparable to those of codeine but with less respiratory depression^18^, and that the compound is not self-administered by rats, but instead, inhibits self-administration of morphine and heroin^19,20^.

The study and development of safer opioids is a long-standing scientific and societal goal, and a part of the scientific strategy proposed by the National Institute of Health (NIH) initiative to address the opioid crisis^21,22^. Further, there is a growing interest in the use of opioid receptor modulators as medicaments for depression and anxiety disorders (and other psychiatric diseases)^1,23^. Therefore, safer opioid modulators represent promising treatments for disorders spanning a wide spectrum of physical and emotional pain. Accordingly, further understanding of kratom and its alkaloids has major implications for public health.

Our previous report provided a basic structure–activity relationship (SAR) map of MG, in terms of in vitro opioid receptor pharmacology, with respect to the substituents on the saturated rings of MG. This work was enabled by the total enantioselective synthesis of MG developed in our laboratories^15^. Several other research teams have also reported total syntheses of MG and related compounds^24–28^. These de novo synthetic approaches offer nearly unlimited exploration of complex MG-related molecules, but they are labor intensive due to the high number of synthetic steps. Thus, the need to access many different derivatives at multiple positions demands more efficient synthetic strategies. MG can be extracted from kratom leaf matter in multigram quantities (~1% of dry kratom mass^14,15^), and therefore, there is a strong incentive to develop synthetic methods for direct functionalization of MG, for example, via late-stage C–H functionalization, rather than laborious total synthesis.

With respect to derivatization of the indole nucleus, the methoxy group at the C9 position is synthetically accessible by selective demethylation and subsequent functionalization of the free phenol^16^. For the adjacent C10 position, a small series of compounds has been prepared^14,29^, while the C11 position remained unexplored due to the lack of functionalization methods (see below). Our preliminary docking studies suggested that relatively small substituents (e.g., F, Cl, and Me) would be tolerated at C11 (Fig. 1)^15^. However, it is presently difficult to predict the effect of such substituents on opioid receptor activation potency, efficacy, or signaling bias, demonstrating the need for C–H bond functionalization approaches compatible with the structural complexity presented by *Mitragyna* alkaloids.

More than a decade ago, we formulated the general concept of “C–H bonds as ubiquitous functionality” and demonstrated the strategic impact of C–H functionalization in both the construction of molecular frameworks and modification of existing complex cores^30–33^. For the latter, termed “complex core diversification” or “late-stage functionalization”, the synthetic power of this concept is readily apparent as the positional (as well as stereoisomeric) analogs of complex starting materials are accessed with high efficiency when compared to lengthy de novo approaches^30–36^. Late-stage functionalization approaches have since been widely adopted and become a common part of chemists’ armamentarium^37–41^. These concepts are being extended beyond C–H bonds to include the possibilities of skeleton modification via C–C and other bond activation^42–45^.

In this paper, we describe application of these concepts to *Mitragyna* alkaloids in the context of mapping their neuropharmacology. We introduce a strategic temporary modification of the MG alkaloid skeleton (“complex core restructuring”), to create a distinct chemotype disposed toward the desired C–H functionalization chemistry, namely C11 functionalization of MG’s indole nucleus. In this manner, we offer a solution that provides for rapid and selective functionalization of the aromatic ring of MG at the C11 and C12 positions (C6 and C7 positions in indole numbering). We subsequently found that these analogs enable fine-tuning of opioid receptor signaling efficacy, which represents one of the currently most promising strategies for creating safer opioid therapeutics.

## Results

### C–H borylation of MG yields C12-substituted analogs

MG is a complex natural product of a corynanthidine alkaloid type decorated with a number of functional groups, including enol ether, ester, tertiary amine, indole nitrogen, and aromatic methyl ether, arranged in a specific constitutional and geometrical configuration that underlies its opioid activity. It therefore poses an exciting challenge for late-stage functionalization. In this study, we focused on functionalization of the indole arene ring (the rationale is discussed in “Introduction”). Iridium-catalyzed arene C–H borylation was selected due to its wide substrate scope, including basic heterocyclic compounds^46,47^.

**Fig. 2:**
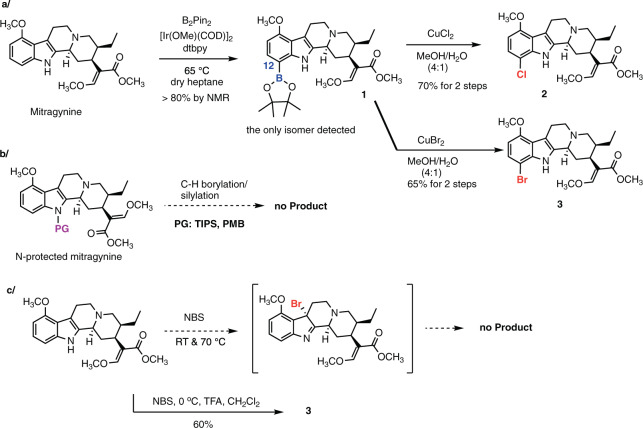
Examination of direct functionalization of mitragynine indole nucleus. **a** C–H borylation of MG provides exclusively C12-derivatives consistent with indole directing effects (C7 in indole numbering). The yields are a general range based on >10 repetitions by two experimenters. **b** The positional selectivity cannot be re-routed to C11 functionalization via previously reported TIPS protection of the indole nitrogen. **c** The C3-indole halogenation–rearrangement process^53^ is not applicable to MG. Bromination under acidic conditions gave 12-Br-MG as the major product.

After exploration and optimization of reaction conditions (Supplementary Table 1), we found that borylation is compatible with the MG chemotype. Using bis(pinacolato)diboron (B_2_Pin_2_) under catalytic [Ir(COD)OMe]_2_ and 4,4′-di-tert-butyl-2,2′-dipyridyl (dtbpy) as the ligand in absolute heptane at 80 °C for ≥17 h, C12-boronate ester **1** was obtained as the single isomer (Fig. 2a). This compound proved to be unstable and decomposed during silica gel column chromatography, and we thus confirmed its formation in the crude material via nuclear magnetic resonance spectroscopy (NMR), thin-layer chromatography (TLC), and mass spectroscopy (MS). The crude ester **1** was transformed to several derivatives without further purification in good yields. Namely, 12-Cl- (**2**) and 12-Br-MG (**3**) were prepared in two steps in 70% and 65% yield, respectively (Fig. 2a)^48^. The observed regioselectivity is consistent with the known directing effect of the indole nitrogen and C7-borylation of 2,3-disubstituted indoles^49,50^.

### Known C6 indole derivatization methods do not produce C11 analogs of MG

After achieving C12 functionalization we turned our attention to the C11 position in MG (C6 position in indole numbering), which remained unexplored due to the limited reaction repertoire available for C6 indole functionalization^51^. We considered several known approaches for C6 indole functionalization to gain access to these derivatives. Specifically, we examined the possibility of blocking the indole nitrogen’s directing effect with a protecting group, along the path demonstrated in the context of tryptophan substrates, where protection with a bulky triisopropyl silane (TIPS) group led to C6-borylation under optimized C–H borylation conditions (Fig. 2b)^52^. However, this approach was not applicable to MG as the TIPS-protected MG decomposed under the catalytic conditions. On the other hand, *p*-methoxybenzyl (PMB)-MG was either unreactive or formed undesired products under the reaction conditions. Tert-butoxycarbonyl (Boc)-protected MG provided the C11-boronate ester, but the subsequent functionalization reaction was inefficient (Supplementary Figs. 3 and 4). The latter approach was not further optimized as an entirely different protection method was found to be successful (see below).

Another avenue of enquiry was inspired by an intriguing thermal rearrangement of 3-bromoindolenines to 6-bromoindoles^53^, which had been successfully applied in complex substrates en route to stephacidine A alkaloids^42^. However, this approach also failed in the MG scaffold under similar reaction conditions (Fig. 2c); namely, MG was unreactive in the presence of *N*-bromosuccinimide (NBS) under neutral conditions, while under acidic conditions, 12-Br-MG (**3**) was the major product (Fig. 2c). Apparently, the C9 methoxy group exerts an activating and directing effect in the benzene ring of the indole nucleus once the bromination reagent is sufficiently activated^54^.

Finally, the methods relying on remote directing effect of groups attached to the indole nitrogen, such as the copper-catalyzed C6 functionalization of indoles using the phosphinimide directing group^55^, were not examined, as (1) the deprotection step involves harsh reduction conditions (e.g., LiAlH_4_) incompatible with MG’s functionalities or (2) the C6 functionalization is too restrictive in terms of the functionalization chemistry or substrate requirements^56,57^.

### Complex core restructuring: C–H borylation of mitragynine-ethylene glycol adduct (MG-EG)

Direct functionalization of MG gave C12-halo or -boronate ester analogs, while re-routing this regioselectivity was unsuccessful via either catalyst optimization or indole nitrogen protection. The known methods for C6 indole functionalization—that possess the required generality and scope to pursue systematic SAR studies—failed to provide an efficient synthetic route to C11-substituted MG analogs.

**Fig. 3:**
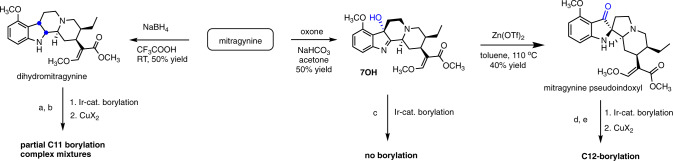
Catalytic borylation of MG analogs with reduced, oxidized, or rearranged indole nucleus. **a** B_2_Pin_2_, dtbpy/Me_4_-phen, [Ir(COD)(OMe)]_2_, 65 °C, dry heptane; 50% conversion to 11-boronate ester by ^1^H NMR. **b** CuBr_2_, MeOH/H_2_O (4:1), 80 °C; using the crude borylation mixture, provided mixtures of mitragynine, 2,3-dihydromitragynine (major products, ~60% by ^1^H NMR) and 11-Br-dihydromitragynine and 11-Br-MG (minor products, ~30% by ^1^H NMR). **c** B_2_Pin_2_, dtbpy/Me_4_-phen, [Ir(COD)(OMe)]_2_, 65 °C, dry heptane; no borylation product was detected. **d** B_2_Pin_2_, dtbpy/Me_4_-phen, [Ir(COD)(OMe)]_2_, 65 °C, dry heptane; complete conversion to C12-boronate by ^1^H NMR). **e** CuBr_2_, MeOH/H_2_O (4:1), 80 °C; no reaction.

We therefore resorted to changing the reactivity of the MG core by “removing” the indole double bond (2,3-π bond in indole numbering) by either reduction or oxidation, or oxidation followed by rearrangement (Fig. 3). 7OH was examined as an indolenine substrate in the Ir-catalyzed borylation reaction, but there was no conversion to a borylated product. 7OH can be readily rearranged to mitragynine pseudoindoxyl with zinc triflate as reported by us previously^16^, however this pseudoindoxyl system afforded C12 borylation (Fig. 3), while the corresponding 12-boronate ester (not shown) was unreactive in the subsequent halogenation reaction. Next, we investigated the possibility of using a reduced indole substrate, namely dihydromitragynine^58^. With this compound, we observed a partial conversion of starting material to the corresponding 11-boronate ester (50% conversion as determined by ^1^H NMR). However, the following halogenation reaction gave complex mixtures of MG and 2,3-dihydromitragynine as the major products, and 11-Br-MG and 11-Br-2,3-dihydromitragynine as the minor products (Fig. 3).

**Fig. 4:**
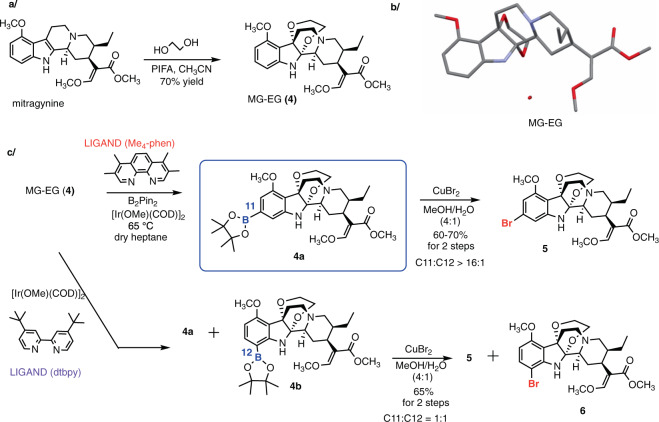
C11-selective C–H borylation via conversion of MG to mitragynine-ethylene glycol adduct (MG-EG). **a** One-step synthesis of MG-EG, a stable derivative of MG. **b** The 3D structure of MG-EG was confirmed by X-ray crystallography. ORTEP representation of MG-EG structure is shown without hydrogen atoms for clarity. **c** C–H borylation of MG-EG gives a mixture of C11- and C12-boronates and the subsequent functionalization products. The high C11 selectivity was achieved by ligand optimization. The ratio was determined by ^1^H NMR of crude reaction mixtures. The yields, given as a general range, are based on >20 repetitions by 3 experimenters, on a scale up to 100 mg of substrate.

Lastly, we focused on oxidative attachment of ethylene glycol to the indole nucleus, rendering a distinct chemotype: mitragynine-ethylene glycol (MG-EG, Fig. 4a). This unusual compound was first introduced by Takayama and co-authors to provide access to the C10-substituted analogs via electrophilic aromatic substitution, and this scaffold showed potent activity at MOR^14,59^. Aside from these studies, this indole-ethylene glycol adduct has not been explored in the context of indole chemistry. We became interested in this compound as the ethylene glycol group not only masks the indole, which is particularly relevant in this study, but also dramatically alters the shape of the entire alkaloid scaffold. We crystallized MG-EG from methanol and confirmed its 3D structure (Fig. 4b, CCDC 1905559), which revealed the chair-like conformation of the dioxane ring and the propeller-like arrangement of the three rings converging on the C–C bond of the former indole ring.

When we applied the catalytic borylation protocol (with the dtbpy ligand) to MG-EG, a 1:1 ratio of C11 (**4a**) and C12 (**4b**) products was observed (Fig. 4c), clearly confirming the different reactivity property of this chemotype, and unlocking the possibility of optimizing the reaction conditions to favor C11 functionalization.^52,60,61^ Indeed, ligand screening (Supplementary Table 3) showed that 3,4,7,8-tetramethyl-1,10-phenanthroline (Me_4_-phen) as the ligand, together with B_2_Pin_2_ and [Ir(COD)OMe]_2_ in heptane at 65 °C, gave the 11-borylated product (**4a**) as the major product with ratio >16:1 of C11 (**4a**): C12 (**4b**) products (Fig. 4c, confirmed by the subsequent bromination).

We reasoned that by converting the indole nucleus to an unusual aniline derivative, the directing effects of the nitrogen would be diminished while its steric effects would gain importance, resulting in functionalization of the unhindered C11 position (after catalyst–ligand optimization). Boronates **4a** and **4b** were converted to the bromo derivatives **5** and **6**, respectively^48^, using copper(II) bromide. Thus, this sequence provided two 11-substituted MG-EG intermediates—boronate **4a** and bromide **5**—with versatile synthetic potential.

### Preparation of C11-substituted analogs of mitragynine and related scaffolds

**Fig. 5:**
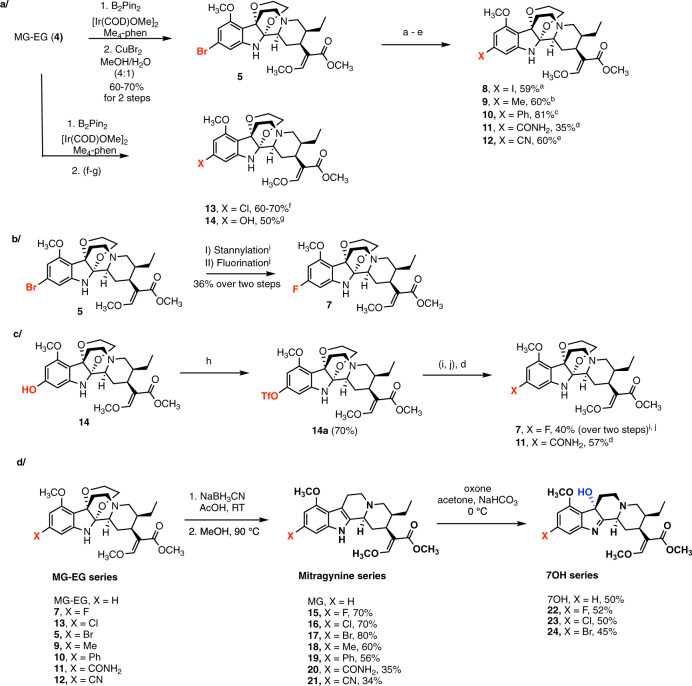
The MG-EG scaffold is compatible with a wide array of functionalization chemistries. **a**, **b**, **c** Synthesis of C11-substituted analogs of MG-EG. (a) NaI, CuI, *N*,*N* ’-dimethylethylenediamine, 110 °C, 46 h. (b) Pd_2_(dba)_3_, XPhos, DABAL-Me_3_, THF, 60 °C, 2 h. (c) Pd(dppf)Cl_2_·CH_2_Cl_2_, PhB(OH)_2_, CsOAc, THF, 70 °C, 7 h. (d) Pd(OAc)_2_, dppf, Co_2_(CO)_8_, DIPEA, NH_4_Cl, imidazole, dioxane, 90 °C, 15 h. (e) Pd_2_(dba)_3_, Zn dust, Zn(CN)_2_, [HPtBu_3_]BF_4_, DMF, RT, 3 h. (f) CuCl_2_·2H_2_O, MeOH: H_2_O (4:1), 80 °C, 12 h. (g) H_2_O_2_, THF, 0 °C to RT, 30 min. (h) PhNTf_2_, DIPEA, DMF, 50 °C, overnight. (i) (^n^Bu_3_Sn)_2_, Pd(PPh_3_)_4_, LiCl, dioxane, 100 ^°^C, overnight. (j) AgOTf, F-TEDA-PF_6_, RT, 20 min. The yields are based on >2 repetitions. **d** Reductive removal of ethylene glycol. This step renders 11-substituted MG analogs, which in turn can be oxidized to the corresponding 7OH derivatives. The yields are average based on at least 3 repetitions.

The 11-boronate ester (**4a**) was converted to the compounds 11-Cl-MG-EG (**13**) and 11-OH-MG-EG (**14**) using the appropriate substitution methods (Fig. 5a)^48,62^. For the preparation of additional derivatives, the 11-Br-MG-EG (**5**) served as the key intermediate enabling preparation of compounds **8**–**12** in one synthetic step (Fig. 5a), where X = I (**8**)^63^, Me (**9**)^64^, Ph (**10**)^65^, CONH_2_ (**11**)^66^, and CN (**12**)^67^. Considering the complexity of these compounds, the yields were satisfactory and more than sufficient to produce practical amounts of the compounds for preliminary pharmacological evaluation. However, the synthesis of 11-F-MG-EG (**7**) proved more difficult compared to its other halogen relatives. Relevant reported procedures were not effective in this context; for example, direct fluorination of 11-boronate ester **4a** using copper-mediated fluorination with (^t^BuCN)_2_CuOTf or Cu(OTf)_2_py_4_ failed^68,69^, so did an indirect sequence of stannylation–fluorination^70^. Deoxyfluorination of the corresponding phenol **14** using Phenofluor™ Mix was also unsuccessful^71^. Eventually, 11-F-MG-EG (**7**) was synthesized from bromide **5** via the sequence of stannylation and fluorination (Fig. 5b)^72^. The triflate **14a** represents an alternative intermediate (to the corresponding bromide **5**) for further functionalization; for example, (1) 11-F-MG-EG (**7**) was also synthesized from triflate through a series of stannylation and fluorination (Fig. 5c) and (2) carboxamide **11** was prepared in 57% yield (versus 35% via the bromide, Supplementary information).

The ethylene glycol moiety can be readily removed under mild reductive condition in one step to yield 11-substituted MG analogs (**15–21**, Fig. 5d) in moderate-to-good yields. Our strategy enables functionalization of the C11 position with a wide range of substituents starting directly from the MG natural product, thus permitting systematic exploration of SAR at this position.

To convert the MG analogs to the corresponding 7OH series, we optimized a set of conditions using OXONE^®^ in acetone (Fig. 5d)^73,74^. This procedure was successfully applied to the 11-substituted MG analogs, as demonstrated by preparation of the 11-halo derivatives’ **22**–**24** analogs in yields around 50%. This oxidation protocol is superior to the previously reported approaches, namely PIFA gives complex mixtures of products and a lower yield of 7OH, while lead tetraacetate involves a toxic heavy metal and requires a second hydrolysis step^75^.

In summary, we developed access to 11-substituted analogs in three MG-related molecular series, compounds previously inaccessible via electrophilic substitution or other approaches, providing the means for a systematic SAR exploration of this position in multiple MG-type scaffolds.

### Neuropharmacology: in vitro modulation of opioid receptors by the 11-substituted 7OH analogs

**Fig. 6:**
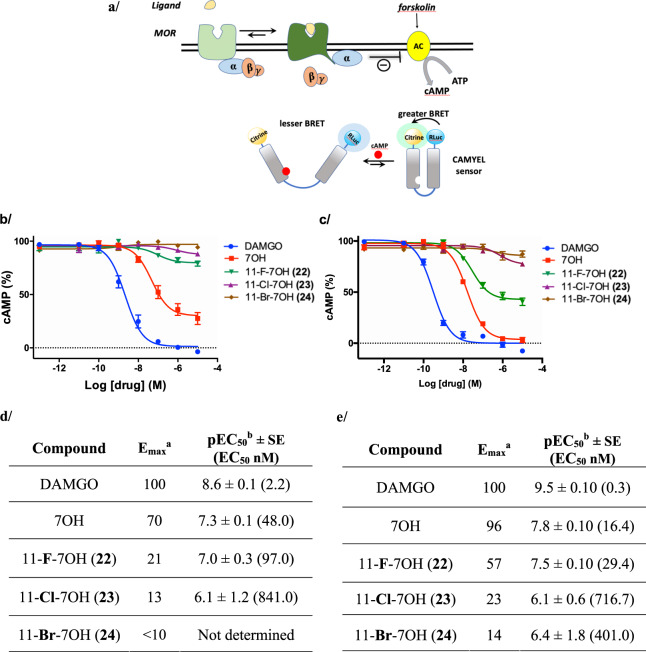
Activity of 7-hydroxymitragynine (7OH) analogs at the mu-opioid receptor (MOR). **a** Conceptual representation of the MOR CAMYEL BRET assay. To measure G protein activation, MOR (light green) was coexpressed with G protein subunits G*α*_oB_, *β*_1_, *γ*_2_, and the BRET CAMYEL sensor. Forskolin activates adenylyl cyclase (AC), which converts ATP to cyclic adenosine monophosphate (cAMP). On activation of mMOR by ligand, *α* subunit inhibits AC, resulting in a decrease of cAMP accumulation. The CAMYEL sensor is comprised of citrine (mustard yellow) and *Renilla* luciferase (light blue) with human Epac1 (blue linker) interspaced between them. **b** Agonist activity of 7OH at mMOR, presented as a percentage of cAMP inhibition produced by the positive control DAMGO ([D-Ala^2^, N-Me-Phe^4^, Gly^5^-ol]-enkephalin); **c** Agonist activity of 7OH analogs at hMOR; curves represent the average of *n* = 3, independent experiments with error bars representing ±SEM. **d** Functional activity of 7OH analogs at mMOR as determined in CAMYEL BRET assays. **e** Functional activity of 7OH analogs at hMOR as determined in CAMYEL BRET assays [^a^Agonist activity indicated by EC_50_ values, maximal efficacy is expressed as maximal inhibitory effect on cAMP levels relative to DAMGO (*E*_max_ = 100% – cAMP(min)%). ^b^All data points represent −logEC_50_ ± SE (EC_50_ nM) for *n* = 3 repetitions].

With efficient access to the C11 analogs, we examined the effect of C11 substitution on the opioid receptor pharmacology of MG analogs. We focused the initial set of biological assays on the 7OH series, as 7OH is an active metabolite of MG and exhibits an order of magnitude greater potency compared to MG as an MOR agonist^17^. First, we examined the 11-halo analogs of 7OH in radioligand binding studies to assess the affinity for the opioid receptors (Table 1). We found that the C11 halogen modulated affinity across all three opioid receptors: the 11-F compound **22** exhibited greater affinity compared to the parent 7OH, but this gain in binding was progressively lost as the halogen became larger (compounds **23** and **24**, Table 1). This effect was most pronounced at kappa-opioid receptor (KOR), where the affinity of **22** was six times greater than that of the parent compound 7OH. The compound **22** also had more than 2-fold greater affinity for delta-opioid receptor (DOR) compared to 7OH. The effect of the C11 halogen on MOR binding was subtle across the halogen series. Since the parent compound 7OH acts as a potent agonist, we examined the functional modulation of mouse MOR (mMOR) activation in living cells. Specifically, we examined the signaling consequences of G protein activation via detection of the downstream signaling molecule cyclic adenosine monophosphate (cAMP). cAMP is formed from adenosine triphosphate (ATP) by adenylyl cyclase (AC), which is inhibited by activation of the MOR and its associated G proteins. Changes in the level of cAMP were measured using a bioluminescence resonance energy transfer (BRET) functional assay. The BRET signal increases in response to decreasing amounts of cAMP as a result of conformational changes in the sensor. cAMP levels were raised prior to the assay by addition of forskolin as described previously (Fig. 6a, illustration created by the authors)^1,76^.

**Table 1:** Binding affinities of 7OH analogs at the mouse opioid receptors.

Compound	K_*i*_ ± SEM (nM)^a^		
	mMOR	mKOR	mDOR
7OH	21.5 ± 0.8	119.0 ± 2.1	88.5 ± 9.9
11-F-7OH (**22**)	13.7 ± 1.0	21.0 ± 3.2	35.8 ± 2.0
11-Cl-7OH (**23**)	27.1 ± 1.1	30.7 ± 11.4	47.2 ± 2.4
11-Br-7OH (**24**)	32.4 ± 1.4	81.1 ± 7.1	57.7 ± 6.7

^a^All data points represent mean ± SEM (nM) of *n* = 3. [^125^I]BNtxA was used as the standard radiolabeled ligand^98^.

In the cAMP assay, 7OH was a potent agonist with relatively high efficacy (*E*_max_ = 70%, compared to DAMGO ([D-Ala^2^, N-Me-Phe^4^, Gly^5^-ol]-enkephalin), EC_50_ = 48 nM, Fig. 6b, d). Introduction of the 11-fluoro substitution in compound **22** dramatically reduced the efficacy, rendering a low-efficacy agonist (*E*_max_ ~ 21%) (Fig. 6b, d). For the **23** and **24** derivatives, the agonist activity at mMOR was nearly abolished (*E*_max_ = 13% for **23** and *E*_max_ < 10% for **24**). Accordingly, 11-halo substituents in the 7OH scaffold do not interfere with binding of these compounds to mMOR, but profoundly modulate the signaling efficacy at this receptor.

To determine if the efficacy modulation trend holds at the human receptor, we examined the 11-X-7OH series in hMOR-CAMYEL assay (Fig. 6c) using the same experimental setup as illustrated above (Fig. 6a). The parent compound 7OH was potent and highly efficacious (*E*_max_ = 96%, compared to the reference ligand DAMGO, EC_50_ = 16.4 nM, Fig. 6c, e), whereas 11-F-7OH (**22**) was a partial agonist with markedly reduced efficacy (*E*_max_ = 57%, EC_50_ = 29.4 nM, Fig. 6c, e). The **23** and **24** derivatives showed very low efficacy (*E*_max_ = 23% for **23** and *E*_max_ = 14% for **24**). Hence, we can conclude that the trend in efficacy signaling modulation in the halogen 7OH series is comparable between human and mouse MOR.

**Fig. 7:**
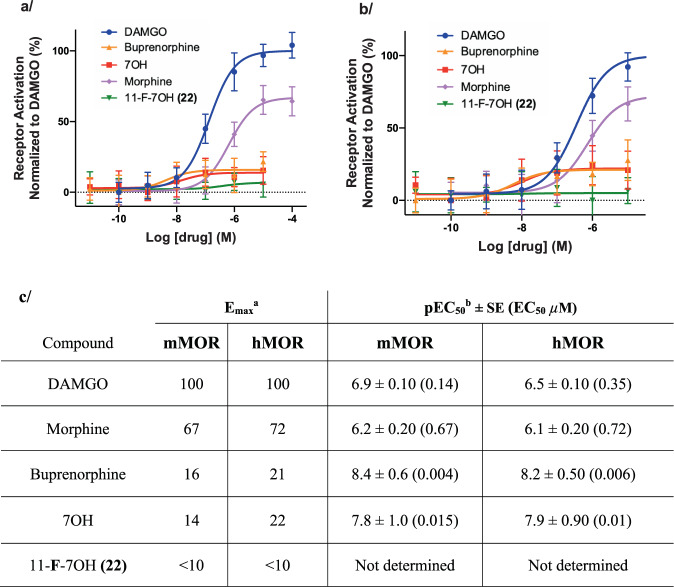
Receptor activation by 7OH series in comparison to known opioids in BRET-based Nb33 recruitment assay at mMOR and hMOR. Intrinsic receptor activation efficacy was determined by the extent of recruitment of the conformationally selective nanobody Nb33 to hMOR or mMOR fused as its C terminus to NanoLuc, as measured by BRET. Positive controls used are: DAMGO, buprenorphine, morphine, and parent 7OH. **a** Agonist activity of 11-F-7OH (**22**) as compared to positive controls at mMOR. **b** Agonist activity of 11-F-7OH (**22**) as compared to positive controls at hMOR. Curves represent the average of *n* = 3, independent experiments with error bars representing ±SEM. **c** Functional activity of 11-F-7OH (**22**) and positive controls at mMOR and hMOR in Nb33 BRET Assay [^a^Agonist activity indicated by EC_50_ values, maximal efficacy is expressed relative to DAMGO (*E*_max_). ^b^All data points represent −logEC_50_ ± SE (EC_50_ µM) for *n* = 3 repetitions].

The measured efficacy of receptor agonists depends greatly on the receptor reserve pools and the extent of downstream signaling amplification of each functional readout. Under the conditions typically used in in vitro cell-based assays, such as the cAMP BRET assay discussed above, the efficacy of partial agonists appear exaggerated owing to large receptor pools produced by receptor overexpression and downstream signaling amplification^77^. To examine this effect for 7OH and its analogs, we adopted an assay that detects an active conformation of MOR, using the Nb33 nanobody sensor, and thus limits the signaling-related amplification (Nb33 BRET assay using a luciferase-tagged MOR and Venus-tagged Nb33, see Supplementary information)^78^. This assay was initially described by Stoeber et al.^79^ and has recently been rigorously validated by Gillis et al.^78^ via comparison to a number of independent signaling assays, and calibrated by direct comparison of several opioid drugs including well-established partial agonists (buprenorphine and morphine) and experimental opioids. Using mMOR and hMOR Nb33-BRET assays, we found that morphine and buprenorphine act as partial agonists, in comparison to DAMGO, with markedly different signaling efficacies (Fig. 7). While morphine appears as a partial agonist with relatively high efficacy (*E*_max_ ~ 67% (mMOR) and 72% (hMOR), Fig. 7), buprenorphine shows much lower efficacy (*E*_max_ ~16% (mMOR) and 21% (hMOR)), results consistent with the previous report^78^. Remarkably, we found that 7OH exhibits low “intrinsic” efficacy (*E*_max_ ~14% (mMOR) and 22% (hMOR), Fig. 7) comparable to that of buprenorphine. In direct comparison, the efficacy of **22** is further reduced to near the detection limit of the assay using both mMOR and hMOR (Fig. 7).

The two assays employed provide complementary windows into MOR signaling efficacy measurements. The Nb33 BRET assay, with limited signaling amplification, positioned 7OH close to buprenorphine in terms of the broad range of signaling efficacies of different opioids. Despite the narrow dynamic range of this assay for low-efficacy compounds, it showed that fluorination of the 11-position further impaired the signaling efficacy of 7OH to near the limit of detection. In the amplified cAMP CAMYEL BRET assay, with a greater dynamic range in the low end of efficacy readout, **22** exhibits greatly diminished but readily detectable agonist signaling as compared to 7OH, whereas the efficacy was essentially lost for the much less efficacious compounds **23** and **24**.

**Fig. 8:**
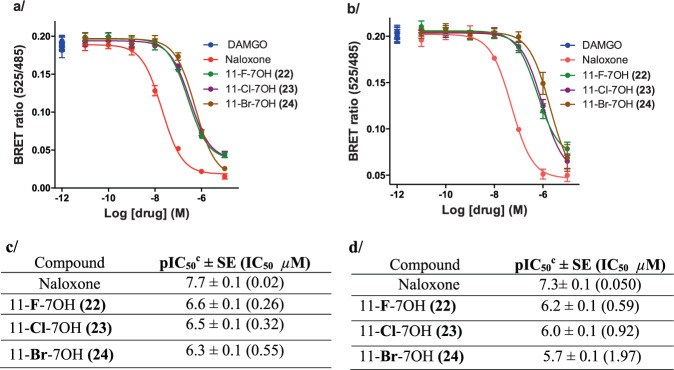
Antagonist activity of 7OH analogs at mMOR and hMOR. To assay G protein activation, mMOR and hMOR was coexpressed with G protein subunits G*α*_oB_, *β*_1_, *γ*_2_, and the BRET CAMYEL sensor. Antagonism was measured by the inhibition of DAMGO’s effect on cAMP. **a** Competitive antagonist activity of 7OH analogs at mMOR; positive control = naloxone. **b** Competitive antagonist activity of 7OH analogs at hMOR; positive control = naloxone. Curves represent the average of *n* = 3, independent experiments with error bars representing ±SEM. **c** Functional antagonist activity of 7OH analogs at mMOR as determined in CAMYEL BRET assays. **d** Functional antagonist activity of 7OH analogs at hMOR as determined in CAMYEL BRET assays [^c^Antagonist activity indicated by IC_50_ values for inhibition of a reference agonist (DAMGO), all data points represent −logIC_50_ ± SE (IC_50_ µM) for *n* = 3 repetitions].

To correlate the very low agonist efficacy with antagonism, we next determined the antagonist activity of the 11-X-7OH series in a CAMYEL antagonism assay at mMOR (Fig. 8a, c) and hMOR (Fig. 8b, d) compared to the positive control naloxone. As expected, the compounds were able to inhibit the response elicited by the standard full agonist DAMGO at hMOR and mMOR down to the levels predicted based on their agonist efficacy as determined above.

To complete the examination of this series at the opioid receptors, we performed the analogous cAMP BRET assays with mDOR and hDOR, and rat KOR (rKOR) and hKOR. The parent 7OH was found to act as a partial agonist at both mDOR (*E*_max_ = 72%, EC_50_ = 71.4 nM) and hDOR (*E*_max_ = 45%, EC_50_ = 99.9 nM, Supplementary Fig. 7)^80^. Again, **22** showed considerable reduction in signaling efficacy at the receptors of both species, when compared to 7OH, without a major shift in potency (*E*_max_ = 41%, EC_50_ = 102.1 nM at mDOR; *E*_max_ = 26%, EC_50_ = 150.8 nM at hDOR,). The **23** and **24** compounds had very low efficacy at mDOR and hDOR (Supplementary Fig. 7). Thus, the C11 halogen trend described for MORs was also observed at DORs. In contrast, this compound series showed no agonist activity at the KOR receptors (Supplementary Fig. 8). Finally, we selected one representative compound of this series, 11-Br-7OH **(24)**, and tested it for antagonism at the KOR and DOR (Supplementary Figs. 9 and 10) revealing full inhibition of reference agonists DPDPE ([D-Pen^2,5^]-enkephalin) for DOR and U50,488 for KOR.

In summary, these pharmacological studies indicate an important role for the C11 substitution in selective and profound modulation of the signaling efficacy at MORs and DORs of the MG-related scaffolds and provide a strong rationale for examining this effect in vivo.

### 11-Fluoro-7-hydroxymitragynine (**22**) is a low-efficacy partial agonist in vivo in mouse analgesia tests

**Fig. 9:**
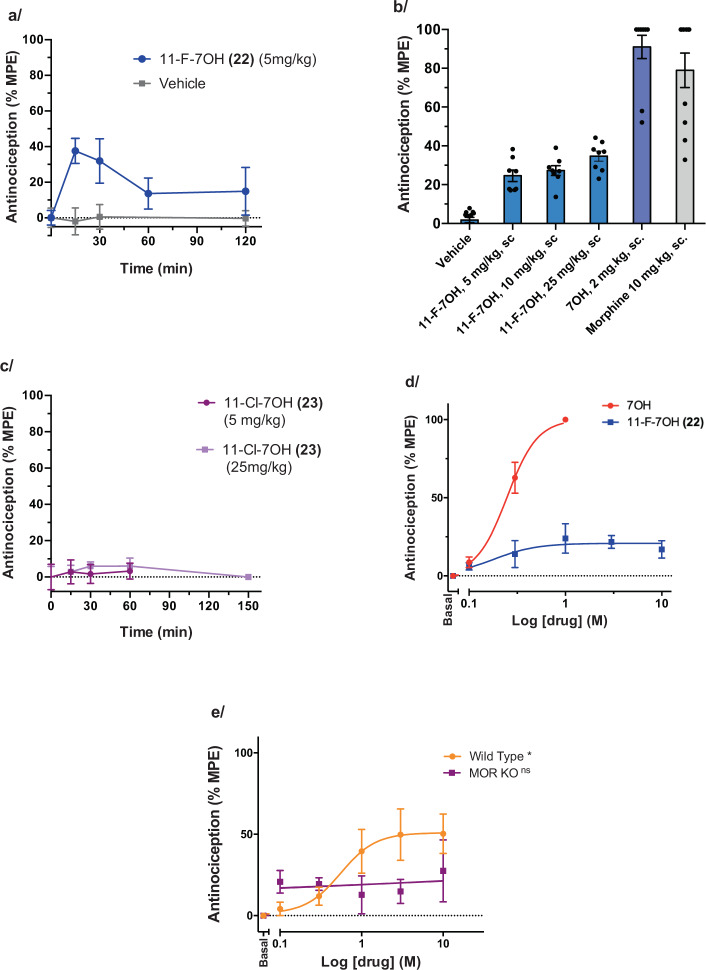
Antinociceptive effects of 7OH analogs in tail-flick assay in mice. **a** Time course of the antinociceptive effect of **22** (5 mg/kg. s.c., *n* = 11 CD1 mice) and vehicle (*n* = 6 CD1 mice) in the tail-flick assay. Each point represents mean ± SEM. % MPE, a percentage of the maximum possible effect as set in this assay (see Supplementary information). **b** Tail-flick dose–response for the high-dose range (5, 10, and 25 mg/kg, s.c.; *n* = 8 CD1 mice each group) of **22**, in direct comparison to controls 7OH and morphine. CD1 mice (*n* = 10), at peak analgesic time point. (Vehicle (−5.4, 7.8, 4.0), 11-F-7OH 5 mg/kg (17.3, 22.4, 38.3), 11-F-7OH 10 mg/kg (13.7, 39.0, 27.1), 11-F-7OH 25 mg/kg (23.1, 44.2, 36.8), 7OH 2 mg/kg (52.1, 100.0, 100.0), Morphine 10 mg/kg (32.8, 100, 100)) **c** Time course of **23** at 2 doses (5 and 25 mg/kg, s.c., *n* = 8 CD1 mice each). Each point represents mean ± SEM. **d** Dose–response curves in mouse tail-flick assay for the parent 7OH (s.c.) and **22** (s.c.), 15 min time point, C57BL/6 mice (*n* = 10). Each point represents mean ± SEM. **e** Antinociceptive effect of 11-F-7OH (**22**) is MOR dependent. Dose–response curves of **22** (s.c.), in the tail-flick assay was evaluated 15 min post drug administration in WT, C57 mice (*n* = 8) and MOR KO mice (*n* = 5). Each point represents mean ± SEM. One-way RM ANOVA shows statistically significant result for WT* mice with *p = 0.006 and statistically non-significant result (MOR KO^ns^) with *p* = 0.832 for MOR KO mice.

We set out to examine how the gradual signaling efficacy modulation found in vitro, within the 7OH series, would translate to in vivo effects in living rodents. There is a strong rationale for pursuing such studies, as partial MOR agonists may provide a path to safer opioid therapeutics (see “Discussion” below for more details). We recently reported that 7OH was a potent and efficacious analgesic in mice using a tail-flick assay (thermal nociception assay)^16,17^, consistent with a previous report by others^81,82^. On the basis of the previously determined ED_50_ value for 7OH in mouse tail-flick assay (ED_50_ = 0.57 (0.19–1.7) mg/kg, 95% CI)^17^, and similar in vitro binding and signaling potencies of 7OH and **22** at MOR, we selected a relatively high dose of **22** (5 mg/kg, subcutaneous (s.c.) administration) for the initial time course profiling of the analgesic effect in CD1 mice (Fig. 9a). The analgesic effect peaked at 15 min post injection, indicating rapid bioavailability in vivo comparable to the parent 7OH, followed by a decay to a residual analgesic effect that lasted for at least 120 min. Noteworthy is the partial analgesic efficacy of <40% MPE (maximum possible effect) in the tail-flick assay. To confirm that the maximum effect was reached, we examined even higher doses at a 15 min time point, namely 10 and 25 mg/kg (s.c.), which showed that indeed the maximal analgesic effect was reached already at the 5 mg/kg dose (Fig. 9b). The control compounds, 7OH and morphine, elicited a greater analgesic effect in the same mouse strain. In contrast, **23** showed no analgesic effect at 5 or 25 mg/kg dose (Fig. 9c).

Thus, the in vitro MOR pharmacological profile of the 7OH analogs translates well to the in vivo tail-flick test, an established physiological readout for MOR agonists that measures antinociceptive effects of drugs to painful thermal stimuli. We next set out to rigorously confirm these results by: (1) using a different and widely used mouse strain, C57BL/6J mice; (2) performing the experiments in a different location and research group; and (3) determining a full dose-curve for both the control (7OH) and the compound **22** (Fig. 9d). 7OH showed a potent and high-efficacy analgesic effect (ED_50_ = 0.25 (0.20–0.31) mg/kg, 95% CI, analgesic efficacy normalized to 100%, comparable to morphine), consistent with previous results^17^, while **22** also elicited a potent analgesic effect (ED_50_ = 0.18 (0.04–0.78) mg/kg, 95% CI), but with dramatically attenuated analgesic efficacy (*E*_max_ = 21%, Fig. 9d). Thus, **22** is equipotent to 7OH but exhibits low efficacy in antinociceptive effects, whereas 7OH is comparable to morphine in efficacy.

To confirm that the low-efficacy analgesia is driven by MOR, and no other targets or mechanisms (e.g., other opioid receptors or non-opioid off-targets), we performed the mouse tail-flick assay in wild-type (WT), and MOR knockout (MOR KO) mice with the same genetic background (Fig. 9e). Despite the low range of the efficacy readout afforded by **22**, we were able to show that the dose-dependent effect of **22** was statistically significant in WT mice, but not in MOR KO animals.

These results support an explanatory model where the analgesic effect of 11-F-7OH is largely driven by MOR, and the reduced analgesic efficacy is a consequence of low-efficacy MOR activation/signaling induced by this compound. Thus, 7OH and 11-F-7OH provide an excellent probe pair for examining the effects of relative signaling efficacy on therapeutic (desired) and adverse effects, and the resulting preclinical measure of a therapeutic window.

## Discussion

Most recent estimates of kratom use, based on the tonnage of imported kratom and survey reports of dosing ranges, suggest 10–16 million users in the US alone^83^. Numerous anecdotal reports, including those collected by the United States Drug Enforcement Administration (>23,000 publicly available reports)^8^, point to remarkable medicinal effects of kratom in self-medication for chronic pain, depression, anxiety, substance use disorders, and other ailments. Inspired by these clinical indicators, our laboratories have been studying the chemistry and biology of kratom alkaloids with the long-term goal of developing drug leads based on these compounds. In this context, the aromatic ring SAR of MG has not been mapped systematically, as only limited series of analogs have been synthesized and studied (e.g., C10 analogs)^14,16^, and the C11 position has not been examined at all. To address this task, and to complement our total synthesis efforts^15^, we set out to examine the late stage functionalization of MG, in this study focusing on the C11 position of the indole nucleus.

Years ago, we formulated the ideas of C–H bond functionalization and late-stage functionalization as general concepts with transformational potential for the thought and practice of chemical synthesis of carbon-based substances^30–36^. These concepts have since been widely adopted in both academia and industry and the C–H functionalization mindset, as well as corresponding methods, are indispensable components of today’s synthetic repertoire^37–40^. Our team has been developing methods for systematic and programmable functionalization of heteroarenes, which are essential building blocks in medicinal chemistry, including indoles, pyrroles, pyrazoles, imidazoles, pyridines, and others^30–36^. In the present study, we focused on the benzene ring of highly complex indoles, in the context of specific neuropharmacological goals.

Our approach provided a practical route to C11-analogs of MG, 7OH, and MG-EG, from the natural product, and systematic SAR exploration of the C11 position. The results indicate that substitution of this position plays an important role with respect to MOR signaling, specifically in modulating the efficacy of receptor-triggered signaling events. Designing the receptor signaling parameters of GPCR ligands a priori remains an unfulfilled potential of computational methods, and thus, a systematic examination of functional characteristics of receptor ligands and probes still relies largely on chemical synthesis. The chemical approach described here provides a means to advance our examination and understanding of how molecular interactions of MG-type scaffolds with the opioid receptors underlie functional outcomes such as G protein signaling and maximal signaling efficacy.

There has been considerable excitement and hope for generating MOR agonists that bias its signaling toward G protein-initiated pathways as a means for rational design of improved safety and therapeutic index of opioid analgesics (“G protein bias hypothesis” of opioids)^84^. It was suggested that some of the typical adverse effects of opioids such as respiratory depression or reinforcing effects are linked to arrestin signaling. According to this approach, biasing the MOR-triggered signaling toward the G protein and away from arrestin would result in attenuation of the adverse effects, relative to the desired analgesic activity^84^. However, this hypothesis has been seriously challenged using both genetic and pharmacological tools^78,85–90^. An emerging alternative hypothesis is that partial agonism of MOR can lead to reduced side effects while preserving therapeutic effects (“signaling efficacy hypothesis”)^78,91^. An example is the superior safety profile of buprenorphine (a partial agonist in vitro) compared to the standard prescription opioids^92^. Buprenorphine is as efficacious as full agonists in treating pain but has substantially reduced ability to produce respiratory depression and thus reduced risk of opioid overdose death^92^. Such low-efficacy compounds may have been incorrectly identified as G protein biased due both to receptor reserve and to the much greater amplification of the G protein signaling arm as compared to the much less sensitive arrestin pathway assays^78^. Lower efficacy opioids are also of great interest for the treatment of opioid use disorder (OUD) where such compounds may provide efficacious opioid maintenance while having a reduced abuse potential. Therefore, close structural derivatives with a varying degree of G protein signaling efficacy are needed for direct and rigorous probing of this hypothesis.

In this context the 11-halo series of 7OH provide valuable pharmacological tools. We demonstrated that 7OH is a potent low-efficacy agonist at mMOR and hMOR in the efficacy range comparable to buprenorphine (*E*_max_ ~ 20% versus DAMGO), in a cell-based assay without signaling amplification (Nb33 BRET assay). Comparatively, 11-F-7OH showed only minimal receptor activation in this assay. In the amplified system (cAMP BRET assay) with a greater dynamic range on the low end of efficacy spectrum, the enhancement in apparent efficacy of 7OH was much smaller for **22**. Thus, using two assays with complementary dynamic ranges of efficacy readouts, we showed that fluorination in the 11-position markedly reduces MOR signaling efficacy of an already low-efficacy agonist, the parent 7OH.

Interestingly, the correlation between the cAMP signaling efficacy obtained in vitro in cultured cells and the in vivo analgesic efficacy determined in living animals, for the 11-X-7OH series, suggests that the in vitro amplified system may mimic the receptor reserve and/or receptor-effector situation in the neurons of relevant pain circuitry, and thus provides a useful model system. Considering the recent surveys that document much improved safety of kratom compared to the prescription or illicit opioids^83,93,94^, a further decrease of MOR’s G protein signaling efficacy of the key mediator of kratom’s opioid-like effects, 7OH^17^, may lead to even safer compounds, and illustrates the potential importance of the present work. Further, a recent report showed that 7OH attenuated alcohol intake in drinking mice and that this effect was mediated by DOR activation, while 7OH exhibited rewarding effects on its own^80^. Thus, the attenuated MOR signaling efficacy of **22**, while maintaining sufficient DOR signaling, may provide a therapeutic lead for OUD and other substance use disorders with no or much diminished abuse potential. That we see only partial analgesia for **22** and effective analgesia with 7OH suggests that the ideal efficacy may be somewhere between that of these two analogs, which can guide subsequent synthetic efforts in search of the ideal balance between therapeutic effects and side effects.

In summary, we describe a systematic examination of late-stage functionalization of kratom alkaloids, which provided efficient access to MG analogs and identified 11-F-7OH (**22**) as an important lead compound for further investigations.

## Methods

### General procedure for synthesis of 11-boronate ester MG-EG (4a)

Starting material MG-EG (**4**) (50 mg, 0.11 mmol), [Ir(COD)OMe]_2_ (3.6 mg, 5.5 µmol, 5 mol%), 3,4,7,8-tetramethyl-1,10-phenanthroline (3.9 mg, 16 µmol, 15 mol%) and B_2_Pin_2_ (111 mg, 0.44 mmol, 4 equiv.) were balanced into an oven dried vial. The vial was purged with argon, dry heptane (2.5 mL) was added under argon, and the vial was sealed with a Teflon-lined screw cap and heated to 65 °C. The RM became a dark red-brown solution after 5–15 min of heating. After 17–24 h, when LR-MS indicated complete consumption of SM, the RM was concentrated to give the crude boronate ester. This intermediate was immediately used to prepare the **5**, **13**, and **14** derivatives without further purification. Compound **7** was synthesized either from **14** through a sequence of triflation, stannylation and fluorination or from **5** through a sequence of stannylation and fluorination.

### General procedure for synthesis of 11-X-MG derivatives (15–17)

Starting material (**5**, **7**, or **13**, 0.11 mmol) was dissolved in AcOH (2.0 mL) under argon and NaBH_3_CN (13.7 mg, 0.22 mmol, 2 equiv.) was added to the solution. After stirring at RT for 15 min, another portion of NaBH_3_CN (13.7 mg, 0.22 mmol, 2 equiv.) was added and stirring was continued for 1 h. After this time, MeOH (81 *µ*L) was added and the RM was heated to 90 °C for 1 h (for **15**) and 14 h (for **16** and **17**). The reaction mixture was added into a cold concentrated NH_4_OH solution and extracted with DCM. After drying over Na_2_SO_4_, the DCM extract was evaporated. Product was purified by preparative TLC using an appropriate solvent mixture.

### General procedure for synthesis of 11-X-7OH derivatives (**22–24**)

Starting material (**15**, **16**, or **17**; 73 µmol) was dissolved in acetone (2.2 mL), sat. aq. NaHCO_3_ (1.5 mL) was added, and the stirred suspension was cooled in an ice bath (0 °C). OXONE^®^ (1.4–1.5 equiv.) in H_2_O (0.7 mL) was added dropwise over 20 min with vigorous stirring (care should be taken that the RM does not form lumps and should be stirred thoroughly). The reaction was monitored during the addition of OXONE^®^ by TLC. After 25 min from the first addition, the reaction mixture was diluted with H_2_O (10 mL) and extracted with EtOAc (3 × 10 mL). The combined extracts were washed with brine (10 mL), dried over Na_2_SO_4_, and concentrated. Product was purified by preparative TLC using an appropriate solvent mixture to synthesize compounds **22**, **23**, and **24**.

### Tail-flick mice assay

#### Mice

For analgesic dose–response experiments, male CD1 mice (20–32 g), 6–8 weeks were obtained from Charles River Laboratories and male C57BL/6 mice (22–30 g), 8–15 weeks were obtained from Jackson Lab (Bar Harbor, ME) and housed 5 mice per cage in a vivarium following an IACUC-approved protocol. For male C57BL/6 mice temperature was kept constant at 22 ± 2 °C, and relative humidity was maintained at 50 ± 5%. For male CD1 mice (20–32 g) the temperature was in the range of 20–26 °C and relative humidity maintained within the range of 30–70%. Mice were given access to food and tap water ad libitum. All mice used throughout the manuscript were opioid naïve. All mice were maintained on a 12 h light/dark cycle with Purina rodent chow and water available ad libitum and housed in groups of five until testing.

For analgesic testing in KO animals, wild-type, male C57BL/6 mice (22–33 g), 10–12 weeks were purchased from the Jackson Lab (Bar Harbor, ME). These mice were kept at a constant temperature of 22 ± 2 °C, and relative humidity was maintained at 40–50%. Exon-1/Exon-11 MOR-1 KO mice on a C57 background were bred in the Pintar laboratory at Rutgers University. All mice were maintained on a 12-hour light/dark cycle with food and water available ad libitum, and housed in groups of five until testing. All testing was done in the light cycle.

All animal studies were preapproved by the Institutional Animal Care and Use Committees of Washington University School of Medicine and Columbia University, in accordance with the 2002 National Institutes of Health Guide for the Care and Use of Laboratory Animals.

#### Tail flick (dose–response)

Tail-flick antinociception was determined using the radiant heat tail-flick technique using an Ugo Basile model 37360 instrument as previously described^95,96^. The intensity was set to achieve a baseline between 2 and 3 s. Baseline latencies were determined before experimental treatments for all mice. Tail-flick antinociception was assessed as an increase in baseline latency, with a maximal 15 s latency to minimize damage to the tail. Data were analyzed as percent maximal effect, % MPE, and was calculated according to the formula: % MPE = [(observed latency – baseline latency)/(maximal latency – baseline latency)] × 100. Compounds were injected s.c. and antinociception was assessed at the peak effect. Mice were tested for analgesia with cumulative subcutaneous doses of the drug until the mouse can withstand the maximal latency. Once the mouse reached the maximal latency, the mouse was no longer given higher doses. The analgesia experiments were performed by blinding the experimenter to the identity of 7OH versus 11-F-7OH. In vivo experiments were evaluated using GraphPad Prism 8, San Diego, CA as described above.

#### Tail flick (MOR KO animals)

Analgesia was tested in wild-type and MOR KO animals by the radiant heat tail-flick technique using an IITC Model 33 Tail Flick Analgesia Meter as previously described^97^. The intensity was set to achieve a baseline between 2 and 3 s. Tail-flick antinociception was assessed as an increase in baseline latency, with a maximal 10 s latency to minimize damage to the tail. Data were analyzed as percent maximal effect, % MPE, which was calculated according to the formula: % MPE [(observed latency − baseline latency)/(maximal latency − baseline latency)] × 100. Compounds were administered s.c. as indicated in the figures, and analgesia was assessed at the peak effect (15 min). Mice were tested for analgesia with cumulative subcutaneous doses of the drug until the mouse can withstand the maximal latency. Once the mouse reached the maximal latency, the mouse was no longer given higher doses. In vivo experiments were evaluated using GraphPad Prism 8, San Diego, CA as described above.

### Reporting summary

Further information on research design is available in the Nature Research Reporting Summary linked to this article.

## Supplementary information

Supplementary Information

Reporting Summary

## Data Availability

The authors declare that all the data supporting the findings of this study are available within the article and Supplementary Information files which contains synthetic procedures and NMR spectra for the featured compounds, additional functional data at rodent and human receptors, biological protocols for receptor binding, activity and antinociception assays. The X-ray crystallographic coordinates for structure **4** reported in this study have been deposited at the Cambridge Crystallographic Data Centre (CCDC), under deposition numbers 1905559. This data can be obtained free of charge from The Cambridge Crystallographic Data Centre via www.ccdc.cam.ac.uk/data_request/cif. A preprint version of this work has been deposited in the public depository ChemRxiv platform 10.26434/chemrxiv.12799787. Source data are provided with this paper.

## Source data

Source Data

## References

[1] Gassaway MM, Rives M-L, Kruegel AC, Javitch JA, Sames D (2014). The atypical antidepressant and neurorestorative agent tianeptine is a μ-opioid receptor agonist. Transl. Psychiatry.

[2] Kruegel AC, Rakshit S, Li X, Sames D (2015). Constructing *Iboga* alkaloids via C–H bond functionalization: examination of the direct and catalytic union of heteroarenes and isoquinuclidine alkenes. J. Org. Chem..

[3] Gassaway MM (2016). Deconstructing the *Iboga* alkaloid skeleton: potentiation of FGF2-induced glial cell line-derived neurotrophic factor release by a novel compound. ACS Chem. Biol..

[4] Marton S (2019). Ibogaine administration modifies GDNF and BDNF expression in brain regions involved in mesocorticolimbic and nigral dopaminergic circuits. Front. Pharmacol..

[5] Adkins JE, Boyer EW, McCurdy CR (2011). *Mitragyna speciosa*, a psychoactive tree from Southeast Asia with opioid activity. Curr. Top. Med. Chem..

[6] Kruegel AC, Grundmann O (2018). The medicinal chemistry and neuropharmacology of kratom: a preliminary discussion of a promising medicinal plant and analysis of its potential for abuse. Neuropharmacology.

[7] Swogger MT (2015). Experiences of kratom users: a qualitative analysis. J. Psychoactive Drugs.

[8] DEA 3 Factor Analysis for mitragynine and 7-hydroxymitragynine https://www.regulations.gov/document?D=DEA-2016-0015-0004 (August 2016).

[9] Swogger MT, Walsh Z (2018). Kratom use and mental health: a systematic review. Drug Alcohol Depend..

[10] Fluyau D, Revadigar N (2017). Biochemical benefits, diagnosis, and clinical risks evaluation of kratom. Front. Psychiatry.

[11] Chakraborty S, Majumdar S (2021). Natural products for the treatment of pain: chemistry and pharmacology of salvinorin A, mitragynine, and collybolide. Biochemistry.

[12] Wilson LL (2021). Kratom alkaloids, natural and semi-synthetic, show less physical dependence and ameliorate opioid withdrawal. Cell Mol. Neurobiol.

[13] Takayama H (2004). Chemistry and pharmacology of analgesic indole alkaloids from the rubiaceous plant, *Mitragyna speciosa*. Chem. Pharm. Bull..

[14] Takayama H (2006). New procedure to mask the 2,3-π bond of the indole nucleus and its application to the preparation of potent opioid receptor agonists with a corynanthe skeleton. Org. Lett..

[15] Kruegel AC (2016). Synthetic and receptor signaling explorations of the *Mitragyna* alkaloids: mitragynine as an atypical molecular framework for opioid receptor modulators. J. Am. Chem. Soc..

[16] Váradi A (2016). Mitragynine/corynantheidine pseudoindoxyls as opioid analgesics with Mu agonism and delta antagonism, which do not recruit *β*-arrestin-2. J. Med. Chem..

[17] Kruegel AC (2019). 7-Hydroxymitragynine is an active metabolite of Mitragynine and a key mediator of its analgesic effects. ACS Cent. Sci..

[18] Macko E, Weisbach JA, Douglas B (1972). Some observations on the pharmacology of mitragynine. Arch. Int. Pharmacodyn. Ther..

[19] Hemby SE, McIntosh S, Leon F, Cutler SJ, McCurdy CR (2019). Abuse liability and therapeutic potential of the *Mitragyna speciosa* (kratom) alkaloids mitragynine and 7-hydroxymitragynine: kratom abuse liability. Addict. Biol..

[20] Yue K, Kopajtic TA, Katz JL (2018). Abuse liability of mitragynine assessed with a self-administration procedure in rats. Psychopharmacology.

[21] Volkow ND, Collins FS (2017). The role of science in addressing the opioid crisis. N. Engl. J. Med..

[22] Majumdar S, Devi LA (2018). Strategy for making safer opioids bolstered. Nature.

[23] Samuels BA (2017). The behavioral effects of the antidepressant tianeptine require the Mu-opioid receptor. Neuropsychopharmacology.

[24] Takayama H (1995). The first total synthesis of (−)-mitragynine, an analgesic indole alkaloid in *Mitragyna speciosa*. Tetrahedron Lett..

[25] Ma J, Yin W, Zhou H, Liao X, Cook JM (2009). General approach to the total synthesis of 9-methoxy-substituted indole alkaloids: synthesis of mitragynine, as well as 9-methoxygeissoschizol and 9-methoxy-*N*_b_-methylgeissoschizol. J. Org. Chem..

[26] Kerschgens IP (2012). Total syntheses of mitragynine, paynantheine and speciogynine via an enantioselective thiourea-catalysed Pictet–Spengler reaction. Chem. Commun..

[27] Kim J, Schneekloth JS, Sorensen EJ (2012). A chemical synthesis of 11-methoxy mitragynine pseudoindoxyl featuring the interrupted Ugi reaction. Chem. Sci..

[28] Sun X, Ma D (2011). Organocatalytic approach for the syntheses of corynantheidol, dihydrocorynantheol, protoemetinol, protoemetine, and mitragynine. Chem. Asian J..

[29] Matsumoto K (2014). Orally active opioid / dual agonist MGM-16, a derivative of the indole alkaloid Mitragynine, exhibits potent antiallodynic effect on neuropathic pain in mice. J. Pharmacol. Exp. Ther..

[30] Godula K (2006). C–H bond functionalization in complex organic synthesis. Science.

[31] Pastine SJ, Gribkov DV, Sames D (2006). sp^3^ C−H bond arylation directed by amidine protecting group: α-arylation of pyrrolidines and piperidines. J. Am. Chem. Soc..

[32] Guo P, Joo JM, Rakshit S, Sames D (2011). C–H arylation of pyridines: high regioselectivity as a consequence of the electronic character of C–H bonds and heteroarene ring. J. Am. Chem. Soc..

[33] Genovino J, Sames D, Hamann LG, Touré BB (2016). Accessing drug metabolites via transition-metal catalyzed C−H oxidation: the liver as synthetic inspiration. Angew. Chem. Int. Ed..

[34] Lane BS, Brown MA, Sames D (2005). Direct palladium-catalyzed C-2 and C-3 arylation of indoles: a mechanistic rationale for regioselectivity. J. Am. Chem. Soc..

[35] Goikhman R, Jacques TL, Sames D (2009). C−H bonds as ubiquitous functionality: a general approach to complex arylated pyrazoles via sequential regioselective *C*-arylation and *N*-alkylation enabled by SEM-group transposition. J. Am. Chem. Soc..

[36] Wang X, Lane BS, Sames D (2005). Direct *C*-arylation of free (NH)-indoles and pyrroles catalyzed by Ar−Rh(III) complexes assembled in situ. J. Am. Chem. Soc..

[37] He J, Wasa M, Chan KSL, Shao Q, Yu J-Q (2017). Palladium-catalyzed transformations of alkyl C–H bonds. Chem. Rev..

[38] McMurray L, O’Hara F, Gaunt MJ (2011). Recent developments in natural product synthesis using metal-catalysed C–H bond functionalisation. Chem. Soc. Rev..

[39] Cernak T, Dykstra KD, Tyagarajan S, Vachal P, Krska SW (2016). The medicinal chemist’s toolbox for late stage functionalization of drug-like molecules. Chem. Soc. Rev..

[40] Yang L, Huang H (2015). Transition-metal-catalyzed direct addition of unactivated C–H bonds to polar unsaturated bonds. Chem. Rev..

[41] Gensch T, Hopkinson MN, Glorius F, Wencel-Delord J (2016). Mild metal-catalyzed C–H activation: examples and concepts. Chem. Soc. Rev..

[42] Mukai K (2017). Bioinspired chemical synthesis of monomeric and dimeric stephacidin A congeners. Nat. Chem..

[43] Kerschgens I, Rovira AR, Sarpong R (2018). Total synthesis of (−)-xishacorene B from (*R*)-carvone using a C–C activation strategy. J. Am. Chem. Soc..

[44] Roque JB, Kuroda Y, Göttemann LT, Sarpong R (2018). Deconstructive fluorination of cyclic amines by carbon–carbon cleavage. Science.

[45] Liao K (2017). Site-selective and stereoselective functionalization of non-activated tertiary C–H bonds. Nature.

[46] Larsen MA, Hartwig JF (2014). Iridium-catalyzed C–H borylation of heteroarenes: scope, regioselectivity, application to late-stage functionalization, and mechanism. J. Am. Chem. Soc..

[47] Preshlock SM (2013). A traceless directing group for C–H borylation. Angew. Chem. Int. Ed..

[48] Murphy JM, Liao X, Hartwig JF (2007). Meta halogenation of 1,3-disubstituted arenes via iridium-catalyzed arene borylation. J. Am. Chem. Soc..

[49] Paul S (2006). Ir-catalyzed functionalization of 2-substituted indoles at the 7-position: nitrogen-directed aromatic borylation. J. Am. Chem. Soc..

[50] Homer JA, Sperry J (2014). A short synthesis of the endogenous plant metabolite 7-hydroxyoxindole-3-acetic acid (7-OH-OxIAA) using simultaneous C–H borylations. Tetrahedron Lett..

[51] Leitch JA, Bhonoah Y, Frost CG (2017). Beyond C2 and C3: transition-metal-catalyzed C–H functionalization of indole. ACS Catal..

[52] Feng Y (2015). Total synthesis of verruculogen and fumitremorgin A enabled by ligand-controlled C–H borylation. J. Am. Chem. Soc..

[53] Ikeda M, Tamura Y (1980). 3-Haloindolenines—versatile intermediates in the indole chemistry. Heterocycles.

[54] Vadola PA, Sames D (2012). Catalytic coupling of arene C–H bonds and alkynes for the synthesis of coumarins: substrate scope and application to the development of neuroimaging agents. J. Org. Chem..

[55] Yang Y, Li R, Zhao Y, Zhao D, Shi Z (2016). Cu-catalyzed direct C6-arylation of indoles. J. Am. Chem. Soc..

[56] Yang G (2014). Pd(II)-catalyzed *meta*-C–H olefination, arylation, and acetoxylation of indolines using a U-shaped template. J. Am. Chem. Soc..

[57] Leitch JA, McMullin CL, Mahon MF, Bhonoah Y, Frost CG (2017). Remote C6-selective ruthenium-catalyzed C–H alkylation of indole derivatives via σ-activation. ACS Catal..

[58] Gribble GW, Johnson JL, Saulnier MG (1981). Stereoselective reduction of 1,2,3,4,6,7,12,12b-octahydroindolo[2,3-*a*]quinolizine with sodium borohydride in trifluoeoacetic acid. Heterocycles.

[59] Okada N, Misawa K, Kitajima M (2007). Preparation of ethylene glycol adducts at 2,3-positions of indoles with hypervalent iodine. Heterocycles.

[60] Saito Y, Segawa Y, Itami K (2015). *para*-C–H borylation of benzene derivatives by a bulky iridium catalyst. J. Am. Chem. Soc..

[61] Litvinas ND, Fier PS, Hartwig JF (2012). A general strategy for the perfluoroalkylation of arenes and arylbromides by using arylboronate esters and [(phen)CuR^F^]. Angew. Chem. Int. Ed..

[62] Eastabrook AS, Wang C, Davison EK, Sperry J (2015). A procedure for transforming indoles into indolequinones. J. Org. Chem..

[63] Meyer-Eppler G (2014). Cheap and easy synthesis of highly functionalized (Het)aryl iodides via the aromatic Finkelstein reaction. Synthesis.

[64] Cooper T, Novak A, Humphreys LD, Walker MD, Woodward S (2006). User-friendly methylation of aryl and vinyl halides and pseudohalides with DABAL-Me_3_. Adv. Synth. Catal..

[65] Wang B, Sun H-X, Sun Z-H (2009). A general and efficient Suzuki-Miyaura cross-coupling protocol using weak base and no water: the essential role of acetate. Eur. J. Org. Chem..

[66] Suresh AS, Baburajan P, Ahmed M (2015). Synthesis of primary amides by aminocarbonylation of aryl/hetero halides using non-gaseous NH_3_ and CO sources. Tetrahedron Lett..

[67] Ramnauth J, Bhardwaj N, Renton P, Rakhit S, Maddaford SP (2003). The room-temperature palladium-catalyzed cyanation of aryl bromides and iodides with tri-*t*-butylphosphine as ligand. Synlett.

[68] Fier PS, Luo J, Hartwig JF (2013). Copper-mediated fluorination of arylboronate esters. identification of a copper(III) fluoride complex. J. Am. Chem. Soc..

[69] Taylor NJ (2017). Derisking the Cu-mediated ^18^F-fluorination of heterocyclic positron emission tomography radioligands. J. Am. Chem. Soc..

[70] Furuya T, Ritter T (2009). Fluorination of boronic acids mediated by silver(I) triflate. Org. Lett..

[71] Tang P, Wang W, Ritter T (2011). Deoxyfluorination of phenols. J. Am. Chem. Soc..

[72] Furuya T, Strom AE, Ritter T (2009). Silver-mediated fluorination of functionalized aryl stannanes. J. Am. Chem. Soc..

[73] Liu S, Scotti JS, Kozmin SA (2013). Emulating the logic of monoterpenoid alkaloid biogenesis to access a skeletally diverse chemical library. J. Org. Chem..

[74] Movassaghi M, Schmidt MA, Ashenhurst JA (2008). Stereoselective oxidative rearrangement of 2-aryl tryptamine derivatives. Org. Lett..

[75] Ishikawa H, Takayama H, Aimi N (2002). Dimerization of indole derivatives with hypervalent iodines(III): a new entry for the concise total synthesis of *rac*- and *meso*-chimonanthines. Tetrahedron Lett..

[76] Jiang LI (2007). Use of a cAMP BRET sensor to characterize a novel regulation of cAMP by the sphingosine 1-phosphate/G_13_ pathway. J. Biol. Chem..

[77] Kenakin T (2017). A scale of agonism and allosteric modulation for assessment of selectivity, bias, and receptor mutation. Mol. Pharm..

[78] Gillis A (2020). Low intrinsic efficacy for G protein activation can explain the improved side effect profiles of new opioid agonists. Sci. Signal..

[79] Stoeber M (2020). Agonist-selective recruitment of engineered protein probes and of GRK2 by opioid receptors in living cells. eLife.

[80] Gutridge AM (2020). G protein-biased kratom-alkaloids and synthetic carfentanil-amide opioids as potential treatments for alcohol use disorder. Br. J. Pharm..

[81] Matsumoto K (2004). Antinociceptive effect of 7-hydroxymitragynine in mice: Discovery of an orally active opioid analgesic from the Thai medicinal herb *Mitragyna speciosa*. Life Sci..

[82] Matsumoto K (2006). Involvement of μ-opioid receptors in antinociception and inhibition of gastrointestinal transit induced by 7-hydroxymitragynine, isolated from Thai herbal medicine *Mitragyna speciosa*. Eur. J. Pharmacol..

[83] Henningfield JE (2019). Risk of death associated with kratom use compared to opioids. Preventive Med..

[84] Schmid CL (2017). Bias factor and therapeutic window correlate to predict safer opioid analgesics. Cell.

[85] Johnson TA (2017). Identification of the first marine-derived opioid receptor “balanced” agonist with a signaling profile that resembles the endorphins. ACS Chem. Neurosci..

[86] Hill R (2018). The novel μ-opioid receptor agonist PZM21 depresses respiration and induces tolerance to antinociception: PZM21 depresses respiration. Br. J. Pharmacol..

[87] Kliewer A (2019). Phosphorylation-deficient G-protein-biased μ-opioid receptors improve analgesia and diminish tolerance but worsen opioid side effects. Nat. Commun..

[88] Kliewer A (2020). Morphine-induced respiratory depression is independent of β‐arrestin2 signalling. Br. J. Pharmacol..

[89] Conibear AE, Kelly E (2019). A biased view of *μ*-opioid receptors?. Mol. Pharm..

[90] Faouzi A (2020). Synthesis and pharmacology of a novel μ–δ opioid receptor heteromer-selective agonist based on the carfentanyl template. J. Med. Chem..

[91] Uprety R (2021). Controlling opioid receptor functional selectivity by targeting distinct subpockets of the orthosteric site. eLife.

[92] Pergolizzi J (2010). Current knowledge of buprenorphine and its unique pharmacological profile. Pain Pract..

[93] Saref A (2019). Self-reported prevalence and severity of opioid and kratom (*Mitragyna speciosa* korth.) side effects. J. Ethnopharmacol..

[94] Garcia-Romeu A, Cox DJ, Smith KE, Dunn KE, Griffiths RR (2020). Kratom (*Mitragyna speciosa*): user demographics, use patterns, and implications for the opioid epidemic. Drug Alcohol Depend..

[95] Kozai TDY, Jaquins-Gerstl AS, Vazquez AL, Michael AC, Cui XT (2015). Brain tissue responses to neural implants impact signal sensitivity and intervention strategies. ACS Chem. Neurosci..

[96] Váradi A (2015). Synthesis of carfentanil amide opioids using the Ugi multicomponent reaction. ACS Chem. Neurosci..

[97] Schuller AGP (1999). Retention of heroin and morphine–6β–glucuronide analgesia in a new line of mice lacking exon 1 of MOR–1. Nat. Neurosci..

[98] Majumdar S (2011). Generation of novel radiolabeled opiates through site-selective iodination. Bioorg. Med. Chem. Lett..

