# Supplementary material for: Site selective C–H functionalization of Mitragyna alkaloids reveals a molecular switch for tuning opioid receptor signaling efficacy
Source: Nat Commun. 2021 Jun 22;12:3858. doi: 10.1038/s41467-021-23736-2 (PMC8219695; doi:10.1038/s41467-021-23736-2)

Supplementary Information

Site Selective C-H Functionalization of *Mitragyna* Alkaloids Reveals a Molecular Switch for Tuning Opioid Receptor Signaling Efficacy

Srijita Bhowmik, Juraj Galeta, Václav Havel, Melissa Nelson, Abdelfattah Faouzi, Benjamin Bechand, Mike Ansonoff, Tomas Fiala, Amanda Hunkele, Andrew C. Kruegel, John. E. Pintar, Susruta Majumdar, Jonathan A. Javitch, Dalibor Sames*

CONTENTS

Pages 2-25: Synthetic Procedures

Pages 25-27: Supplementary Figures 7-10 and Activity of 11-X-7OH analogs at the human and rodent receptors.

Pages 28-31: Biological Procedures

Pages 32-34: References

Pages 34: Acronyms used

Pages 35-63: NMR Spectra

Supplementary Methods

General Considerations (Synthesis) Reagents and solvents were obtained from commercial sources and were used without further purification unless otherwise stated (including anhydrous solvents). Heptane was dried with and kept over activated 3Å MS. All reactions were performed in dried glassware (flame-dried or oven-dried overnight) under an argon atmosphere unless otherwise stated and monitored by TLC on silica-coated plates using solvent mixtures appropriate to each reaction. All column chromatography was performed on silica gel (40-63 μm). For compounds containing a basic nitrogen, Et_3N was often used in the mobile phase in order to provide better resolution. In these cases, TLC plates were pre-soaked in the Et_3N-containing solvent and then allowed to dry briefly before use in analysis, such that an accurate representation of R_f was obtained. Nuclear magnetic resonance spectra were recorded on 400 or 500 MHz instruments as indicated and collected via the Bruker Topspin software (Bruker Topspin 3.5 pl 6). The NMR spectra were analyzed using Mestronova 14.2.0 software. Chemical shifts are reported as δ values in ppm referenced to $CDCl_3$ (^{1}H NMR = 7.26 and ^{13}C NMR = 77.16) and CD_3OD (^{1}H NMR = 3.31 and ^{13}C NMR = 49). Multiplicity is indicated as follows: s (singlet); d (doublet); t (triplet); q (quartet); dd (doublet of doublets); dt (doublet of triplets); td (triplet of doublets); m (multiplet); br (broad). In some cases, spectra are complicated by the presence of multiple conformers, resulting in peak broadening or additional splitting. As a result of these effects, multiple peaks may correspond to the same proton group or carbon atom. When possible, this is indicated by an "and" joining two listed peaks or spectral regions. All carbon peaks are rounded to one decimal place unless such rounding would cause two close peaks to become identical. In these cases, two decimal places are retained. Low resolution mass spectra (LRMS) were recorded on a quadrupole mass spectrometer (ionization mode: APCI+ or ESI+). High-resolution mass spectra (HRMS) were recorded on a quadrupole time-of-flight mass spectrometer (ionization mode: ESI+). Reactions in vials at elevated temperature were performed in an aluminum heating block and the temperature was regulated by a thermometer immersed in a vial containing silicon oil. Mitragynine (MG) was obtained by extraction and purification from commercially available kratom (*Mitragyna speciosa*) leaf powder as previously described.[1,2]

Synthetic procedures:

Optimization of C12-selective borylation reactions of MG and subsequent transformations

Supplementary Figure 1: Known C10/C12 derivatization versus unknown C11 derivatization

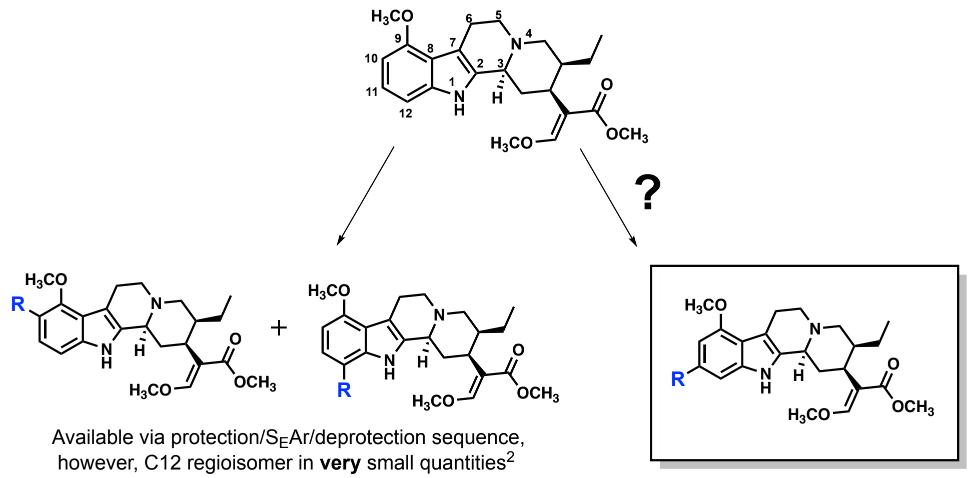

Supplementary Table 1: A comprehensive overview of borylation optimization reactions

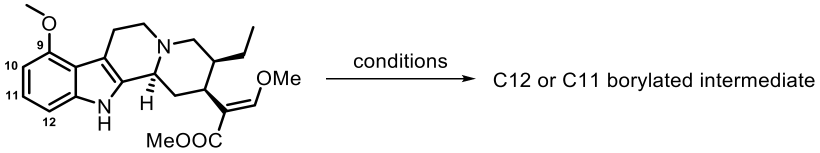

$[Ir(COD)OMe]_2$ (mol%)	ligand (mol%)	Solvent	temperature (°C)	boron source	NMR conv. / reaction time
(not specified)	dtbpy (not specified)	Hexane	60	B_2Pin_2 (1.5 equiv)	no reaction / 24 h
	dtbpy (not specified)	Hexane	60	HBPin (1.5 equiv)	no reaction / 24 h
	dtbpy (not specified)	Heptane	80	B_2Pin_2 (1.5 equiv)	no reaction / 12 h
	dtbpy (not specified)	Heptane	80	HBPin (1.5 equiv)	no reaction / 12 h
	dtbpy (not specified)	1,4-dioxane	80	B_2Pin_2 (1.5 equiv)	no reaction / 12 h
	dtbpy (not specified)	1,4-dioxane	80	HBPin (1.5 equiv)	no reaction / 12 h
	dtbpy (not specified)	THF	80	B_2Pin_2 (1.5 equiv)	no reaction / 12 h
	phenanthroline (not specified)	dry heptane	60	HBPin (2.5 equiv)	no reaction / 24 h
	Me_4-phen (not specified)	dry heptane	60	HBpin (2.5 equiv)	< 20% / 24 h
	dtbpy (not specified)	dry heptane	60	HBPin (2.5 equiv)	< 20% / 24 h
	Me_4-phen (not specified)	dry heptane	80	HBPin (3 equiv)	> 50% / 24 h
	dtbpy (not specified)	dry heptane	80	HBPin (3 equiv)	> 50% / 24 h
	dtbpy (not specified)	dry heptane	80	HBPin (4 equiv)	100% / 24 h
**(5 mol%)**	**dtbpy 10 mol%**	**dry heptane**	**65**	**HBPin (4 equiv)**	**100% C12 / 24 h**

(10 mol%)	Me_4-phen 30 mol%	dry heptane	80	B_2Pin_2 (4 equiv)	100% (C11:C12 3:2) / 24 h
**(10 mol%)**	**dtbpy 30 mol%**	**dry heptane**	**80**	**B_2Pin_2 (4 equiv)**	**100% C12 / 24 h**

Where noted "not specified" for the amount of metal pre-catalyst and ligand in the Table S1, the exploratory experiments were run on 3-5 mg scale of substrate and the smallest amount of pre-catalyst and ligand that can be obtained on a tip of spatula (without weighing) was added. Use of B_2Pin_2 is advantageous since it is a solid easily handled. Thus, in reactions with B_2pin_2, all solids were balanced at once into the reaction vial, flushed with argon and solvent added last (also the case for C11 borylation below). In comparison, reactions with liquid HBPin gave identical results. C12 borylated MG was observed to undergo slow protodeborylation in contact with humidity and air and decomposed rapidly on silica gel (back to starting MG).

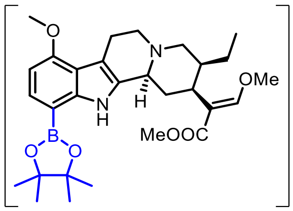

Compound 1: both procedures provide identical results

General Procedure **A**: MG (25 mg, 62 µmol), $[Ir(COD)OMe]_2$ (4 mg, 6.2 µmol), dtbpy (5 mg, 18.8 µmol) and B_2Pin_2 (64 mg, 250 µmol) were balanced into an oven dried vial. The vial was purged with argon, dry heptane (1 mL) was added under argon, and the vial was sealed with a Teflon-lined screw cap and heated to 80 °C. The reaction mixture (RM) became a dark-brown solution after 5–15 min of heating. After 24 h, when LR-MS and TLC (on alumina) indicated complete consumption of starting material (SM), the RM was concentrated to dryness. This crude intermediate was immediately used to prepare the –Cl **2** and –Br **3** derivatives without further purification.

General Procedure **B**: Catalyst $[Ir(COD)OMe]_2$ (4 mg, 6.0 µmol) was dissolved in dry heptane (2.8 mL) in an oven dried vial under argon and HBPin (73 µL, 500 µmol), dtbpy (3.1 mg, 12 µmol) and MG (49 mg, 120 µmol) were added consecutively under a stream of argon, with a 2 min stirring period at RT between each addition. The vial was sealed with a Teflon-lined screw cap and heated to 65 °C. RM was a dark red-brown color. After 24 h, when LR-MS and TLC (on alumina) indicated complete consumption of SM, the RM was concentrated to dryness. This crude intermediate was immediately used to prepare the –Cl **2** and –Br **3** derivatives without further purification.

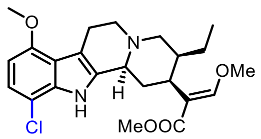

Compound 2 (12-Cl-MG). To the dark residue **1** (from Procedure A) was added $CuCl_2 \cdot H_2O$ (37 mg, 217 μmol) and a mixture of MeOH + H_2O (2 + 0.5 mL). The vial was sealed and the RM was heated to 80 °C with vigorous stirring (slower stirring will cause incomplete conversion due to a precipitation of reactants during the reaction). After 12 h, TLC (EtOAc:Hex 1:1 + 2% Et_3N) and LR-MS indicated complete conversion of the intermediate. Crude reaction mixture was adsorbed on silica and purified by column chromatography using EtOAc:Hex 1:9 + 5% Et_3N. Product **2** was obtained as a yellow, amorphous solid (16 mg, 70% for 2 steps).

^{1}H NMR (400 MHz, $CDCl_3$) δ 7.84 (s, 1H), 7.44 (s, 1H), 6.97 (d, J = 8.3 Hz, 1H), 6.38 (d, J = 8.3 Hz, 1H), 3.86 (s, 3H), 3.75 (s, 3H), 3.72 (s, 3H), 3.16 (dt, J = 11.8, 2.4 Hz, 1H), 3.13 – 2.99 (m, 3H), 2.97 – 2.88 (m, 2H), 2.58 – 2.42 (m, 3H), 1.85 (dt, J = 13.0, 3.1 Hz, 1H), 1.82 – 1.72 (m, 1H), 1.67 – 1.59 (m, 1H), 1.25 – 1.16 (m, 1H), 0.88 (t, J = 7.4 Hz, 3H). **^{13}C NMR (101 MHz, $CDCl_3$)** δ 169.3, 160.7, 153.5, 134.6, 134.0, 120.8, 118.9, 111.6, 109.4, 108.8, 100.7, 77.2, 61.7, 61.4, 57.9, 55.7, 53.7, 51.5, 40.8, 40.0, 30.0, 23.9, 19.3, 13.0. **LR-MS (APCI+)** calcd. for $C_{23}H_{30}ClN_2O_4^+$ $[M+H]^+$: 433.2, found 433.3.

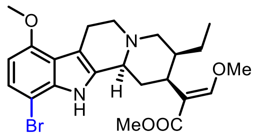

Compound 3 (12-Br-MG). To the dark residue **1** (from Procedure B) was added $CuBr_2$ (41 mg, 186 μmol) and a mixture of MeOH + H_2O (2 + 0.5 mL). The vial was sealed and the RM was heated to 80 °C with vigorous stirring (slower stirring will cause incomplete conversion due to a precipitation of reactants during the reaction). After 12 h, TLC (EtOAc:Hex 1:1 + 2% Et_3N) and LR-MS indicated complete conversion of the intermediate. Crude reaction mixture was adsorbed on silica and purified by column chromatography using EtOAc:Hex 1:9 + 5% Et_3N. Product **3** was obtained as an amorphous, pale-yellow solid (21 mg, 65% for 2 steps).

^{1}H NMR (500 MHz, $CDCl_3$) δ 7.78 (s, 1H), 7.45 (s, 1H), 7.11 (d, J = 8.3 Hz, 1H), 6.36 (d, J = 8.3 Hz, 1H), 3.85 (s, 3H), 3.75 (s, 3H), 3.71 (s, 3H), 3.16 (dd, J = 11.3, 2.5 Hz, 1H), 3.13 – 2.99 (m, 3H), 2.96 – 2.88 (m, 2H), 2.57 – 2.43 (m, 3H), 1.85 (dt, J = 13.0, 3.2 Hz, 1H), 1.82 – 1.72 (m, 1H), 1.63 (dd, J = 8.8, 5.5 Hz, 1H), 1.28 – 1.17 (m, 1H), 0.87 (t, J = 7.4 Hz, 3H). **^{13}C NMR (126 MHz, $CDCl_3$)** δ 169.3, 160.7, 154.1, 135.3, 134.5, 123.7, 118.8, 111.5, 109.6, 101.4, 96.2, 61.8, 61.4, 57.9, 55.7, 53.7, 51.5, 40.9, 40.0, 30.0, 24.0, 19.3, 13.0. **LR-MS (APCI+)** calcd. for $C_{23}H_{30}BrN_2O_4^+$ $[M+H]^+$: 477.2, found 477.4.

Compound 3 (12-Br-MG): Preparation using NBS.

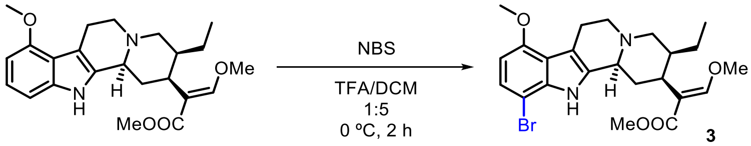

MG (35 mg, 88 μmol) and NBS (15 mg, 88 μmol) were taken in a vial and dissolved in CH_2Cl_2 (1 mL) under argon.[3] The reaction mixture was cooled to 0 °C and TFA (0.20 mL, 11.4 μmol) was added. The reaction was continued for 2 h at 0 °C until LR-MS and TLC indicated complete consumption of SM. The reaction mixture was quenched with saturated aq. $NaHCO_3$ and extracted with EtOAc (3 × 5 mL). Combined EtOAc extracts were dried over Na_2SO_4 and evaporated. The product was purified by PTLC using EtOAc:Hex 1:4 + 2% Et_3N and obtained as an amorphous yellow powder (25 mg, 60%).

Note: Reaction was repeated three times. Out of three repeats, two gave only 12-Br-MG **3** and once we obtained a minor amount of 10-Br-MG (C12 : C10 = 19:5).

The spectral properties of the product were identical to material obtained through the borylation procedure.

Supplementary Figure 2: General strategy for C11 derivatization

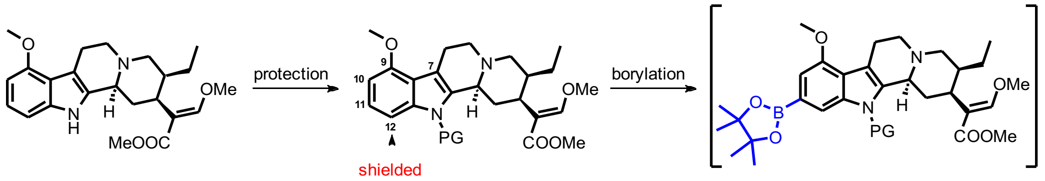

As the iridium-catalyzed borylation reactions are typically sterically controlled, one potential strategy to access the C11 position was to install a PG on the indole nitrogen to shield the C12 position . Accordingly, we attempted the Ir-catalyzed borylation reaction on MG bearing both electron-donating and electron-withdrawing protecting groups.

Supplementary Figure 3: N-protected mitragynine derivatives and subsequent borylation reactions

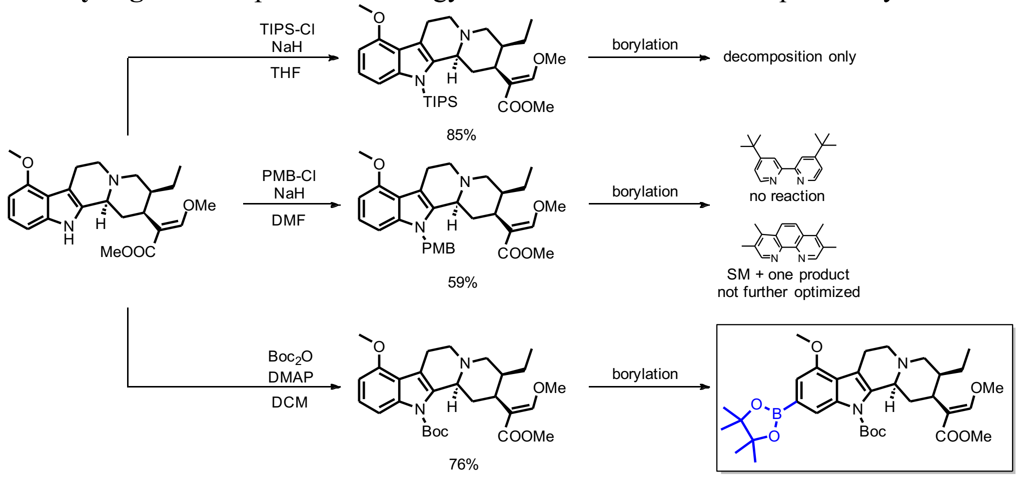

Boc-MG: MG (200 mg, 0.502 mmol) was dissolved in dry DCM (5 mL) and Boc_2O (120 mg, 0.552 mmol) followed by DMAP (6 mg, 0.0502 mmol) were added in one portion. The mixture was stirred for 12 h at RT and directly poured on column for separation (EtOAc:Hex 1:5 + 5% Et_3N; $R_f = 0.48$). The pure product (190 mg, 76%) was obtained as a yellowish, foamy solid.

^1H NMR (400 MHz, $CDCl_3$) δ 7.76 (d, J = 0.7 Hz, 1H), 7.41 (s, 1H), 7.16 (t, J = 8.2 Hz, 1H), 6.65 (dd, J = 8.1, 0.7 Hz, 1H), 3.89 (s, 3H), 3.72 (s, 3H), 3.71 (s, 3H), 3.65 – 3.52 (m, 1H), 3.13 (dt, J = 13.2, 3.5 Hz, 1H), 3.06 (dd, J = 12.0, 2.1 Hz, 2H), 2.96 – 2.84 (m, 2H), 2.76 – 2.61 (m, 2H), 2.33 – 2.16 (m, 1H), 1.98 (dt, J = 13.0, 2.4 Hz, 1H), 1.83 – 1.71 (m, 1H), 1.62 (s, 9H), 1.59 (d, J = 3.9 Hz, 1H), 1.25 – 1.21 (m, 1H), 0.89 (t, J = 7.4 Hz, 3H). ^{13}C NMR (126 MHz, $CDCl_3$) δ 169.6, 160.8, 154.3, 150.9, 139, 135.5, 124.6, 119.2, 116.9, 111.9, 108.6, 103.8, 83.8, 62.9, 61.8, 58.0, 55.7, 51.59, 51.57, 41.0, 40.8, 31.5, 28.5, 25.7, 20.0, 13.6. LR-MS (APCI+) calcd. for $C_{28}H_{39}N_2O_6^+$ $[M+H]^+$: 499.3, found 499.4.

Supplementary Figure 4: *In situ* C11 borylation of **Boc-MG** and subsequent chlorination step

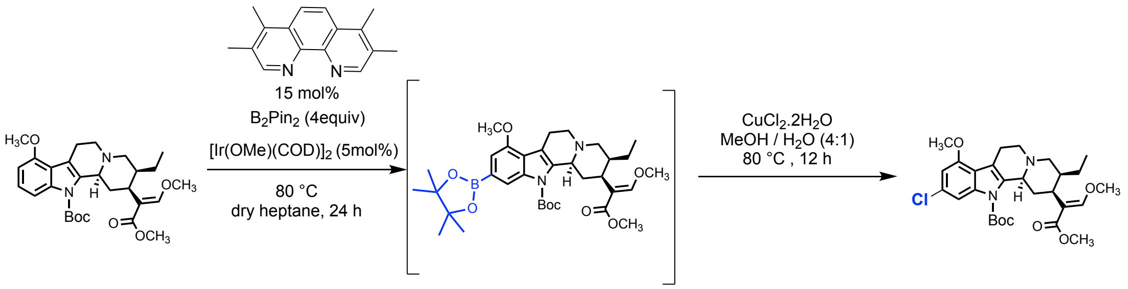

When attempting chlorination of the crude material obtained by borylation of Boc-MG, a mixture of two products, MG and 11-Cl-MG (one spot on TLC) was obtained after column chromatography. We observed significant protodeborylation as well as boc-deprotection under the reaction conditions. Preliminary exploration showed that the halogenation step was strongly concentration and temperature dependent. This process was not further optimized as another protected MG derivative, mitragynine-ethylene glycol adduct MG-EG (**4**), was found to be a superior substrate for C11-borylation compared to Boc-MG.

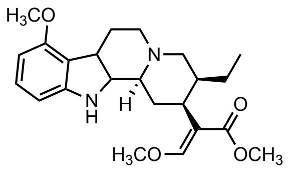

Dihydromitragynine (DHM): MG (100 mg, 251 µmol) was dissolved in neat TFA (1.5 mL). To the solution $NaBH_4$ (37.9 mg, 1.00 mmol) was added and made to dissolve. The vial was left open for some time to let the H_2 gas formed during the reaction to let off and then the reaction was made to continue overnight at room temperature. After the consumption of starting material, the reaction mixture was

quenched with 1M NaOH solution and extracted with CH_2Cl_2 (3 × 25 mL). The combined extract was washed with brine, dried over $MgSO_4$, and evaporated. The product was purified by PTLC using EtOAc:Hex 1:4 + 2% Et_3N. DHM was obtained as a yellow solid (50 mg, 50%).

^{1}H NMR (500 MHz, $CDCl_3$) δ 7.41 (s, 1H), 6.96 (t, *J* = 8.0 Hz, 1H), 6.37 (d, *J* = 7.7 Hz, 1H), 6.29 (dd, *J* = 8.2, 0.6 Hz, 1H), 3.79 (d, *J* = 1.2 Hz, 6H), 3.69 (s, 3H), 3.52 (dd, *J* = 6.6, 3.2 Hz, 1H), 3.05 (dt, *J* = 12.4, 6.4 Hz, 1H), 3.00 – 2.82 (m, 2H), 2.69 (dt, *J* = 11.6, 3.3 Hz, 1H), 2.54 (td, *J* = 13.0, 11.4 Hz, 1H), 2.19 – 2.07 (m, 1H), 2.00 (dt, *J* = 11.6, 2.6 Hz, 1H), 1.96 – 1.75 (m, 4H), 1.62 – 1.48 (m, 2H), 1.44 – 1.34 (m, 1H), 1.29 – 1.11 (m, 1H), 0.84 (t, *J* = 7.4 Hz, 3H). ^{13}C NMR (126 MHz, $CDCl_3$) δ 169.5, 160.7, 156.0, 152.1, 128.4, 121.6, 112.1, 104.8, 102.1, 64.9, 64.7, 61.9, 59.1, 55.5, 55.0, 51.6, 41.1, 40.7, 37.9, 29.6, 27.9, 19.5, 13.4. LR-MS (APCI+) calcd. for $C_{23}H_{33}N_2O_4$ $[M+H]^+$: 401.2, found 401.4.

Supplementary Figure 5: Borylation of MG analogs with oxidized (7OH), reduced (DHM), rearranged indole nucleus (MP) and subsequent bromination step.

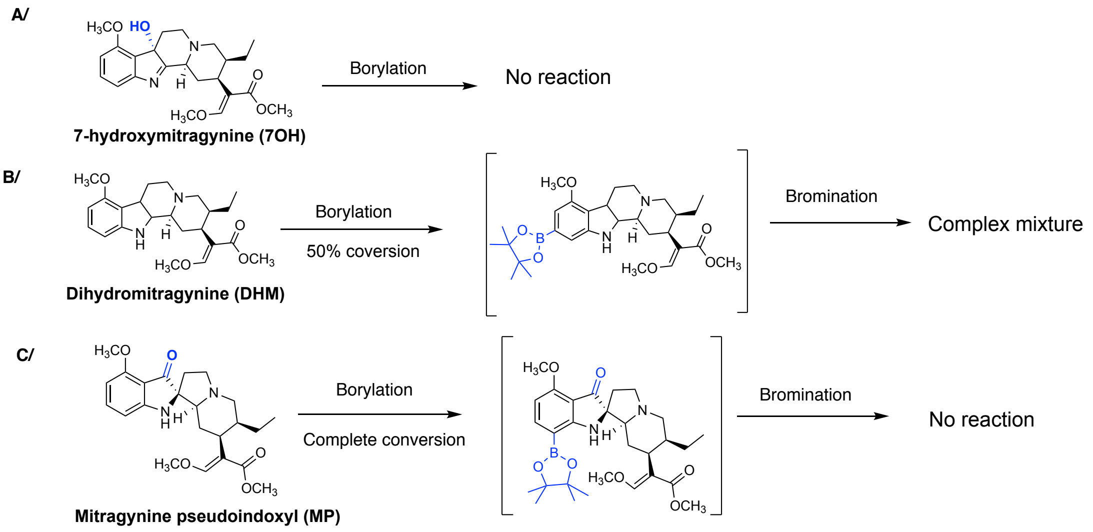

A/ 7OH was synthesized from MG as described in general procedure E.[4] 7OH (25 mg, 60 µmol), $[Ir(COD)OMe]_2$ (5 mol%), ligand (dtbpyl or Me_4-phen) (15 mol%) and B_2Pin_2 (4 equiv) was balanced into an oven dried vial. The vial was purged with argon, dry heptane (1 mL) was added under argon, and the vial was sealed with a Teflon-lined screw cap and heated to 65 °C. The reaction was heated for 24 h and then was concentrated *in vacuo*. No borylation was observed with either ligands as indicated by LR-MS and ^{1}H-NMR.

B/ DHM (25 mg, 62 µmol), $[Ir(COD)OMe]_2$ (5 mol%), ligand (dtbpyl or Me_4-phen) (15 mol%) and B_2Pin_2 (4 equiv) was balanced into an oven dried vial. The vial was purged with argon, dry heptane (1 mL) was added under argon, and the vial was sealed with a Teflon-lined screw cap and heated to 65 °C. The reaction was heated for 24 h and then was concentrated *in vacuo*. ^{1}H-NMR of the crude material showed a partial conversion (50% conversion) to the C11-borylated product along with unreacted SM. To the crude dark residue of intermediate was added $CuBr_2$ (4 equiv) and a mixture of MeOH (2.4 mL) + H_2O (0.6 mL) (4:1). The vial was sealed, and the RM was then heated to 80 °C with vigorous stirring. After 24 h a complex and inseparable mixture of MG and DHM as the major products (~ 60% by ^{1}H NMR), and 11-Br-mitragynine and 11-Br-2,3-dihydromitragynine as the minor products (~ 30% by ^{1}H NMR) was formed.

C/ MP was synthesized according to published procedure.[5] MP (25 mg, 60 µmol), $[Ir(COD)OMe]_2$ (5 mol%), ligand (dtbpyl or Me_4-phen) (15 mol%) and B_2Pin_2 (4 equiv) were balanced into an oven dried vial. The vial was purged with argon, dry heptane (1 mL) was added under argon, and the vial was sealed with a Teflon-lined screw cap and heated to 65 °C. The reaction was heated for 24 h and then was concentrated *in vacuo*. Complete conversion of SM to C12-borylated product was determined through LR-MS and ^{1}H-NMR. To the resulting dark residue of intermediate was added $CuBr_2$ (4 equiv) and a mixture of MeOH (2 mL) + H_2O (0.5 mL) (4:1). The vial was sealed, and the RM was then heated to 80 °C with vigorous stirring for 24 h. No conversion to brominated product was seen.

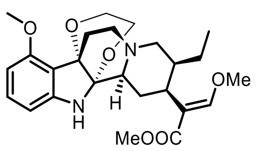

Compound 4 (MG-EG). Reaction was performed according to a published procedure.[2] To a solution of MG (600 mg, 1.51 mmol) in dry MeCN (12 mL) were added dry ethylene glycol (12 mL) and PIFA (649 mg, 1.51 mmol) at 0 °C and the RM was stirred for 1 h at 0 °C under argon atmosphere. After adding chilled saturated aqueous $NaHCO_3$ solution (120 mL), the mixture was extracted with CH_2Cl_2 (3 × 40 mL). The combined extract was washed with brine, dried over $MgSO_4$, and evaporated. The product was purified by column chromatography using 1:9 to 2:8 EtOAc:Hex + 2% Et_3N gradient. Product **4** was obtained as a light green solid (485 mg, 70%) (The green color is due to the residual chlorophyll in the batch of MG extracted from the plant material).

MG-EG crystals were grown by dissolving **4** in hot MeOH and allowing it to slowly cool to room temperature. After the solution was cooled down sufficiently (in less than 1 hour), **4** formed crystals

suitable for an X-ray crystallography. The absolute stereochemistry was assigned based on literature.[6,7] X-ray crystal structure for **4** is deposited in the Cambridge Crystallographic Data Centre CCDC 1905559.

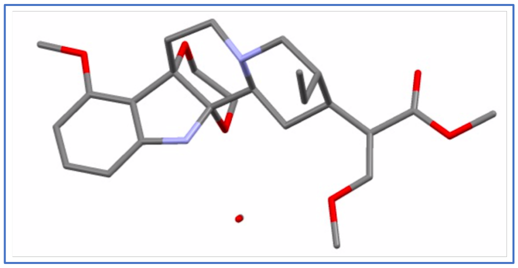

Supplementary Figure 6. ORTEP representation of the X-ray crystal structure of **MG-EG (4)**

^{1}H NMR (400 MHz, $CDCl_3$) δ 7.41 (s, 1H), 7.07 (dd, *J* = 8.3, 7.7 Hz, 1H), 6.43 – 6.26 (m, 2H), 4.25 (s, 1H), 3.92 (td, *J* = 11.6, 2.7 Hz, 1H), 3.86 – 3.81 (m, 4H, signal overlap), 3.78 (s, 3H), 3.70 (s, 3H), 3.67 (dd, *J* = 11.3, 2.3 Hz, 1H), 3.46 – 3.38 (m, 1H), 2.98 (dd, *J* = 11.6, 2.2 Hz, 1H), 2.92 (dt, *J* = 13.0, 3.6 Hz, 1H), 2.53 – 2.42 (m, 2H), 2.39 – 2.30 (m, 2H), 2.28 – 2.23 (m, 1H), 2.15 (dt, *J* = 14.4, 2.5 Hz, 1H), 1.87 – 1.69 (m, 3H), 1.56 (d, *J* = 11.1 Hz, 1H), 1.24 – 1.21 (m, 1H), 0.84 (t, *J* = 7.4 Hz, 3H). ^{13}C NMR (101 MHz, $CDCl_3$) δ 169.2, 160.6, 157.1, 149.1, 130.0, 115.9, 111.9, 105.4, 102.9, 90.9, 81.5, 62.5, 61.8, 61.4, 61.0, 58.7, 55.5, 51.4, 50.4, 40.6, 40.3, 35.6, 24.3, 19.3, 13.2. LR-MS (APCI+) calcd. for $C_{25}H_{35}N_2O_6^+$ $[M+H]^+$: 459.2, found 459.7

Ligand Screening:

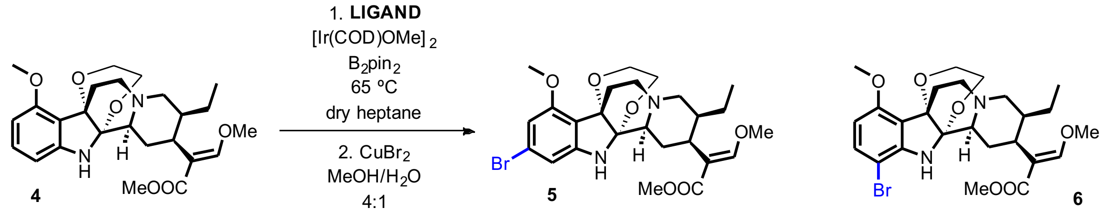

General procedure: Compound **4** (30 mg, 65 µmol), $[Ir(COD)OMe]_2$ (5 mol%), ligand (15 mol%) and B_2Pin_2 (4 equiv) were balanced into an oven dried vial. The vial was purged with argon, dry heptane (1 mL) was added under argon, and the vial was sealed with a Teflon-lined screw cap and heated to 65 °C. The reaction was heated for 15–24 h and then was concentrated *in vacuo*. To the resulting dark residue of intermediate was added $CuBr_2$ (4 equiv) and a mixture of MeOH (2.4 mL) + H_2O (0.6 mL) (4:1). The vial was sealed, and the RM was then heated to 80 °C with vigorous stirring. After 12 h, brine (10 ml) was added and the mixture was extracted with DCM (3 × 5 mL). The combined DCM extracts were dried over Na_2SO_4 and evaporated. The product was purified by PTLC using EtOAc:Hex 1:4 + 2% Et_3N (the plate

was developed twice to improve resolution). After PTLC, the product was obtained as an inseparable mixture of **5** and **6** and the ratio was analyzed by ^{1}H-NMR (see Supplementary Table 2)

Supplementary Table 2. Ligand screening of MG-EG (4) borylation/bromination for product distribution

Entry	Ligand	Ratio (**5**:**6**)
1.	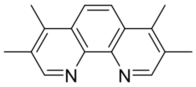	**> 16:1**
2.	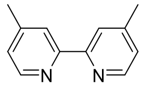	1:1.25
3.	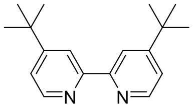	≈ 1:1
4.	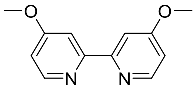	2.8:1
5.	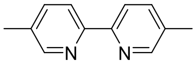	negligible borylation observed
6.	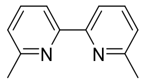	no borylation

C-H borylation reaction (selectivity C11:C12 = 1.3-1.1:1) of 4 and subsequent bromination [Entry 3, Supporting Table 2]

Starting material **4** (25 mg, 55 μmol), $[Ir(COD)OMe]_2$ (1.8 mg, 2.7 μmol), dtbpy (2.2 mg, 8.1 μmol) and B_2Pin_2 (55 mg, 0.22 mmol) were balanced into an oven dried vial. The vial was purged with argon, dry heptane (1 mL) was added under argon, and the vial was sealed with a Teflon lined screw cap and heated to 65 °C. The RM became a dark brown solution after 5–15 minutes of heating. After 15–24 h, when LR-MS indicated complete consumption of SM, the RM was concentrated to dryness. This intermediate was immediately used for further transformation.

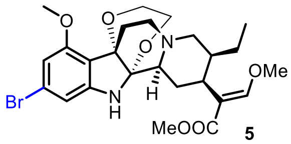

5

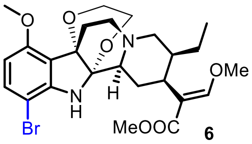

6

To the dark residue of borylated intermediate was added $CuBr_2$ (37 mg, 0.17 mmol) and a mixture of MeOH + H_2O (2 + 0.5 mL). The vial was sealed, and the RM was then heated to 80 °C with vigorous stirring

(slower stirring will cause incomplete conversion due to a precipitation of reactants during the reaction). After 15 h, TLC (EtOAc:Hex 1:1 + 2% Et_3N) and LR-MS indicated complete conversion of the intermediate. The RM was diluted with brine (15 mL) and extracted with DCM (3 × 5 mL). The combined DCM extracts were dried over Na_2SO_4 and evaporated. The product was purified by PTLC using EtOAc:Hex 1:4 + 2% Et_3N (the plate was developed twice to improve resolution). Products **5** and **6** were obtained as a mixture (C11:C12 - 1.3-1.1:1) in the form of a pale-yellow solid (20-24 mg, 60-83%).

C-H borylation reaction (selectivity C11:C12 = 16-20:1) of 4 and subsequent transformations

General procedure C: Compound **4** (50 mg, 0.11 mmol), $[Ir(COD)OMe]_2$ (3.6 mg, 5.5 µmol), Me_4-phen (3.9 mg, 16 µmol) and B_2Pin_2 (111 mg, 0.44 mmol) were balanced into an oven dried vial. The vial was purged with argon, dry heptane (2.5 mL) was added under argon, and the vial was sealed with a Teflon-lined screw cap and heated to 65 °C. The RM became a dark red-brown solution after 5–15 minutes of heating. After 17–24 h, when LR-MS indicated complete consumption of SM, the RM was concentrated to give the crude boronate ester. This intermediate was immediately used to prepare the -Cl (**13**), -Br (**5**), and -OH (**14**) derivatives without further purification.

Up to 100 mg of **4** can be used in one reaction vial (8 mL volume) to prepare the boronate ester intermediate using only 1.5 mL of dry heptane. It is however necessary to divide the crude intermediate into multiple reaction vessels for the subsequent reaction (the reactions are very sensitive to the ratio of MeOH/H_2O and total reaction volume). The intermediate boronate ester is air and moisture sensitive and unstable during a silica gel column purification. Other solvents than those used for further reactions (mainly chlorinated, e.g. DCM and $CHCl_3$) can lead to a significant protodeborylation. Compounds prepared from the boronate ester intermediate are sometimes contaminated with decomposition products from excess of B_2Pin_2 (like pinacol). These can be removed either by repeated chromatography purification (preferably PTLC) or after further reaction steps.

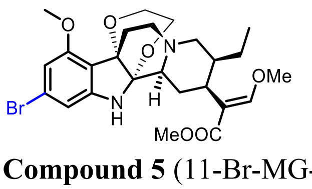

Compound 5 (11-Br-MG-EG). The boronate ester intermediate was prepared from **4** (50 mg, 0.11 mmol) as described in the general procedure C. To the dark residue of borylated intermediate was added $CuBr_2$ (73 mg, 0.33 mmol) and a mixture of MeOH + H_2O (4 + 1 mL). The vial was sealed and the RM was then heated to 80 °C with vigorous stirring (slower stirring will cause incomplete conversion due to precipitation of reactants during the reaction). After 15 h, TLC (EtOAc:Hex 1:1 + 2% Et_3N) and LR-MS indicated complete conversion of the intermediate. The RM was diluted with brine (15 mL), extracted with DCM (3

× 10 mL), and the combined DCM extracts were dried over Na_2SO_4 and evaporated. The product was purified by PTLC using EtOAc:Hex 1:4 + 2% Et_3N (the plate was developed twice to improve resolution). Product **5** (along with 3-5% of inseparable C12 isomer**)** was obtained as a pale-yellow solid (35 mg, 60%). ^{1}H NMR (400 MHz, $CDCl_3$) δ 7.40 (s, 1H), 6.54 (s, 2H), 4.29 (s, 1H), 3.91 (td, *J* = 11.6, 2.8 Hz, 1H), 3.82 (s, 3H), 3.82 – 3.74 (m, 4H), 3.70 (s, 3H), 3.66 (dd, *J* = 11.6, 2.7 Hz, 1H), 3.41 (dd, *J* = 11.6, 2.5 Hz, 1H), 2.98 (dd, *J* = 11.5, 2.2 Hz, 1H), 2.91 (dt, *J* = 13.0, 3.5 Hz, 1H), 2.49 – 2.40 (m, 2H), 2.38 – 2.29 (m, 2H), 2.29 – 2.22 (m, 1H), 2.13 (dd, *J* = 14.4, 2.5 Hz, 1H), 1.83 – 1.69 (m, 3H), 1.56 (d, *J* = 15.0 Hz, 1H), 1.24 – 1.21 (m, 1H), 0.84 (t, *J* = 7.4 Hz, 3H). ^{13}C NMR (126 MHz, $CDCl_3$) δ 169.2, 160.5, 157.3, 150.0, 122.8, 115.0, 111.7, 108.7, 106.7, 91.0, 81.1, 62.5, 61.8, 61.3, 60.9, 58.6, 55.7, 51.4, 50.2, 40.6, 40.2, 35.4, 24.2, 19.3, 13.2. HRMS (ESI+) calcd. for $C_{25}H_{34}BrN_2O_6^+$ $[M+H]^+$: 539.1584, found 539.1592

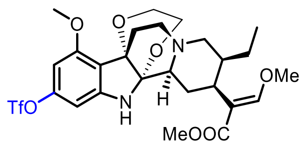

Compound 14a (11-OTf-MG-EG). To a solution of **14** (115 mg, 0.24 mmol) in dry DMF (3 mL) at RT was added DIPEA (2 mL, 1.2 mmol) and *N*-phenyl-bis(trifluoromethanesulfonimide) (173 mg, 0.48 mmol). After the RM was stirred at 50 °C overnight, it was quenched by the addition of saturated aqueous $NaHCO_3$ and extracted with DCM (3 × 15 mL). The combined organic phases were washed with brine, dried over Na_2SO_4 and evaporated. The product was purified by column chromatography using EtOAc:Hex 3:7 + 2% Et_3N. Product **14a** was obtained as a yellow powder (101 mg, 70%).

^{1}H NMR (400 MHz, $CDCl_3$) δ 7.41 (s, 1H), 6.28 (d, *J* = 3.8 Hz, 2H), 4.46 (s, 1H), 3.92 (td, *J* = 11.7, 3.1 Hz, 1H), 3.84 (s, 3H), 3.82 – 3.74 (m, 4H), 3.73 – 3.65 (m, 4H), 3.43 (dd, *J* = 11.7, 2.5 Hz, 1H), 2.99 (dd, *J* = 11.5, 2.1 Hz, 1H), 2.92 (dt, *J* = 13.1, 3.6 Hz, 1H), 2.51 – 2.40 (m, 2H), 2.38 – 2.22 (m, 3H), 2.14 (dt, *J* = 14.4, 2.5 Hz, 1H), 1.81 – 1.74 (m, 2H), 1.74 – 1.64 (m, 1H), 1.57 (d, *J* = 11.2 Hz, 1H), 1.22 – 1.15 (m, 1H), 0.85 (t, *J* = 7.4 Hz, 3H). ^{19}F NMR (471 MHz, $CDCl_3$) δ -72.03. ^{13}C NMR (126 MHz, $CDCl_3$) δ 169.2, 160.6, 157.3, 150.8, 149.9, 122.8, 120.1, 117.5, 115.8, 115.0, 111.6, 98.6, 96.8, 91.1, 81.0, 62.6, 61.8, 61.3, 60.8, 58.6, 55.9, 51.4, 50.2, 40.5, 40.1, 35.4, 24.3, 19.3, 13.2. LR-MS (APCI+) calcd. for $C_{26}H_{34}F_3N_2O_9S^+$ $[M+H]^+$: 607.2, found 607.9.

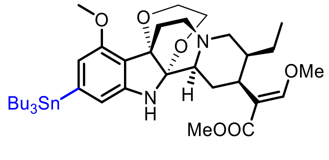

Compound 14b (11-Bu$_3$Sn-MG-EG)

Synthesis from Bromide 5

To a solution of **5** (75 mg, 0.14 mmol) in dry dioxane (0.7 mL) at RT was added lithium chloride (30 mg, 0.70 mmol), $Pd(PPh_3)_4$ (24.3 mg, 0.021 mmol, 10 mol%) and bis(tributyltin) (350 μL, 0.70 mmol). After stirring for 24 h at 100 °C, the RM was cooled to RT and concentrated *in vacuo*. The residue was dissolved in hexanes and then filtered through a pad of celite. After washing the filter cake several times with hexanes, the combined washings were evaporated. The product was purified by column chromatography using Hex + 5% Et_3N. The intermediate **14b** was obtained as a yellow liquid (63 mg, 60%).

Synthesis from Triflate 14a: To a solution of **14a** (85 mg, 0.14 mmol) in dry dioxane (0.7 mL) at RT was added lithium chloride (30 mg, 0.70 mmol), $Pd(PPh_3)_4$ (24.3 mg, 0.021 mmol, 10 mol%) and bis(tributyltin) (350 μL, 0.70 mmol). After stirring for 24 h at 100 °C, the reaction mixture was cooled to RT and concentrated *in vacuo*. The residue was dissolved in hexanes and then filtered through a pad of celite. After washing the filter cake several times with hexanes, the combined washings were evaporated. The product was purified by column chromatography using Hex + 5% Et_3N. The arylstannane intermediate **14b** was obtained as a yellow liquid (74 mg, 70%).
^{1}H NMR (500 MHz, $CDCl_3$) δ 7.44 (s, 1H), 6.80 – 5.74 (m, 2H), 4.23 (s, 1H), 3.95 (td, *J* = 11.6, 2.5 Hz, 1H), 3.87 (s, 4H), 3.81 (s, 3H), 3.72 (s, 3H), 3.69 (dd, *J* = 11.4, 2.3 Hz, 1H), 3.45 (dd, *J* = 11.4, 2.3 Hz, 1H), 3.01 (dd, *J* = 11.6, 2.1 Hz, 1H), 2.94 (dt, *J* = 13.1, 3.6 Hz, 1H), 2.54 – 2.44 (m, 2H), 2.43 – 2.33 (m, 2H), 2.28 (dd, *J* = 11.3, 3.3 Hz, 1H), 2.16 (dt, *J* = 14.4, 2.4 Hz, 1H), 1.91 – 1.74 (m, 3H), 1.61 – 1.48 (m, 6H), 1.35 (h, *J* = 7.3 Hz, 6H), 1.25 – 1.19 (m, 1H), 1.14 – 0.99 (m, 7H), 0.92 (t, *J* = 7.3 Hz, 9H), 0.86 (t, *J* = 7.4 Hz, 3H). ^{13}C NMR (126 MHz, $CDCl_3$) δ 169.2, 160.4, 156.3, 148.4, 143.9, 115.9, 113.0, 111.7, 109.9, 90.8, 81.5, 62.4, 61.7, 61.27, 60.9, 58.5, 55.2, 51.3, 50.3, 40.5, 40.2, 35.5, 29.1, 27.4, 24.2, 19.2, 13.7, 13.1, 9.7. LR-MS (APCI+) calcd. for $C_{37}H_{61}N_2O_6Sn^+$ $[M+H]^+$: 749.4, found 749.6.

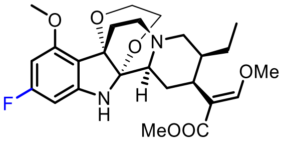

Compound 7 (11-F-MG-EG). To a solution of **14b** (81 mg, 0.11 mmol) in dry acetone (2.0 mL) at RT was added silver triflate (56 mg, 0.22 mmol) and freshly prepared 1-chloromethyl-4-fluoro-1,4-diazoniabicyclo[2.2.2]octane bis(hexafluorophosphate[8] (54 mg, 0.165 mmol). The RM was stirred for 20 min at RT and then concentrated *in vacuo*. Residue was dissolved in DCM and a few drops of 1N HCl solution was added to the solution mixture and then extracted with DCM, dried over Na_2SO_4 and the purified by PTLC. MG-EG (~ 20 –25% formed by destannylation) is usually formed along with the desired

product, which can be purified by PTLC using EtOAc:Hex 1:4 + 2% Et_3N by developing the plate multiple times. Product **7** was obtained as a white solid (31 mg, 60%).

^{1}H NMR (400 MHz, $CDCl_3$) δ 7.40 (s, 1H), 6.39 – 5.84 (m, 2H), 4.31 (s, 1H), 3.91 (td, *J* = 11.7, 2.7 Hz, 1H), 3.84 – 3.75 (m, 7H), 3.70 (s, 3H), 3.66 (dd, *J* = 11.5, 2.5 Hz, 1H), 3.42 (dd, *J* = 11.6, 2.4 Hz, 1H), 3.02 – 2.93 (m, 1H), 2.91 (dt, *J* = 13.0, 3.6 Hz, 1H), 2.44 (d, *J* = 11.6 Hz, 2H), 2.40 – 2.19 (m, 3H), 2.19 – 2.05 (m, 1H), 1.90 – 1.64 (m, 3H), 1.58 (s, 1H), 1.26 (s, 1H), 0.84 (t, *J* = 7.4 Hz, 3H). ^{13}C NMR (fluorine decoupled) (126 MHz, $CDCl_3$) δ 169.2, 164.8, 160.6, 157.6, 149.8, 111.8, 111.3, 93.0, 91.2, 91.2, 80.9, 62.4, 61.8, 61.4, 60.9, 58.7, 55.7, 51.4, 50.4, 40.6, 40.2, 35.7, 24.3, 19.3, 13.2. ^{19}F (proton decoupled) NMR (376 MHz, $CDCl_3$) δ -110.19. HRMS (ESI+) calcd. for $C_{25}H_{34}FN_2O_6^+$ $[M+H]^+$: 477.2401, found 477.2405.

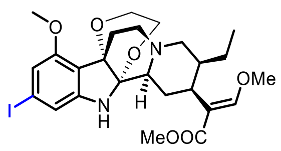

Compound 8 (11-I-MG-EG). Compound **5** (20.0 mg, 37.2 µmol), dried NaI (15.6 mg, 0.1 mmol), CuI (0.5 mg, 2.6 µmol) and *N,N'*-dimethylethylenediamine (2.3 mg, 26 µmol) were balanced into an oven-dried vial. The vial was purged with argon and dry 1,4-dioxane (0.25 mL) was added under a stream of argon. The RM was heated to 110 °C for 24 h. LR-MS indicated formation of product but with a low conversion (TLC is not indicative enough due to a low difference in Rf value of starting compound and product). The RM was evaporated and dried under high vacuum. More NaI (63.7 mg, 0.42 mmol), CuI (3 mg, 16 µmol) and *N*,*N'*-dimethylethylenediamine (9.4 mg, 11 µmol) were added followed by dry 1,4-dioxane (0.3 mL) under Ar. The RM was heated for 22 h, after which LR-MS indicated nearly total conversion. The RM was diluted with DCM (10 mL) and washed 3× with diluted aq. NH_3 (H_2O:28% aq. NH_3 10:0.1 mL), DCM was dried over Na_2SO_4 and evaporated. The crude product was filtered through silica gel in EtOAc:Hex 1:1 + 2% Et_3N and further purified by PTLC (EtOAc:Hex 1:4 + 2% Et_3N, 2× developed). Product **8** was obtained as a yellow solid (13 mg, 59%).

^{1}H NMR (500 MHz, $CDCl_3$) δ 7.40 (s, 1H), 6.72 (d, *J* = 10.7 Hz, 2H), 4.28 (s, 1H), 3.91 (td, *J* = 11.8, 2.9 Hz, 1H), 3.85 – 3.80 (m, 3H), 3.80 – 3.74 (m, 4H), 3.72 – 3.63 (m, 4H), 3.47 – 3.37 (m, 1H), 3.03 – 2.94 (m, 1H), 2.90 (dt, *J* = 13.2, 3.6 Hz, 1H), 2.51 – 2.39 (m, 2H), 2.38 – 2.29 (m, 2H), 2.28 – 2.21 (m, 1H), 2.11 (dt, *J* = 14.5, 2.6 Hz, 1H), 1.84 – 1.66 (m, 3H), 1.55 (d, *J* = 11.1 Hz, 1H), 1.24 – 1.16 (m, 1H), 0.84 (t, *J* = 7.2 Hz, 3H). ^{13}C NMR (126 MHz, $CDCl_3$) δ 169.2, 160.6, 157.4, 150.2, 116.0, 114.7, 112.7, 111.7, 94.0, 90.9, 81.3, 62.5, 61.8, 61.3, 60.8, 58.6, 55.8, 51.4, 50.2, 40.6, 40.2, 35.4, 24.3, 19.3, 13.2. LR-MS (APCI+) calcd. for $C_{25}H_{34}IN_2O_6^+$ $[M+H]^+$: 585.1, found 585.2.

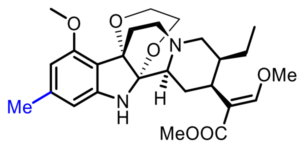

Compound 9 (11-CH_3-MG-EG). Compound **5** (30 mg, 56 µmol), $Pd_2(dba)_3$ (6 mg, 6.7 µmol), Xphos (4.4 mg, 9.2 µmol), and bis(trimethylaluminum)-1,4-diazabicyclo[2.2.2]octane adduct (DABAL-Me_3, 57 mg, 0.22 mmol) were balanced into an oven dried vial. The vial was purged with argon, dry THF (1.5 mL) was added under argon, and the vial was sealed with a Teflon-lined screw cap and heated to 60 °C. After stirring for 2 h, complete conversion was observed by TLC-MS. The reaction mixture was cooled to RT and concentrated *in vacuo*. The product was purified by column chromatography using EtOAc:Hex 1:4 + 2% Et_3N. Product **9** was obtained as a yellow solid (16 mg, 60%).

^{1}H NMR (500 MHz, $CDCl_3$) δ 7.41 (s, 1H), 6.21 (d, *J* = 0.8 Hz, 2H), 4.17 (s, 1H), 3.91 (td, *J* = 11.6, 2.6 Hz, 1H), 3.86 – 3.82 (m, 1H), 3.82 (s, 3H), 3.78 (s, 3H), 3.69 (s, 3H), 3.65 (dd, *J* = 11.4, 2.4 Hz, 1H), 3.47 – 3.33 (m, 1H), 2.98 (dd, *J* = 11.5, 2.1 Hz, 1H), 2.91 (dt, *J* = 13.1, 3.6 Hz, 1H), 2.45 (tt, *J* = 9.4, 2.9 Hz, 2H), 2.40 – 2.28 (m, 2H), 2.27 (s, 3H), 2.24 – 2.19 (m, 1H), 2.13 (dt, *J* = 14.9, 2.8 Hz, 1H), 1.85 – 1.71 (m, 3H), 1.55 (d, *J* = 11.3 Hz, 1H), 1.25 – 1.16 (m, 1H), 0.83 (t, *J* = 7.4 Hz, 3H). ^{13}C NMR (126 MHz, $CDCl_3$) δ 169.2, 160.4, 156.7, 149.1, 140.3, 113.0, 111.7, 106.0, 103.8, 90.9, 81.2, 62.3, 61.6, 61.3, 60.9, 58.5, 55.2, 51.3, 50.3, 40.5, 40.1, 35.6, 24.2, 22.1, 19.2, 13.1. LR-MS (APCI+) calcd. for $C_{26}H_{37}N_2O_6^+$ $[M+H]^+$: 473.3, found 473.8.

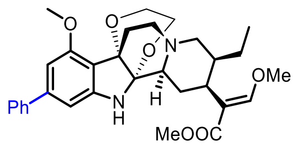

Compound 10 (11-Ph-MG-EG). Compound **5** (20 mg, 37 µmol), phenylboronic acid (9.8 mg, 80 µmol), CsOAc (16.1 mg, 84 µmol, thoroughly dried under high vacuum while heating until melted) and $Pd(dppf)Cl_2·CH_2Cl_2$ (2.0 mg, 2.4 µmol) were balanced into an oven-dried vial. The vial was purged with argon, dry THF (0.3 mL) was added under a stream of argon, and the vial was closed with a Teflon-lined solid screw cap and heated to 70 °C. After 7 h, LR-MS and TLC indicated full consumption of starting material. The RM was diluted with brine (5 mL) and extracted with DCM (3 × 5 mL). The combined DCM extracts were dried over Na_2SO_4, evaporated, and the crude residue was purified by PTLC (EtOAc:Hex 1:4 + 2% Et_3N and Et_2O + 1% Et_3N). Product 10 was obtained as a pale-yellow solid (16 mg, 81%).

^{1}H NMR (400 MHz, $CDCl_3$) δ 7.57 – 7.50 (m, 2H), 7.44 – 7.37 (m, 3H), 7.35 – 7.29 (m, 1H), 6.62 – 6.56 (m, 2H), 4.33 (s, 1H), 3.98 – 3.81 (m, 5H), 3.79 (s, 3H), 3.70 (s, 4H), 3.49 – 3.42 (m, 1H), 3.00 (dd, *J* = 11.5, 2.1 Hz, 1H), 2.93 (dt, *J* = 13.0, 3.6 Hz, 1H), 2.56 – 2.43 (m, 2H), 2.43 – 2.32 (m, 2H), 2.32 – 2.23 (m, 1H), 2.19 (dt, *J* = 14.3, 2.5 Hz, 1H), 1.92 – 1.69 (m, 3H), 1.58 (d, *J* = 11.1 Hz, 1H), 1.28 – 1.21 (m, 1H),

0.85 (t, J = 7.4 Hz, 3H). ^{13}C NMR (126 MHz, $CDCl_3$) δ 169.3, 160.6, 157.1, 149.5, 143.9, 142.0, 128.7, 127.3, 115.1, 111.8, 104.5, 102.6, 91.1, 81.4, 62.6, 61.8, 61.4, 61.0, 58.6, 55.5, 51.4, 50.4, 40.6, 40.3, 35.7, 24.3, 19.3, 13.2. Overlap between carbon signals for phenyl CH and acrylic CH. LR-MS (APCI+) calcd. for $C_{31}H_{39}N_2O_6^+$ $[M+H]^+$: 535.3, found 536.0.

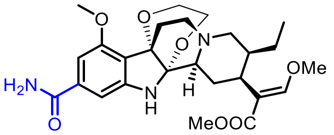

Compound 11 (11-CONH$_2$-MG-EG)

Synthesis from Bromide 5. Compound **5** (20.0 mg, 37 µmol), $Pd(OAc)_2$ (0.5 mg, 2.2 µmol), dppf (2.6 mg, 4.7 µmol), imidazole (2.6 mg, 38 µmol), $Co_2(CO)_8$ (7.6 mg, 22 µmol) and NH_4Cl (8.0 mg, 0.15 mmol) were balanced into an oven-dried vial. The vial was purged with argon and dry 1,4-dioxane (0.8 mL) was added followed by DIPEA (26 µL, 0.15 mmol) under a stream of argon. The vial was sealed with a Teflon-lined screw cap and the RM was heated to 90 °C. After 15 h, TLC (EtOAc:Hex 1:1 + 2% Et_3N) indicated only partial conversion, so additional $Co_2(CO)_8$ (8.0 mg, 23 µmol), NH_4Cl (8.0 mg, 0.15 mmol) and DIPEA (26 µL, 0.15 mmol) were added. Heating was continued for another 24 h (no further conversion was observed). The RM was diluted with MeOH and adsorbed on celite. The compound was purified by column chromatography with a gradient of EtOAc:Hex 1:1 + 2% Et_3N with 5 to 10% MeOH. The product was further purified on PTLC in acetone:Hex 1:1 + 2% Et_3N and coeluting Et_3N salts were removed by washing of its $CHCl_3$ solution with 2M Na_2CO_3. Product **11** was obtained as a pale-yellow solid (6.5 mg, 35%).

Synthesis from Triflate 14a. Compound **14a** (25 mg, 41 µmol), $Pd(OAc)_2$ (0.8 mg, 3.6 µmol), dppf (2.7 mg, 4.9 µmol), imidazole (0.9 mg, 13 µmol), $Co_2(CO)_8$ (9.7 mg, 28 µmol) and NH_4Cl (9.3 mg, 0.17 mmol) were balanced into an oven-dried vial. The vial was purged with argon and dry 1,4-dioxane (0.9 mL) was added followed by DIPEA (30 µL, 0.17 mmol) under a stream of argon. The vial was sealed with a Teflon-lined screw cap and the RM was heated to 90 °C. After 21.5 h, TLC (EtOAc:Hex 1:1 + 2% Et_3N) indicated full conversion, so the RM was diluted with MeOH and adsorbed on celite. The product was partially separated by column chromatography in EtOAc + 2% Et_3N to acetone + 2% Et_3N (compound spread and did not separate very well). Product was further purified on PTLC in EtOAc:acetone 2:1 + 2% Et_3N (developed 2× to improve resolution). Finally, the compound was eluted from SiO_2 with acetone (no Et_3N) to remove Et_3N salts. Product **11** was obtained as a pale-yellow solid (11.7 mg, 57%).

^{1}H NMR (500 MHz, $CDCl_3$) δ 7.39 (s, 1H), 6.93 (s, 1H), 6.65 (s, 1H), 5.97 (s, 1H), 5.73 (s, 1H), 4.44 (s, 1H), 3.98 – 3.84 (m, 4H), 3.79 (s, 4H), 3.70 (s, 4H), 3.43 (dd, J = 11.8, 2.5 Hz, 1H), 3.05 – 2.95 (m, 1H), 2.91 (dt, J = 13.1, 3.6 Hz, 1H), 2.47 (t, J = 9.9 Hz, 2H), 2.35 (td, J = 12.0, 5.3 Hz, 2H), 2.26 (dd, J = 11.9, 3.3 Hz, 1H), 2.21 – 2.11 (m, 1H), 1.87 – 1.64 (m, 3H), 1.56 (d, J = 11.2 Hz, 1H), 1.26 – 1.18 (m, 1H), 0.84

(t, J = 7.3 Hz, 3H). ^{13}C NMR (126 MHz, $CDCl_3$) δ 169.7, 169.2, 160.6, 157.2, 149.3, 135.7, 120.1, 111.8, 103.7, 103.3, 91.2, 81.3, 62.7, 61.8, 61.4, 60.8, 58.6, 55.8, 51.4, 50.2, 40.6, 40.2, 35.3, 24.3, 19.3, 13.2 LR-MS (APCI+) calcd. for $C_{26}H_{36}N_3O_7^+$ $[M+H]^+$: 502.3, found 502.6.

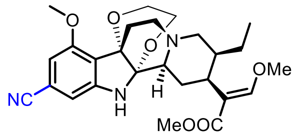

Compound 12 (11-CN-MG-EG). Compound **5** (30 mg, 0.05 mmol), $Pd_2(dba)_3$ (30.5 mg, 0.033 mmol), Zn dust (5.4 mg, 0.08 mmol), $Zn(CN)_2$ (11.5 mg, 0.09 mmol) and $[HP(^tBu)_3]BF_4$ (19.1 mg, 0.06 mmol) were balanced into an oven-dried vial. The vial was purged with argon, dry DMF (1.6 mL) was added under a stream of argon, and the vial was closed with a Teflon-lined solid screw cap and reacted at RT for 1 h. After 1 h, LR-MS and TLC indicated full consumption of starting material. The RM was diluted with brine (5 mL) and extracted with EtOAc (3 × 5 mL). The combined EtOAc extracts were dried over Na_2SO_4, evaporated, and the crude residue was purified by PTLC (EtOAc:Hex 1:4 + 2% Et_3N and Et_2O + 1% Et_3N). The product could not be purified as the free base and so was converted to its HCl salt. After the HCl salt was formed it was washed with hexane for several times to purify it. Product **12** was then obtained as the HCl salt as a pale-yellow solid (14 mg, 60%).

^1H NMR (400 MHz, CD_3OD) (HCl salt of 12) δ 7.63 (s, 1H), 6.92 (d, *J* = 1.1 Hz, 1H), 6.79 (d, *J* = 1.1 Hz, 1H), 3.98 – 3.94 (m, 1H), 3.92 (s, 3H), 3.90 (s, 3H), 3.79 – 3.76 (m, 2H), 3.73 (s, 3H), 3.64 – 3.49 (m, 2H), 3.46 – 3.37 (m, 2H), 3.37 (s, 1H), 3.31 – 3.20 (m, 2H), 2.65 (dt, *J* = 14.8, 12.8 Hz, 1H), 2.41 (dt, *J* = 15.4, 2.5 Hz, 1H), 2.14 (d, *J* = 14.8 Hz, 1H), 2.09 – 1.92 (m, 2H), 1.69 – 1.45 (m, 2H), 1.34 (t, *J* = 7.4 Hz, 1H), 0.97 (t, *J* = 7.3 Hz, 3H). ^{13}C NMR (101 MHz, CD_3OD) δ 168.5, 161.9, 157.2, 149.6, 119.9, 118.5, 113.7, 108.6, 108.5, 107.3, 88.8, 80.0, 62.4, 61.2, 60.6, 60.2, 56.1, 55.0, 50.5, 49.9, 39.1, 37.3, 32.2, 22.4, 17.7, 11.3. LR-MS (APCI+) calcd. for $C_{26}H_{34}N_3O_6^+$ $[M+H]^+$: 484.3, found 484.6

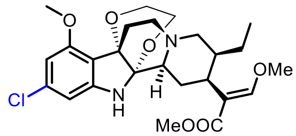

Compound 13 (11-Cl-MG-EG). The boronate ester intermediate was prepared from **4** (50 mg, 0.11 mmol) as described in the general procedure C. To the dark residue of intermediate was added $CuCl_2·2H_2O$ (53.0 mg, 0.31 mmol) and a mixture of MeOH + H_2O (4 + 1 mL). The vial was sealed, and the RM was then heated to 80 °C with vigorous stirring (slower stirring will cause incomplete conversion due to precipitation during the reaction). After 17 h, TLC (EtOAc:Hex 1:1 + 2% Et_3N) and LR-MS indicated complete conversion of the intermediate. The RM was diluted with brine (15 mL) and extracted with DCM (3 × 10 mL). The combined DCM extracts were dried over Na_2SO_4 and evaporated. The product was purified by

PTLC using EtOAc:Hex 1:4 + 2% Et_3N (the plate was developed twice to improve resolution). Product **13** was obtained as a white solid (32 mg, 60%).

^{1}H NMR (400 MHz, $CDCl_3$) δ 7.41 (s, 1H), 6.38 (d, *J* = 2.7 Hz, 2H), 4.30 (s, 1H), 3.91 (dd, *J* = 13.0, 10.4 Hz, 1H), 3.82 (s, 4H), 3.79 (s, 3H), 3.70 (s, 3H), 3.69 – 3.63 (m, 1H), 3.49 – 3.36 (m, 1H), 2.95 (dd, *J* = 27.1, 12.1 Hz, 2H), 2.54 – 2.38 (m, 2H), 2.39 – 2.22 (m, 3H), 2.13 (d, *J* = 14.2 Hz, 1H), 1.86 – 1.72 (m, 3H), 1.61 – 1.52 (m, 1H), 1.24 – 1.17 (m, 1H), 0.85 (t, J = 7.4 Hz, 3H).^{13}C NMR (101 MHz, $CDCl_3$) δ 169.2, 160.6, 157.2, 149.8, 135.1, 114.6, 111.8, 105.9, 103.9, 91.1, 81.1, 62.49, 61.8, 61.4, 60.9, 58.6, 55.8, 51.4, 50.3, 40.6, 40.2, 35.5, 24.3, 19.3, 13.2.HRMS (ESI+) calcd. for $C_{25}H_{34}ClN_2O_6^+$ $[M+H]^+$: 493.2105, found 493.2113.

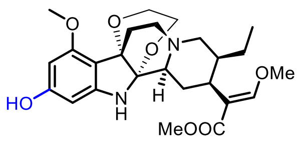

Compound 14 (11-OH-MG-EG). The boronate ester intermediate was prepared from **4** (50 mg, 0.11 mmol) as described in the general procedure C. The dark residue was dissolved in THF (0.7 mL) and 30% H_2O_2 (102 µL, 0.66 mmol) was added dropwise over 5–7 min at 0 °C. The reaction mixture was then stirred for 30 min at RT. The RM was then quenched with 5% $Na_2S_2O_3 \cdot 5H_2O$ (1 ml) and extracted with DCM (3 × 10 mL). The combined DCM extracts were dried over Na_2SO_4, evaporated, and the product was purified by PTLC using EtOAc:Hex 1:4 + 2% Et_3N (the plate was developed twice to improve resolution). Product **14** was obtained as a pale-brown solid (26 mg, 50%).

^{1}H NMR (400 MHz, $CDCl_3$) δ 7.40 (s, 1H), 5.96 – 5.83 (m, 2H), 4.23 (s, 1H), 3.94 – 3.83 (m, 2H), 3.80 (s, 3H), 3.78 (s, 3H), 3.70 (s, 3H), 3.64 (dd, *J* = 8.5, 2.3 Hz, 1H), 3.43 (d, *J* = 9.4 Hz, 1H), 3.00 (dd, *J* = 11.7, 2.1 Hz, 1H), 2.92 (dt, *J* = 12.9, 3.6 Hz, 1H), 2.49 – 2.43 (m, 2H), 2.39 – 2.19 (m, 4H), 2.11 (dt, *J* = 14.4, 2.5 Hz, 1H), 1.85 – 1.70 (m, 2H), 1.58 (d, *J* = 11.4 Hz, 1H), 1.31 (s, 1H), 0.85 (t, *J* = 7.3 Hz, 3H). ^{13}C NMR (101 MHz, $CDCl_3$) δ 169.2, 160.6, 158.4, 157.8, 149.6, 111.9, 108.0, 93.5, 91.5, 90.9, 81.1, 62.3, 61.9, 61.5, 61.4, 58.6, 55.4, 51.4, 50.7, 40.7, 40.1, 35.9, 24.3, 19.4, 13.2. HRMS (ESI+) calcd. for $C_{25}H_{35}N_2O_7^+$ $[M+H]^+$: 475.2444, found 475.2437.

<u>Deprotection of MG-EG adduct derivatives</u>

General procedure D: Reactions were performed according to a published procedure.[2]

Starting material (**5**, **7**, or **13**, 0.11 mmol) was dissolved in AcOH (2.0 mL) under argon and $NaBH_3CN$ (13.7 mg, 0.22 mmol) was added to the solution. After stirring at RT for 15 min, another portion of $NaBH_3CN$ (13.7 mg, 0.22 mmol) was added and stirring continued for 1 h. After this time, MeOH (81 µL) was added and the RM was heated to 90 °C for 14 h. The RM was added into a cold concentrated NH_4OH

solution and extracted with DCM. After drying over Na_2SO_4, the DCM extract was evaporated. Product was purified by PTLC using an appropriate solvent mixture as described for each derivative.

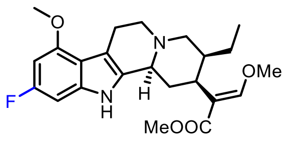

Compound 15 (11-F-MG). The reaction was performed according to the general procedure D except after addition of MeOH the RM was stirred for 1 h at 90 °C. The crude material was purified by PTLC using EtOAc:Hex 1:4 + 2% Et_3N. Product **15** was obtained as a yellow solid (32 mg, 70%).

^{1}H NMR (500 MHz, $CDCl_3$) δ 7.74 (s, 1H), 7.43 (s, 1H), 6.59 (dd, *J* = 9.3, 2.0 Hz, 1H), 6.26 (dd, *J* = 11.8, 2.0 Hz, 1H), 3.84 (s, 3H), 3.73 (s, 3H), 3.70 (s, 3H), 3.18 – 2.97 (m, 4H), 2.96 – 2.83 (m, 2H), 2.62 – 2.36 (m, 3H), 1.84 – 1.73 (m, 2H), 1.69 – 1.55 (m, 1H), 1.29 – 1.16 (m, 1H), 0.86 (t, *J* = 7.4 Hz, 3H). ^{13}C NMR (126 MHz, $CDCl_3$) δ 169.3, 161.1, 159.9 (d, *J* = 182.0 Hz), 154.7 (d, *J* = 12.4 Hz), 136.2 (d, *J* = 15.2 Hz), 133.8 (d, *J* = 3.3 Hz), 114.1, 111.6, 108.0, 90.3 (d, *J* = 26.3 Hz), 90.2 (d, *J* = 28.7 Hz), 61.7, 61.3, 57.9, 55.6, 53.8, 51.5, 40.8, 40.0, 30.1, 23.9, 19.2, 13.0. ^{19}F (proton decoupled) NMR (376 MHz, $CDCl_3$) δ -119.25. HRMS (ESI+): calcd. for $C_{23}H_{30}FN_2O_4^+$ $[M+H]^+$: 417.2256, found 417.2248.

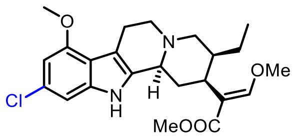

Compound 16 (11-Cl-MG). The reaction was performed according to the general procedure D. The crude material was purified by PTLC using EtOAc:Hex 1:4 + 2% Et_3N. Product **16** was obtained as a yellow solid (33 mg, 70%).

^{1}H NMR (400 MHz, $CDCl_3$) δ 7.84 (s, 1H), 7.42 (s, 1H), 6.89 (d, *J* = 1.6 Hz, 1H), 6.43 (d, *J* = 1.5 Hz, 1H), 3.85 (s, 3H), 3.72 (s, 3H), 3.70 (s, 3H), 3.15 – 2.97 (m, 4H), 2.95 – 2.86 (m, 2H), 2.61 – 2.33 (m, 3H), 1.85 – 1.70 (m, 2H), 1.66 – 1.57 (m, 1H), 1.32 – 1.16 (m, 1H), 0.86 (t, *J* = 7.4 Hz, 3H). ^{13}C NMR (101 MHz, $CDCl_3$) δ 169.3, 160.7, 154.6, 137.1, 134.3, 127.2, 116.5, 111.6, 108.2, 104.5, 101.4, 61.7, 61.3, 57.9, 55.6, 53.8, 51.5, 45.7, 40.8, 40.0, 23.8, 19.3, 13.0. LR-MS (APCI+) calcd. for $C_{23}H_{30}ClN_2O_4^+$ $[M+H]^+$: 433.2, found 433.3.

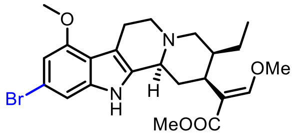

Compound 17 (11-Br-MG). The reaction was performed according to the general procedure D. The crude material was purified by PTLC using EtOAc:Hex 1:4 + 2% Et_3N. Product **17** was obtained as a yellow solid (42 mg, 80%).

^{1}H NMR (500 MHz, $CDCl_3$) δ 7.77 (s, 1H), 7.43 (s, 1H), 7.05 (d, J = 1.4 Hz, 1H), 6.55 (d, J = 1.5 Hz, 1H), 3.85 (s, 3H), 3.73 (s, 3H), 3.71 (s, 3H), 3.14 – 2.97 (m, 4H), 2.94 – 2.87 (m, 2H), 2.55 – 2.41 (m, 3H), 1.83 – 1.71 (m, 2H), 1.62 (dd, J = 8.6, 5.3 Hz, 1H), 1.24 – 1.15 (m, 1H), 0.86 (t, J = 7.4 Hz, 3H). ^{13}C NMR (126 MHz, $CDCl_3$) δ 169.3, 160.7, 154.7, 137.5, 134.3, 116.8, 114.5, 111.5, 108.3, 107.4, 104.0, 61.7, 61.2, 57.9, 55.7, 53.8, 51.5, 40.8, 40.0, 30.0, 23.9, 19.2, 13.0. LR-MS (APCI+) calcd. for $C_{23}H_{30}BrN_2O_4^+$ $[M+H]^+$: 477.2, found 477.4.

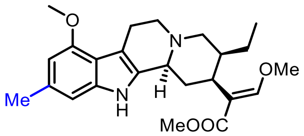

Compound 18 (11-CH_3-MG). Compound **9** (22 mg, 0.046 mmol) was dissolved in AcOH (1.0 mL) under argon and $NaBH_3CN$ (4.4 mg, 0.069 mmol) was added to the solution. After stirring at RT for 15 min, another portion of $NaBH_3CN$ (4.4 mg, 0.069 mmol) was added and the stirring continued for 2 h at room temperature until TLC-MS showed complete consumption of SM. The RM was added into a cold concentrated NH_4OH solution and extracted with DCM. After drying over Na_2SO_4, the DCM extract was evaporated. The crude material was purified by PTLC using EtOAc:Hex 1:4 + 2% Et_3N. Product **18** was obtained as a yellow solid (11.3 mg, 60%).

^{1}H NMR (500 MHz, $CDCl_3$) δ 7.53 (s, 1H), 7.43 (s, 1H), 6.69 (d, J = 1.0 Hz, 1H), 6.28 (d, J = 1.1 Hz, 1H), 3.85 (s, 3H), 3.72 (s, 3H), 3.70 (s, 3H), 3.14 – 3.11 (m, 1H), 3.10 – 3.05 (m, 2H), 3.04 – 2.97 (m, 1H), 2.96 – 2.86 (m, 2H), 2.56 – 2.41 (m, 3H), 2.40 (s, 3H), 1.83 – 1.72 (m, 2H), 1.60 – 1.58 (m, 1H), 0.88 – 0.84 (m, 1H), 0.86 (t, J = 7.4 Hz, 3H). ^{13}C NMR (126 MHz, $CDCl_3$) δ 169.4, 160.6, 154.3, 137.7, 133.1, 131.95, 115.7, 111.7, 107.8, 104.2, 101.8, 61.7, 61.4, 57.9, 55.4, 53.9, 51.5, 40.9, 40.1, 24.0, 22.2, 19.3, 13.0. LR-MS (APCI+): calcd. for $C_{24}H_{33}N_2O_4^+$ $[M+H]^+$: 413.5, found 413.8.

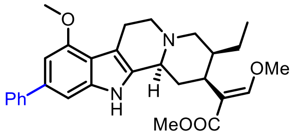

Compound 19 (11-Ph-MG). Compound **10** (64 mg, 0.12 mmol) was dissolved in AcOH (2.1 mL) under argon and $NaBH_3CN$ (14.6 mg, 0.23 mmol) was added to the solution. After stirring at RT for 1 h, MeOH (0.1 mL) was added and the RM was heated to 90 °C for 0.5 h. After cooling to RT, the RM was added into a cold concentrated NH_4OH solution and extracted with DCM. After drying over Na_2SO_4, the DCM extract

was evaporated. The crude material was purified by PTLC using EtOAc:Hex 1:4 + 2% Et_3N. Product **19** was obtained as a yellow solid (32 mg, 56%).

^1H NMR (500 MHz, $CDCl_3$) δ 7.73 (s, 1H), 7.65 – 7.57 (m, 2H), 7.44 (s, 1H), 7.43 – 7.39 (m, 2H), 7.32 – 7.27 (m, 1H), 7.12 (d, J = 1.2 Hz, 1H), 6.70 (d, J = 1.2 Hz, 1H), 3.94 (s, 3H), 3.74 (s, 3H), 3.71 (s, 3H), 3.22 – 3.08 (m, 2H), 3.07 – 2.97 (m, 3H), 2.97 – 2.90 (m, 1H), 2.59 – 2.50 (m, 2H), 2.49 – 2.41 (m, 1H), 1.87 – 1.74 (m, 1H), 1.69 – 1.58 (m, 2H), 1.22 (d, J = 12.0 Hz, 1H), 0.87 (t, J = 7.4 Hz, 3H). ^{13}C NMR (126 MHz, $CDCl_3$) δ 169.4, 160.7, 154.6, 143.0, 137.8, 135.8, 134.6, 128.7, 127.5, 126.6, 117.2, 111.7, 108.1, 103.2, 100.1, 61.7, 61.5, 57.9, 55.6, 53.9, 51.5, 40.9, 40.1, 30.1, 24.0, 19.3, 13.0. LR-MS (APCI+): calcd. for $C_{29}H_{35}N_2O_4^+$ $[M+H]^+$: 475.6, found 475.8.

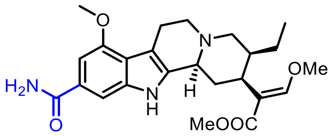

Compound 20 (11-$CONH_2$-MG). Compound **11** (37 mg, 74 µmol) was dissolved in AcOH (1.3 mL) under argon and $NaBH_3CN$ (9.4 mg, 0.15 mmol) was added to the solution. After stirring at RT for 15 min, another portion of $NaBH_3CN$ (9.4 mg, 0.15 mmol) was added and stirring continued for 1 h. MeOH (69 µL) was then added and the RM was heated to 90 °C for 2 h. After cooling to RT, the RM was added into a cold concentrated NH_4OH solution and extracted with DCM. After drying over Na_2SO_4, the DCM extract was evaporated. The crude material was purified by repeated PTLC using DCM:MeOH 95:5 and EtOAc:Hex:MeOH 1:1:0.1 + 2% Et_3N. Product **20** was obtained as an orange solid (11.4 mg, 35%).

^1H NMR (500 MHz, $CDCl_3$) δ 8.54 (s, 1H), 7.41 – 7.36 (m, 2H), 6.89 (s, 1H), 6.14 (s, 1H), 5.90 (s, 1H), 3.88 (s, 3H), 3.69 (d, J = 1.3 Hz, 6H), 3.08 (t, J = 13.7 Hz, 2H), 3.01 (td, J = 9.8, 2.9 Hz, 2H), 2.92 (td, J = 13.8, 12.7, 4.5 Hz, 2H), 2.49 (dd, J = 14.5, 10.1 Hz, 2H), 2.46 – 2.38 (m, 1H), 1.89 (d, J = 12.8 Hz, 1H), 1.80 – 1.70 (m, 1H), 1.64 – 1.57 (m, 1H), 1.23 – 1.14 (m, 1H), 0.85 (t, J = 7.3 Hz, 3H). ^{13}C NMR (126 MHz, $CDCl_3$) δ 171.1, 169.4, 160.8, 154.2, 137.3, 136.5, 126.7, 120.7, 111.5, 108.4, 105.0, 98.8, 61.7, 61.4, 57.9, 55.6, 53.7, 51.5, 40.7, 39.9, 29.8, 23.8, 19.3, 13.0. LR-MS (APCI+): calcd. for $C_{24}H_{32}N_3O_5^+$ $[M+H]^+$: 442.2, found 442.9.

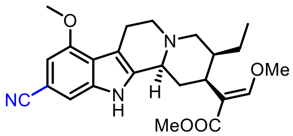

Compound 21 (11-CN-MG). Compound **12** (44 mg, 0.09 mmol) was dissolved in AcOH (0.8 mL) under argon and $NaBH_3CN$ (14.2 mg, 0.23 mmol) was added to the solution. After stirring at RT for 15 min, another portion of $NaBH_3CN$ (14.2 mg, 0.23 mmol) was added and stirring continued at 50 °C for 18 h.

The RM was then added into a cold concentrated NH_4OH solution and extracted with DCM. After drying over Na_2SO_4, the DCM extract was evaporated. The crude material was purified by PTLC using EtOAc:Hex 1:4 + 2% Et_3N. Product **21** was obtained as a yellow solid (13 mg, 34%).

^{1}H NMR (500 MHz, $CDCl_3$) δ 8.16 (s, 1H), 7.43 (s, 1H), 7.27 (d, J = 1.2 Hz, 1H), 6.62 (d, J = 1.2 Hz, 1H), 3.89 (s, 3H), 3.73 (s, 3H), 3.71 (s, 3H), 3.16 (dd, J = 11.5, 2.3 Hz, 1H), 3.11 – 2.99 (m, 3H), 2.97 – 2.88 (m, 2H), 2.58 – 2.48 (m, 2H), 2.47 – 2.41 (m, 1H), 1.83 (dt, J = 12.8, 3.1 Hz, 1H), 1.79 – 1.69 (m, 1H), 1.65 (s, 1H), 1.22 (td, J = 7.5, 6.4, 2.8 Hz, 1H), 0.87 (t, J = 7.3 Hz, 3H). ^{13}C NMR (126 MHz, $CDCl_3$) δ 169.3, 160.8, 154.4, 138.0, 135.9, 121.3, 121.3, 111.4, 110.1, 109.3, 103.6, 102.2, 61.8, 61.2, 57.8, 55.7, 53.6, 51.6, 40.7, 40.0, 29.9, 23.7, 19.2, 13.0. LR-MS (APCI+): calcd. for $C_{24}H_{30}N_3O_4^+$ $[M+H]^+$: 424.5, found 424.8.

Oxidation of MG derivatives to 7OH analogs

General procedure E: Starting material (73 µmol) was dissolved in acetone (2.2 mL), sat. aq. $NaHCO_3$ (1.5 mL) was added, and the stirred suspension was cooled in an ice bath (0 °C). Oxone (1.4 – 1.5 equiv) in H_2O (0.7 mL) was added dropwise over 20 min with vigorous stirring. (Care should be taken that the RM does not form lumps and should be stirred thoroughly). The reaction was monitored during the addition of oxone by TLC. After 25 min from the first addition, the RM was diluted with H_2O (10 mL) and extracted with EtOAc (3 × 10 mL). The combined extracts were washed with brine (10 mL), dried over Na_2SO_4, and concentrated. The product was purified by PTLC using an appropriate solvent mixture as described for each derivative.

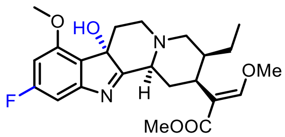

Compound 22 (11-F-7OH). The reaction was performed according to the general procedure E using SM **15** (30 mg, 73 µmol) and oxone (22.1 mg, 109 µmol). The crude material was purified by PTLC (EtOAc:Hex 1:4 + 2% Et_3N). Product **22** was obtained as a yellow solid (16 mg, 52%).

^{1}H NMR (400 MHz, $CDCl_3$) δ 7.43 (s, 1H), 6.91 (dd, J = 8.5, 2.0 Hz, 1H), 6.45 (dd, J = 11.3, 2.0 Hz, 1H), 3.84 (s, 3H), 3.80 (s, 3H), 3.69 (s, 3H), 3.12 (dd, J = 11.1, 2.6 Hz, 1H), 3.06 – 2.96 (m, 2H), 2.91 – 2.73 (m, 3H), 2.66 – 2.56 (m, 2H), 2.51 – 2.43 (m, 1H), 1.89 – 1.82 (m, 1H), 1.73 – 1.53 (m, 3H), 1.30 – 1.14 (m, 1H), 0.82 (t, J = 7.3 Hz, 3H). ^{13}C NMR (126 MHz, $CDCl_3$) δ 186.2, 169.4, 165.0 (d, J = 245.2 Hz), 160.9, 156.2 (d, J = 11.8 Hz), 156.1 (d, J = 13.1 Hz), 122.3, 111.4, 102.2 (d, J = 24.4 Hz), 97.1 (d, J = 27.4 Hz), 80.9, 61.9, 61.6, 58.3, 55.9, 51.5, 50.1, 40.6, 39.4, 36.1, 26.1, 19.1, 13.0. ^{19}F (proton decoupled) NMR (376 MHz, $CDCl_3$) δ -108.31. HRMS (ESI+) calcd. for $C_{23}H_{30}FN_2O_5^+$ $[M+H]^+$: 433.2139, found 433.2139.

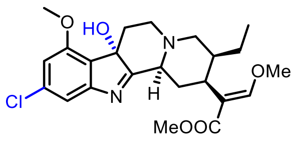

Compound 23 (11-Cl-7OH). The reaction was performed according to the general procedure E using SM **16** (30 mg, 69 µmol) and oxone (30 mg, 97 µmol). The crude material was purified by repeated PTLC (EtOAc:Hex 1:3 + 2% Et_3N and EtOAc:Hex 3:7 + 2% Et_3N). Product **23** was obtained as a yellow solid (15 mg, 50%).

^{1}H NMR (400 MHz, $CDCl_3$) δ 7.44 (s, 1H), 7.21 (d, *J* = 1.5 Hz, 1H), 6.73 (d, *J* = 1.5 Hz, 1H), 3.86 (s, 3H), 3.81 (s, 3H), 3.70 (s, 3H), 3.14 – 2.97 (m, 3H), 2.93 (d, *J* = 7.4 Hz, 1H), 2.87 – 2.72 (m, 2H), 2.70 – 2.55 (m, 2H), 2.48 (dd, *J* = 11.5, 3.0 Hz, 1H), 1.86 (d, *J* = 13.7 Hz, 1H), 1.75 – 1.54 (m, 3H), 1.33 – 1.17 (m, 1H), 0.82 (t, *J* = 7.3 Hz, 3H). ^{13}C NMR (126 MHz, $CDCl_3$) δ 185.8, 169.2, 160.7, 155.8, 135.9, 125.1, 114.9, 111.2, 109.6, 80.9, 61.8, 61.5, 58.1, 55.8, 49.9, 45.7, 40.5, 39.2, 35.8, 25.9, 18.9, 12.8. HRMS (ESI+) calcd. for $C_{23}H_{30}ClN_2O_5^+$ $[M+H]^+$: 449.1843, found 449.1842.

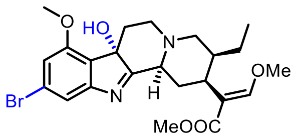

Compound 24 (11-Br-7OH). The reaction was performed according to the general procedure E using SM **17** (30 mg, 63 µmol) and oxone (25 mg, 82 µmol). The crude material was purified by repeated PTLC (EtOAc:Hex 1:3 + 2% Et_3N and EtOAc:Hex 3:7 + 2% Et_3N). Product **24** was obtained as a yellow solid (14 mg, 45%).

^{1}H NMR (400 MHz, $CDCl_3$) δ 7.43 (s, 1H), 7.34 (d, *J* = 1.3 Hz, 1H), 6.87 (d, *J* = 1.3 Hz, 1H), 3.86 (s, 3H), 3.80 (s, 3H), 3.69 (s, 3H), 3.15 – 2.96 (m, 3H), 2.84 – 2.71 (m, 2H), 2.65 – 2.56 (m, 2H), 2.51 – 2.44 (m, 1H), 2.30 (s, 1H), 1.85 (dt, *J* = 13.7, 3.1 Hz, 1H), 1.73 – 1.56 (m, 3H), 1.30 – 1.18 (m, 1H), 0.82 (t, *J* = 7.3 Hz, 3H). ^{13}C NMR (126 MHz, $CDCl_3$) δ 186.0, 169.4, 160.9, 156.1, 156.1, 125.8, 123.6, 118.0, 112.6, 111.3, 81.1, 61.9, 61.7, 58.3, 56.0, 51.4, 50.0, 40.6, 39.4, 35.8, 26.1, 19.1, 13.0. HRMS (ESI+) calcd. for $C_{23}H_{30}BrN_2O_5^+$ $[M+H]^+$: 493.1338, found 493.1337.

Activity of 11-X-7OH analogs at the human and rodent opioid receptors.

A/

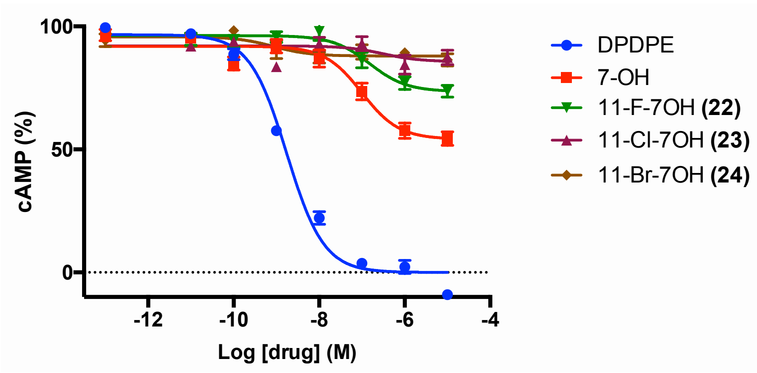

B/

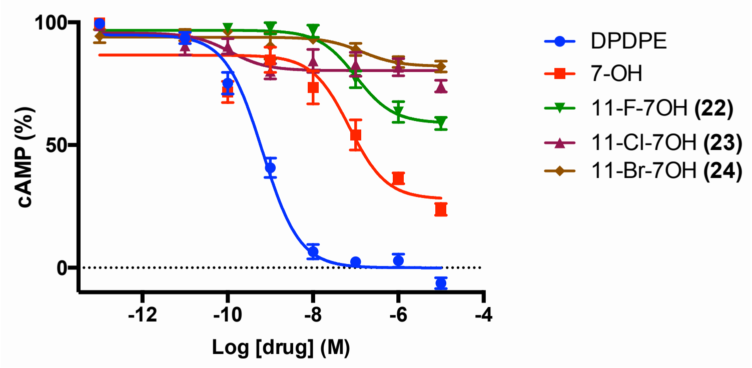

Supplementary Figure 7: Agonist activity of 11-X-7OH analogs at the human and mouse delta opioid receptor (hDOR and mDOR). hDOR and mDOR were co-expressed with $G\alpha_{oB}$, β1, γ2 and the BRET CAMYEL sensor. (A) Agonist activity at hDOR; positive control = [D-Pen^{2,5}]-enkephalin (DPDPE) (B) Agonist activity at mDOR, positive control = DPDPE; Curves represent the average of n = 3, independent experiments with error bars representing ± SEM.

A/

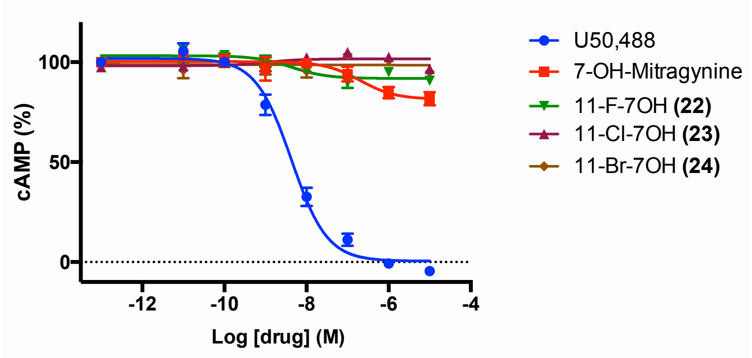

B/

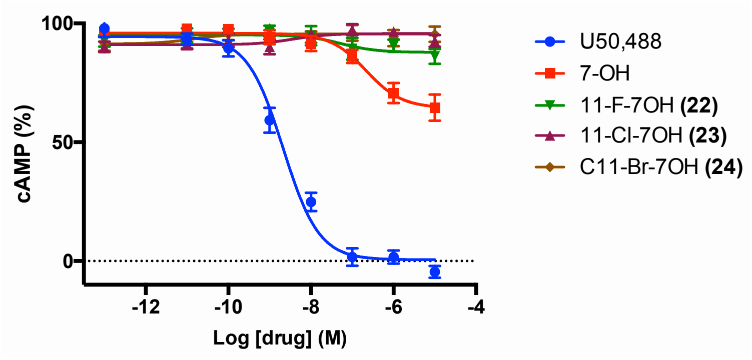

Supplementary Figure 8: Agonist activity of **11-X-7OH** analogs at the human and rat kappa opioid receptor (hKOR and rKOR). hKOR and rKOR were co-expressed with Gα_{oB}, β1, γ2 and the BRET CAMYEL sensor. (A) Agonist activity at hKOR; positive control = U-50,488. (B) Agonist activity at rKOR, positive control = U-50,488; Curves represent the average of *n* = 3, independent experiments with error bars representing ± SEM.

A/

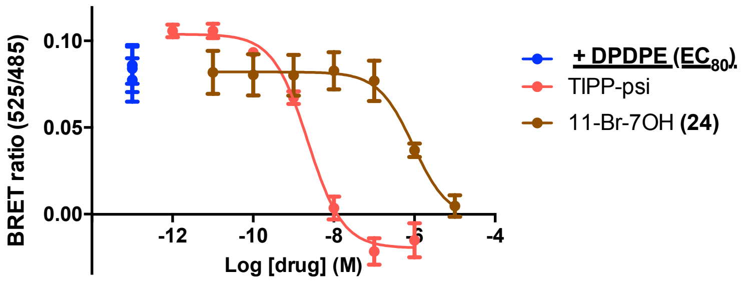

B/

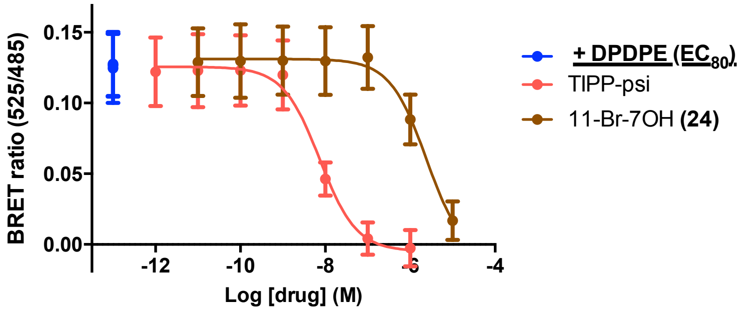

Supplementary Figure 9: Antagonist activity of **11-Br-7OH** (**24**) analog at the human and mouse delta opioid receptor (hDOR and mDOR). hDOR and mDOR were co-expressed with Gα_{oB}, β1, γ2 and the BRET CAMYEL sensor. (A) Antagonist activity at hDOR, competitive inhibition of [D-Pen^{2,5}]-enkephalin (DPDPE), positive control = TIPP-psi. (B) Antagonist activity at mDOR, competitive inhibition of DPDPE, positive control = TIPP -psi. Curves represent the average of *n* = 3, independent experiments with error bars representing ±SEM.

A/

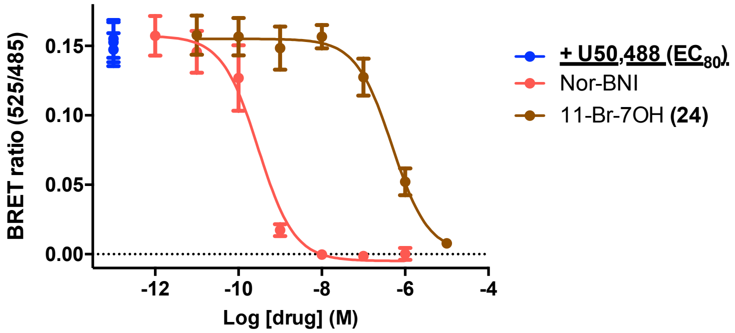

B /

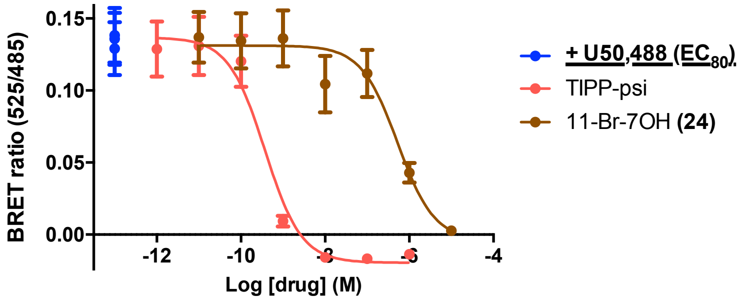

Supplementary Figure 10: Antagonist activity of **11-Br-7OH** (**24**) analog at the human and rat kappa opioid receptor (hKOR and rKOR). hKOR and rKOR were co-expressed with $G\alpha_{oB}$, β1, γ2 and the BRET CAMYEL sensor. (A) Antagonist activity at hKOR, competitive inhibition of U-50,488 (EC_{80}), positive control = nor-binaltorphimine (nor-BNI). (B) Antagonist activity at rKOR, competitive inhibition of U-50,488 (EC_{80}), positive control = TIPP-psi. Curves represent the average of *n* = 3, independent experiments with error bars representing ±SEM.

Biological Procedures:

Mouse Receptor K_i Determination.

Materials: IBNtxA and [^{125}I]BNtxA were synthesized at MSKCC as previously described.[9–11] Na^{125}I was purchased from Perkin-Elmer (Waltham, MA).

Radioligand Competition Binding Assays with Mouse Receptors: [^{125}I]BNtxA binding was carried out in membranes prepared from Chinese Hamster Ovary (CHO) cells stably expressing murine clones of MOR, DOR, and KOR, as previously described.[9,10,12] Binding incubations were performed at 25 °C for 90 min in 50 mM potassium phosphate buffer, pH 7.4, containing 5 mM magnesium sulfate. After the incubation, the reaction was filtered through glass-fiber filters (Whatman Schleicher & Schuell, Keene, NH) and washed three times with 3 mL of ice-cold 50 mM Tris-HCl, pH 7.4, on a semiautomatic cell harvester. Nonspecific binding was defined by addition of levallorphan (8 μM) to matching samples and was subtracted from total binding to yield specific binding. K_i values were calculated by nonlinear

regression analysis (GraphPad Prism, San Diego, CA). Protein concentrations were determined using the Lowry method with BSA as the standard.[13]

CAMYEL BRET Assays.

Materials. HEK-293T cells were obtained from the American Type Culture Collection (Rockville, MD, USA) and were cultured in a 5% CO_2 atmosphere at 37 °C in Dulbecco's Modified Eagle Medium (high glucose no. 11965; Life Technologies; Grand Island, NY, USA) supplemented with 10% Fetal Bovine Serum and 100 U ml^{-1} penicillin and 100 µg ml^{-1} streptomycin (Corning). DAMGO and nor-BNI were purchased from Abcam (Cambridge, United Kingdom). Coelenterazine H was purchased from Dalton Pharma Services (Toronto, ON, Canada). Polyethylenimine (PEI) was purchased from Polysciences (Warrington, PA, USA). Forskolin was purchased from Sigma-Aldrich (Saint Louis, MO, USA). DPDPE, naloxone HCl, and (±)-U-50488 HCl were purchased from Tocris Biosciences (Minneapolis, MN, USA). TIPP[psi] was obtained from the National Institute on Drug Abuse Drug Supply Program.

DNA Constructs. The mouse MOR (mMOR), the mouse DOR (mDOR) and the rat KOR (rKOR) were provided by Dr Lakshmi Devi at Mount Sinai School of Medicine. The human MOR (hMOR), human DOR (hDOR) and human KOR (hKOR) were obtained from the Missouri S&T Resource Center. The human G protein constructs used here have been previously described[14] and were obtained from the Missouri S&T Resource Center unless otherwise noted. The G proteins used were: $G\alpha_{oB}$ ($G\alpha_{oB}$), $G\beta_1$ (β_1), $G\gamma_2$ ($\gamma 2$). YFP-Epac-RLuc (CAMYEL) was obtained from ATCC (no. MBA-277).[15] All constructs were sequence confirmed.

Transfection. A total of 20 µg of cDNA was transiently transfected into HEK-293T cells (6×10^6 cells per plate) in 10 cm dishes (1.25 µg receptor, 10 µg CAMYEL, 1.25 µg $G\alpha_{oB}$, 1.25 µg β_1, 1.25 µg $\gamma 2$, and 5µg pcDNA3.1 empty vector) using PEI in a 1:1 ratio (diluted in Opti-MEM, Life Technologies). Cells were maintained in the HEK-293T media described above. After 24 h, the media was changed, and the experiment was performed 24 h later (48 h after transfection).

Bioluminescence Resonance Energy Transfer (BRET). Experiments were performed as described previously.[14,16–18] Briefly, transfected cells were dissociated and resuspended in phosphate-buffered saline. Approximately 200,000 cells per well were added to a black-framed, white well 96-well plate (no. 60050; Perkin Elmer; Waltham, MA, USA). For agonist assays, 1 µM forskolin (cAMP accumulation) was added to each well. After 5 min, the luciferase substrate coelenterazine H (5 µM) was added to each well. Ligands were added after 5 min, then BRET signal was measured 5 min later. For antagonist assays, ligands were added first, then allowed to incubate for 20 min. Following antagonist incubation, forskolin (1 µM) was added to each well. After 5 min, coelenterazine H (5 µM) was added to

each well. After another 5 min, full agonist ligands (DAMGO, DPDPE, or U-50,488 at EC_{80} concentration) were added to each well and the BRET signal was measured 5 min later. All BRET signal measurements were done on a PHERAstar FS plate reader (BMG Labtech, Cary, NC, USA). The BRET signal was calculated as the ratio of the light emitted by the energy acceptor, mVenus (510–540 nm), over the light emitted by the energy donor, RLuc8 (485 nm). Dose–response curves were fit using a three-parameter logistic equation in GraphPad Prism 5 (Graphpad Software, La Jolla, CA, USA). All experiments were repeated in three independent trials each with triplicate determinations.

BRET-Based Nb33 Recruitment Assays.

Materials: HEK-293T cells were obtained from the American Type Culture Collection (Rockville, MD, USA) and were cultured in a 5% CO_2 atmosphere at 37 °C in Dulbecco's Modified Eagle Medium (DMEM, high glucose, #11965; Life Technologies; Grand Island, NY, USA) supplemented with 10% Fetal Bovine Serum (#35-010-CV, Corning, Corning, NY, USA) and 100 IU ml^{-1} penicillin and 100 μg ml^{-1} streptomycin (#30-002-CI; Corning, Corning, NY, USA). The following chemicals were used without further modification: DAMGO (#78123-71-4, Abcam, Cambridge, United Kingdom), buprenorphine hydrochloride (#B9275, Sigma-Aldrich, St. Louis, MO, USA), morphine sulfate (#M1167, Spectrum Chemicals, New Brunswick, NJ, USA), coelenterazine H (#DC-001437, Dalton Pharma Services, Toronto, ON, Canada), PEI (#NC1014320, Polysciences, Warrington, PA, USA).

DNA Constructs: The expression vector coding for mouse MOR tagged at the C-terminus with Nanoluc (mMOR-nluc) by a Gly-Ser linker was constructed using standard techniques in molecular biology and confirmed by DNA sequencing (Psomagen, Brooklyn, NY, USA). Briefly, two DNA inserts were PCR amplified, one coding for mMOR with an N terminal signal peptide followed by a FLAG tag, and the other coding for NanoLuc. The two inserts were joined by PCR amplification and the resulting insert coding mMOR-nluc was cloned into the Hind III and Xho I sites of pcDNA3.1 (+) (#V79020, ThermoFisher Scientific, Waltham, MA, USA). The plasmid coding for human MOR-nanoluc (hMOR-nluc) was a gift from Dr. Nevin Lambert at the Medical College of Georgia. The plasmid coding for the nanobody-33-Venus (Nb-33) construct[19] was a gift from Dr. Meritxell Canals at the University of Nottingham.

Transfection: A total of 5 μg of cDNA was transiently transfected into HEK-293T cells (2 × 106 cells per plate) in 10 cm dishes (1 μg receptor-nluc, and 4 μg Nb-33-Venus), using PEI in a 6:1 ratio (diluted in DMEM). Cells were maintained in the HEK-293T media described above. Experiments were performed 48 h after transfection.

BRET: Experiments were performed as described previously.[20] Briefly, transfected cells were dissociated and resuspended in phosphate-buffered saline. Cells were added to a black-framed, white well

96-well plate (no. 60050; Perkin Elmer; Waltham, MA, USA). At time zero, the luciferase substrate coelenterazine H (5 μM) was added to each well. Ligands were added after 5 min, then BRET signal was measured 10 min later. BRET measurements were performed using a PHERAstar FS plate reader (BMG Labtech, Cary, NC, USA). The BRET signal was calculated as the ratio of the light emitted by the mVenus acceptor (510–540 nm) over the light emitted by the NanoLuc donor (475 nm). Dose–response curves were fit using a three-parameter logistic equation in GraphPad Prism 8 (Graphpad Software, La Jolla, CA, USA). All experiments were repeated in at least three independent trials each with triplicate determinations.

Tail Flick Mice assay.

Mice

For analgesic dose-response experiments, male CD1 mice (20–32 g),6-8 weeks were obtained from Charles River Laboratories and male C57BL/6 mice (22–30 g), 8–15 weeks were obtained from Jackson Lab (Bar Harbor, ME) and housed 5 mice per cage in a vivarium following an IACUC-approved protocol. For male C57BL/6 mice temperature was kept constant at 22 ± 2 °C, and relative humidity was maintained at 50 ± 5%. For male CD1 mice (20–32 g) the temperature was in the range of 20-26 °C and relative humidity maintained within the range of 30-70%. Mice were given access to food and tap water *ad libitum*. All mice used throughout the manuscript were opioid naïve. All mice were maintained on a 12 h light/dark cycle with Purina rodent chow and water available ad libitum and housed in groups of five until testing.

For analgesic testing in knockout animals, wild-type, male C57BL/6 mice (22–33 g), 10-12 weeks were purchased from the Jackson Lab (Bar Harbor, ME). These mice were kept at a constant temperature of 22 ± 2 °C, and relative humidity was maintained at 40-50%. Exon-1/Exon-11 MOR-1 KO mice on a C57 background were bred in the Pintar laboratory at Rutgers University. All mice were maintained on a 12-hour light/dark cycle with food and water available *ad libitum*, and housed in groups of five until testing. All testing was done in the light cycle.

All animal studies were preapproved by the Institutional Animal Care and Use Committees of Washington University School of Medicine and Columbia University, in accordance with the 2002 National Institutes of Health Guide for the Care and Use of Laboratory Animals.

Materials and Formulations

Compounds 7OH and the analogs were prepared as described above. Compound solutions were prepared on the same day as testing from pure solid material. For the analgesic testing with the C57BL/6 mice the solids were dissolved in UPS grade 0.85% saline with addition of 2 molar equivalents of glacial acetic acid. For the analgesic testing with the CD1 mice solids were dissolved in 10:10:80 of DMSO: solutol: saline solution. Heat and sonication were used to assist in fully dissolving the solid to obtain a clear solution. A

dilution series was prepared for the desired doses of 0.1, 0.3, 1, 3, and 10 mg/kg (concentrations 0.01 mg/mL, 0.03 mg/mL, 0.1 mg/mL, 0.3 mg/mL, and 1 mg/mL respectively). Solutions were filtered through a PTFE 0.45μm syringe filter and administered s.c. at a max volume of 300μL.

Tail Flick (Dose – response)

Tail flick antinociception was determined using the radiant heat tail flick technique using an Ugo Basile model 37360 instrument as previously described.[21,22] The intensity was set to achieve a baseline between 2 and 3 s. Baseline latencies were determined before experimental treatments for all mice. Tail flick antinociception was assessed as an increase in baseline latency, with a maximal 15 s latency to minimize damage to the tail. Data were analyzed as percent maximal effect, %MPE, and was calculated according to the formula: % MPE = [(observed latency – baseline latency)/(maximal latency – baseline latency)] × 100. Compounds were injected subcutaneously (s.c.) and antinociception was assessed at the peak effect. Mice were tested for analgesia with cumulative subcutaneous doses of the drug until the mouse can withstand the maximal latency. Once the mouse reached the maximal latency, the mouse was no longer given higher doses. The analgesia experiments were performed by blinding the experimenter to the identity of 7OH versus 11-F-7OH. *In vivo* experiments were evaluated using GraphPad Prism 8, San Diego, CA as described above.

Tail Flick (KO animals)

Analgesia was tested in wild-type and MOR KO animals by the radiant heat tail-flick technique using an IITC Model 33 Tail Flick Analgesia Meter as previously described.[23] The intensity was set to achieve a baseline between 2 and 3 s. Tail flick antinociception was assessed as an increase in baseline latency, with a maximal 10 s latency to minimize damage to the tail. Data were analyzed as percent maximal effect, %MPE, which was calculated according to the formula: % MPE [(observed latency − baseline latency)/(maximal latency − baseline latency)] × 100. Compounds were administered subcutaneously (s.c.) as indicated in the figures, and analgesia was assessed at the peak effect (15 min). Mice were tested for analgesia with cumulative subcutaneous doses of the drug until the mouse can withstand the maximal latency. Once the mouse reached the maximal latency, the mouse was no longer given higher doses. *In vivo* experiments were evaluated using GraphPad Prism 8, San Diego, CA as described above.

Supplementary References:

1. Kruegel, A. C. *et al.* Synthetic and Receptor Signaling Explorations of the *Mitragyna* Alkaloids: Mitragynine as an Atypical Molecular Framework for Opioid Receptor Modulators. *Journal of the American Chemical Society* **138**, 6754–6764 (2016).
2. Takayama, H. *et al.* New Procedure to Mask the 2,3-π Bond of the Indole Nucleus and Its Application to the Preparation of Potent Opioid Receptor Agonists with a Corynanthe Skeleton. *Organic Letters* **8**, 5705–5708 (2006).
3. Gotoh, H., Duncan, K. K., Robertson, W. M. & Boger, D. L. 10′-Fluorovinblastine and 10′-Fluorovincristine: Synthesis of a Key Series of Modified Vinca Alkaloids. *ACS Medicinal Chemistry Letters* **2**, 948–952 (2011).
4. Kruegel, A. C. *et al.* 7-Hydroxymitragynine Is an Active Metabolite of Mitragynine and a Key Mediator of Its Analgesic Effects. *ACS Central Sciemce* **5**, 992–1001 (2019).
5. Váradi, A. *et al.* Mitragynine/Corynantheidine Pseudoindoxyls As Opioid Analgesics with Mu Agonism and Delta Antagonism, Which Do Not Recruit β-Arrestin-2. *Journal of Medicinal Chemistry* **59**, 8381–8397 (2016)
6. Zacharias, D. E., Rosenstein, R. D. & Jeffrey, G. A. The structure of mitragynine hydroiodide. *Acta Crystallography* **18**, 1039–1043 (1965)
7. Carvalho, P., Furr III, E. B. & McCurdy, C. (*E*)-Methyl 2-[(2 *S* ,3 *S* ,12b *R*)-3-ethyl-8-methoxy-1,2,3,4,6,7,12,12b-octahydroindolo[2,3- *a*]quinolizin-2-yl]-3-methoxyacrylate ethanol solvate. *Acta Crystallogr. Sect. E Struct. Rep. Online* **65**, o1441–o1442 (2009).
8. Furuya, T., Strom, A. E. & Ritter, T. Silver-Mediated Fluorination of Functionalized Aryl Stannanes. *Journal of the American Chemical Society* **131**, 1662–1663 (2009).
9. Majumdar, S. *et al.* Generation of novel radiolabeled opiates through site-selective iodination. *Bioorganic and Medicinal Chemistry Letters* **21**, 4001–4004 (2011).
10. Pickett, J. E. *et al.* Mild, Pd-catalyzed stannylation of radioiodination targets. *Bioorganic and Medicinal Chemistry Letters* **25**, 1761–1764 (2015)

11. Majumdar, S. *et al.* Truncated G protein-coupled mu opioid receptor MOR-1 splice variants are targets for highly potent opioid analgesics lacking side effects. *Proceedings of the National Academy Sciences* **108**, 19778–19783 (2011).

12. Pan, Y.-X. *et al.* Identification and Characterization of Three New Alternatively Spliced μ-Opioid Receptor Isoforms. *Molecular Pharmacology* **56**, 396–403 (1999).

13. Lowry, O. H., Rosebrough, N. J., Farr, A. L. & Randall, R. J. Protein measurement with the Folin phenol reagent. *Journal of Biological Chemistry* **193**, 265–275 (1951).

14. Rives, M.-L., Rossillo, M., Liu-Chen, L.-Y. & Javitch, J. A. 6′-Guanidinonaltrindole (6′-GNTI) Is a G Protein-biased κ-Opioid Receptor Agonist That Inhibits Arrestin Recruitment. *Journal of Biological Chemistry* **287**, 27050–27054 (2012).

15. Jiang, L. I. *et al.* Use of a cAMP BRET Sensor to Characterize a Novel Regulation of cAMP by the Sphingosine 1-Phosphate/G $_{13}$ Pathway. *Journal of Biological Chemistry* **282**, 10576–10584 (2007).

16. Gassaway, M. M., Rives, M.-L., Kruegel, A. C., Javitch, J. A. & Sames, D. The atypical antidepressant and neurorestorative agent tianeptine is a μ-opioid receptor agonist. *Translational Psychiatry* **4**, e411–e411 (2014).

17. Besson, A. *et al.* Discovery of an oncogenic activity in p27Kip1 that causes stem cell expansion and a multiple tumor phenotype. *Genes and Development* **21**, 1731–1746 (2007).

18. Vezzi, V. *et al.* Ligands Raise the Constraint That Limits Constitutive Activation in G Protein-coupled Opioid Receptors. *Journal of Biological Chemistry* **288**, 23964–23978 (2013).

19. Gillis, A. *et al.* Low intrinsic efficacy for G protein activation can explain the improved side effect profiles of new opioid agonists. *Science Signalling* **13**, eaaz3140–2159 (2020).

20. Donthamsetti, P., Quejada, J. R., Javitch, J. A., Gurevich, V. V. & Lambert, N. A. Using Bioluminescence Resonance Energy Transfer (BRET) to Characterize Agonist-Induced Arrestin Recruitment to Modified and Unmodified G Protein-Coupled Receptors. *Current Protocols in Pharmacology* **70**, 2141–2144 (2015).

21. Kozai, T. D. Y., Jaquins-Gerstl, A. S., Vazquez, A. L., Michael, A. C. & Cui, X. T. Brain Tissue Responses to Neural Implants Impact Signal Sensitivity and Intervention Strategies. *ACS Chemical Neuroscience* **6**, 48–67 (2015).

22. Váradi, A. *et al.* Synthesis of Carfentanil Amide Opioids Using the Ugi Multicomponent Reaction. *ACS Chemical Neuroscience* **6**, 1570–1577 (2015).

23. Schuller, A. G. P. *et al.* Retention of heroin and morphine–6β–glucuronide analgesia in a new line of mice lacking exon 1 of MOR–1. *Nature Neuroscience* **2**, 151–156 (1999).

ACRONYMS USED:

dtbpy: 4,4′-di-tert-butyl-2,2′-dipyridyl
Me_4-phen: 3,4,7,8-tetramethyl-1,10-phenanthroline
PG: protecting group
RT: Room Temperature
RM: Reaction Mixture
TLC: Thin layer Chromatography
SM: Starting Material
PTLC: Preparative Thin Layer Chromatography
DAMGO: [D-Ala^2, N-Me-Phe^4, Gly^5-ol]-enkephalin
Nor-BNI: Nor-binaltorphimine

NMR Spectra

Supplementary Figure 11 : ^{1}H-NMR of Boc-MG

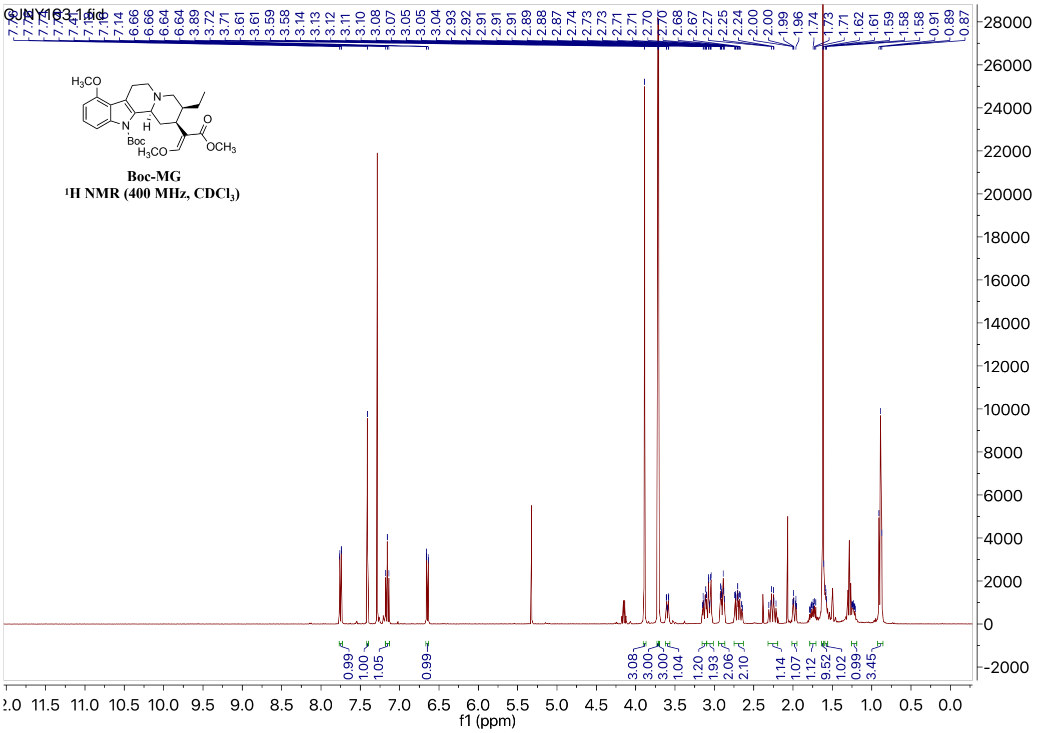

Supplementary Figure 12 : ^{13}C-NMR of Boc-MG

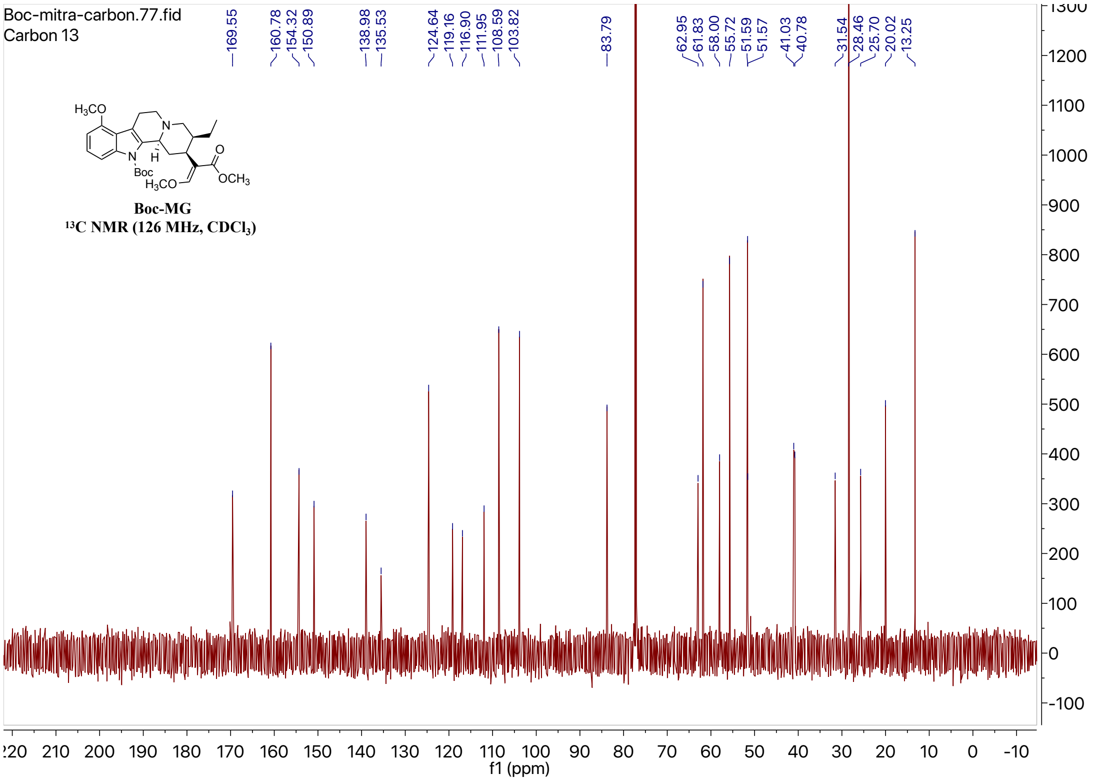

Supplementary Figure 13 : ^{1}H-NMR of 12-Br-MG (**3**)

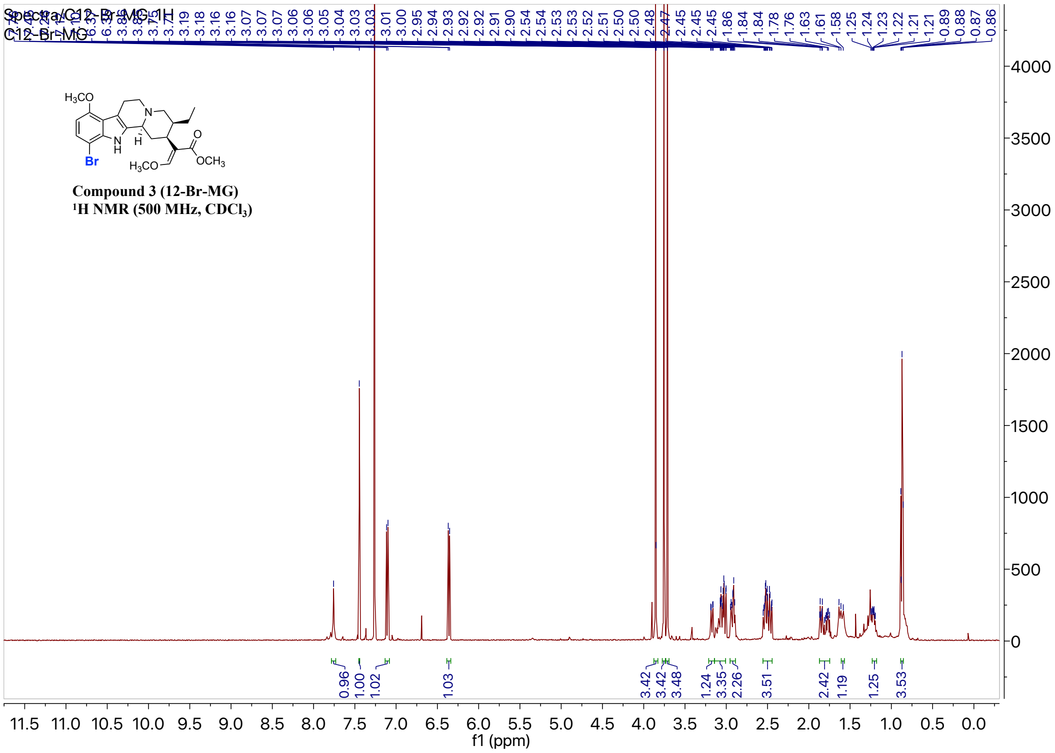

Supplementary Figure 14 : ^{13}C-NMR of 12-Br-MG (**3**)

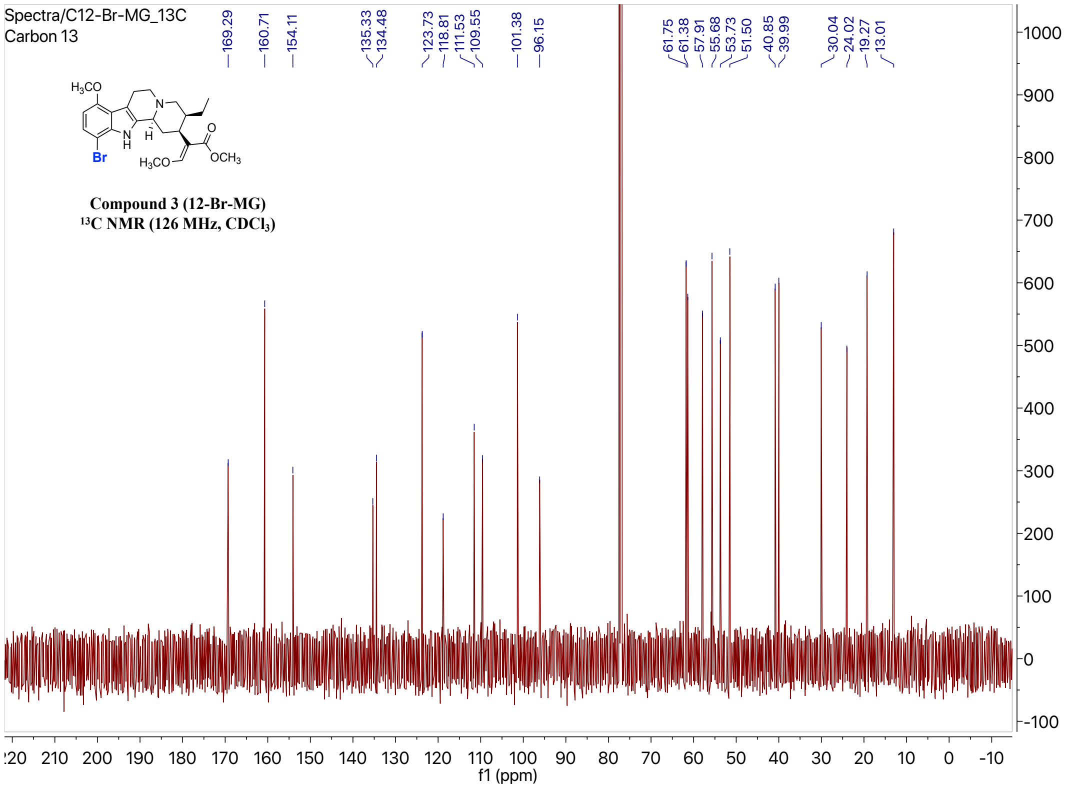

Supplementary Figure 15 : ^{1}H-NMR of Dihydromitragynine (**DHM**)

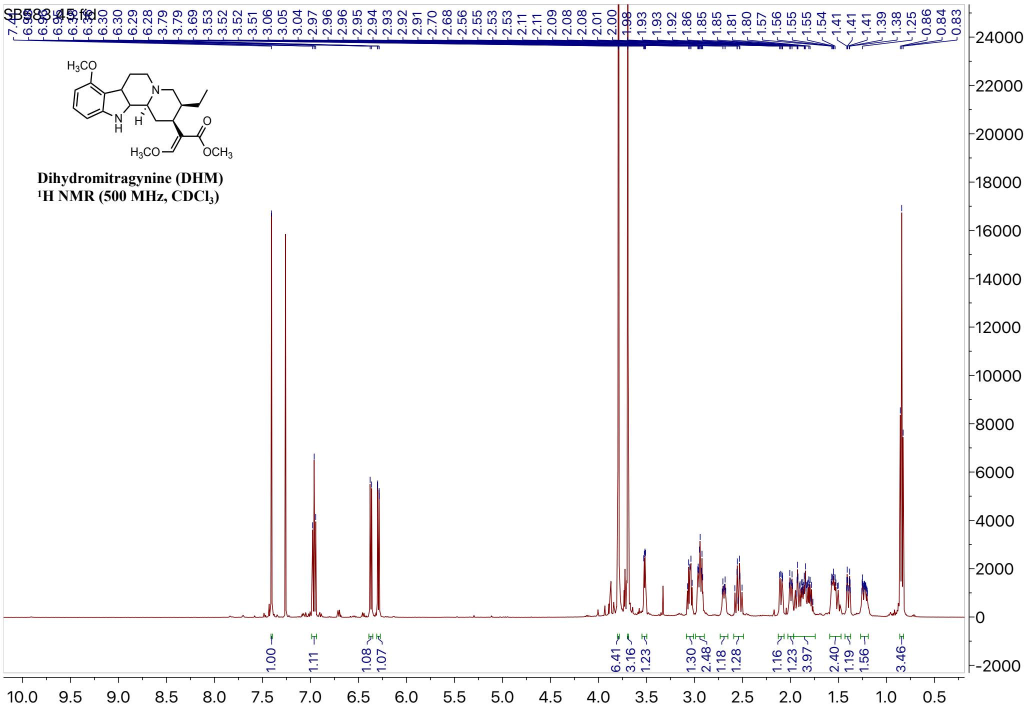

Supplementary Figure 16 : ^{13}C-NMR of Dihydromitragynine (**DHM**)

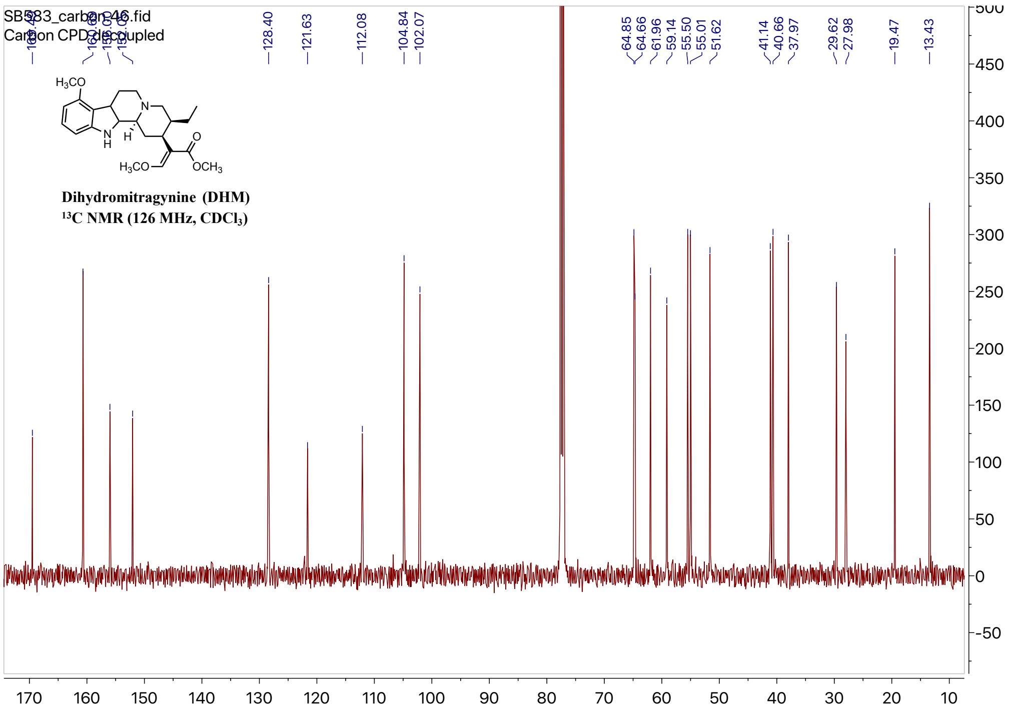

Supplementary Figure 17 : ^{1}H-NMR of MG EG (**4**)

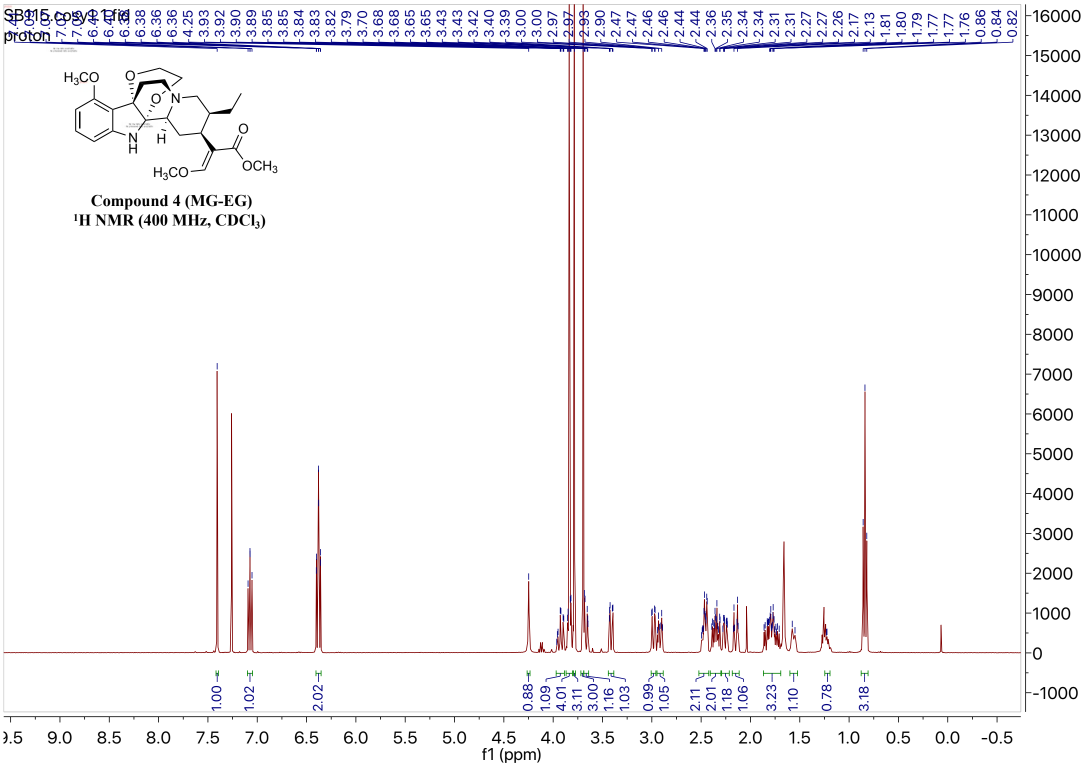

Supplementary Figure 18 : ^{13}C-NMR of MG EG (**4**)

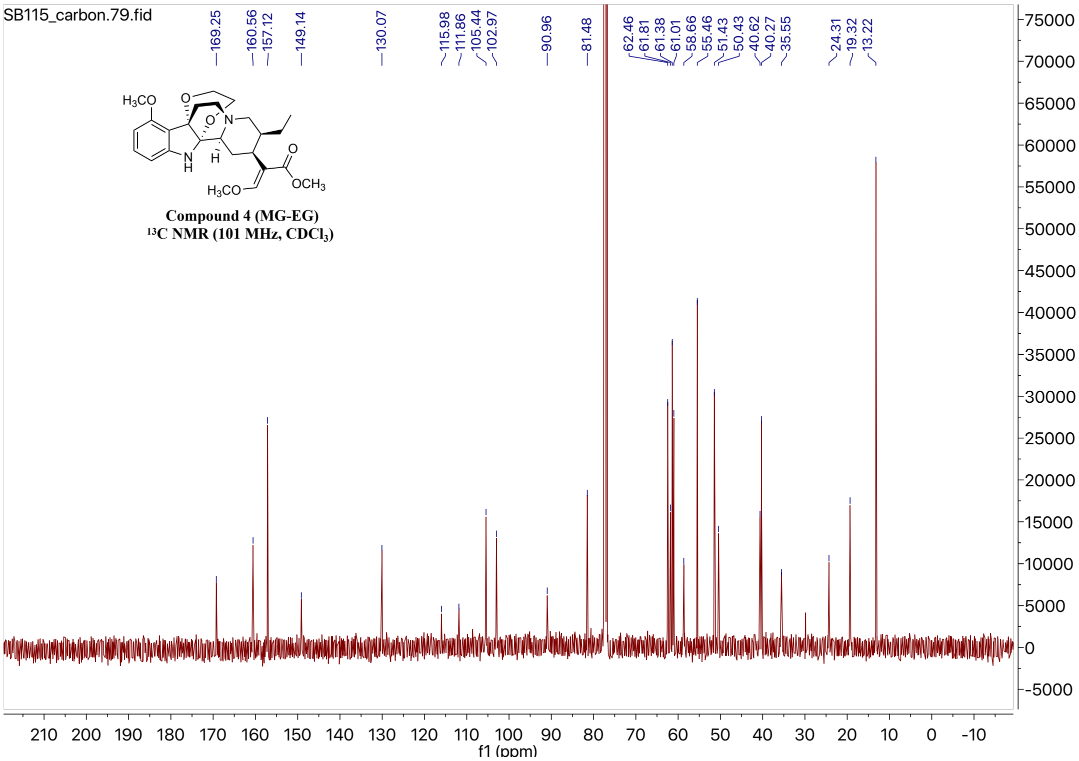

Supplementary Figure 19 : ^{1}H-NMR of **4** and **5** (mixture)

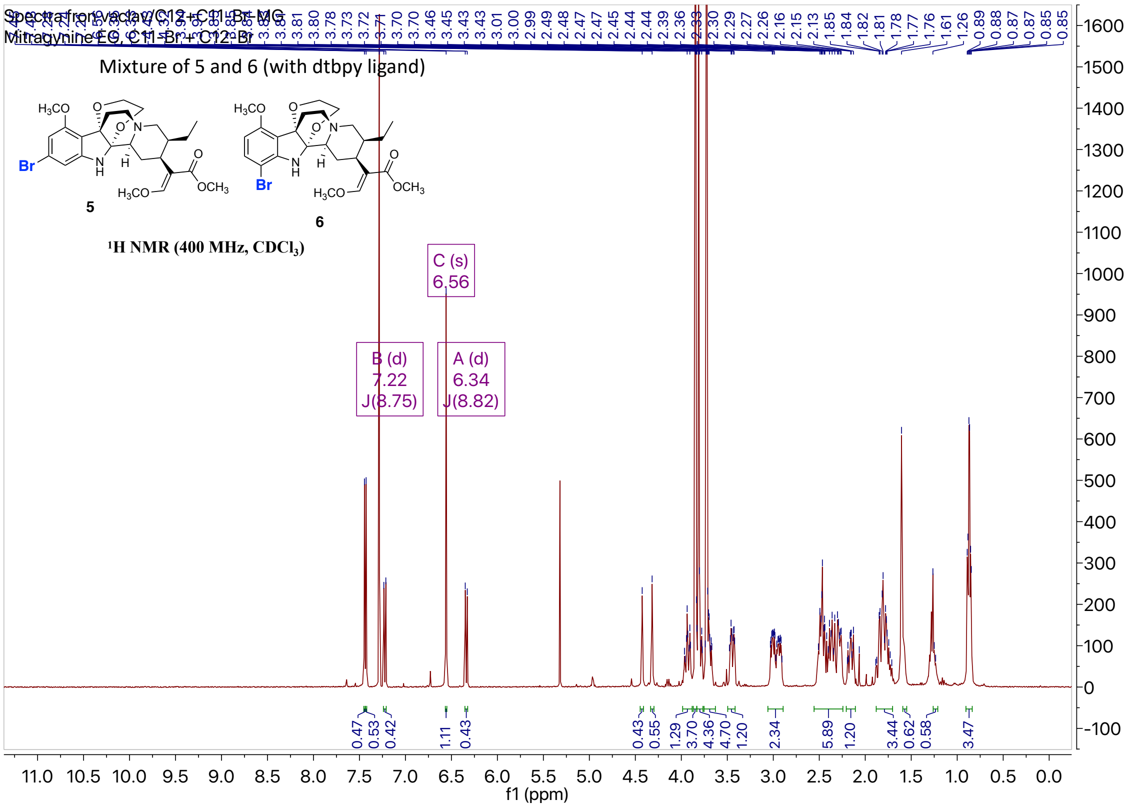

Supplementary Figure 20 : ^{1}H-NMR of 11-Br-MG EG (**5**)

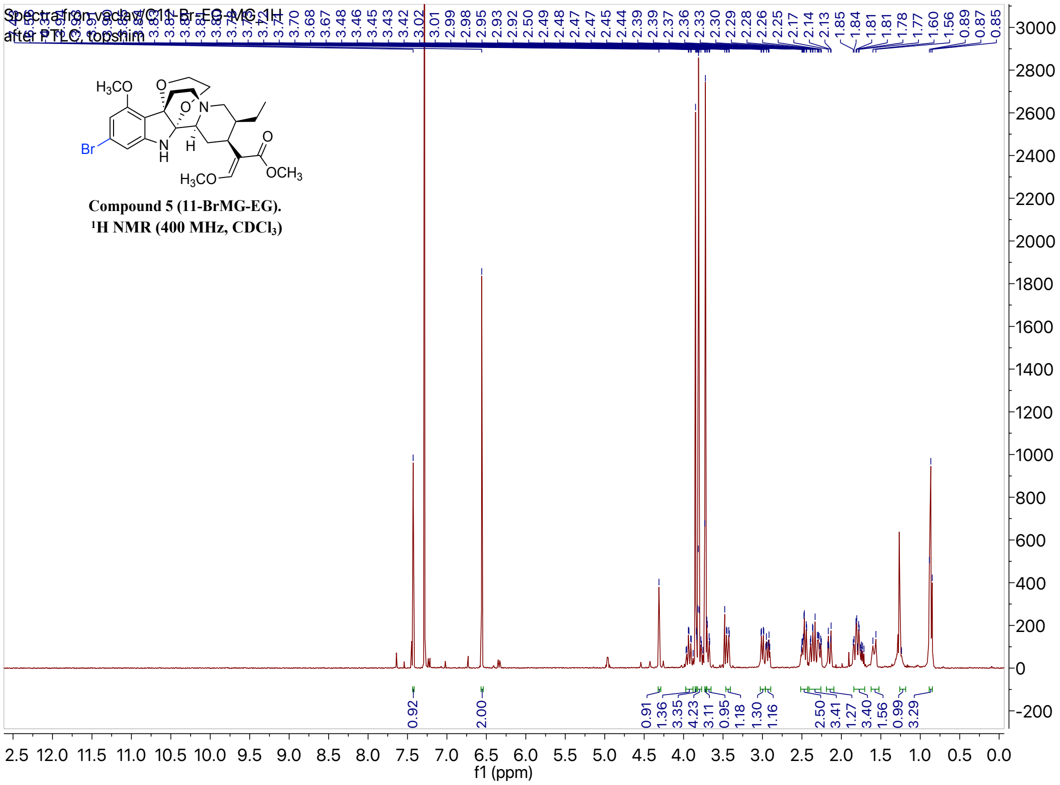

Supplementary Figure 21 : ^{13}C-NMR of 11-Br-MG EG (**5**)

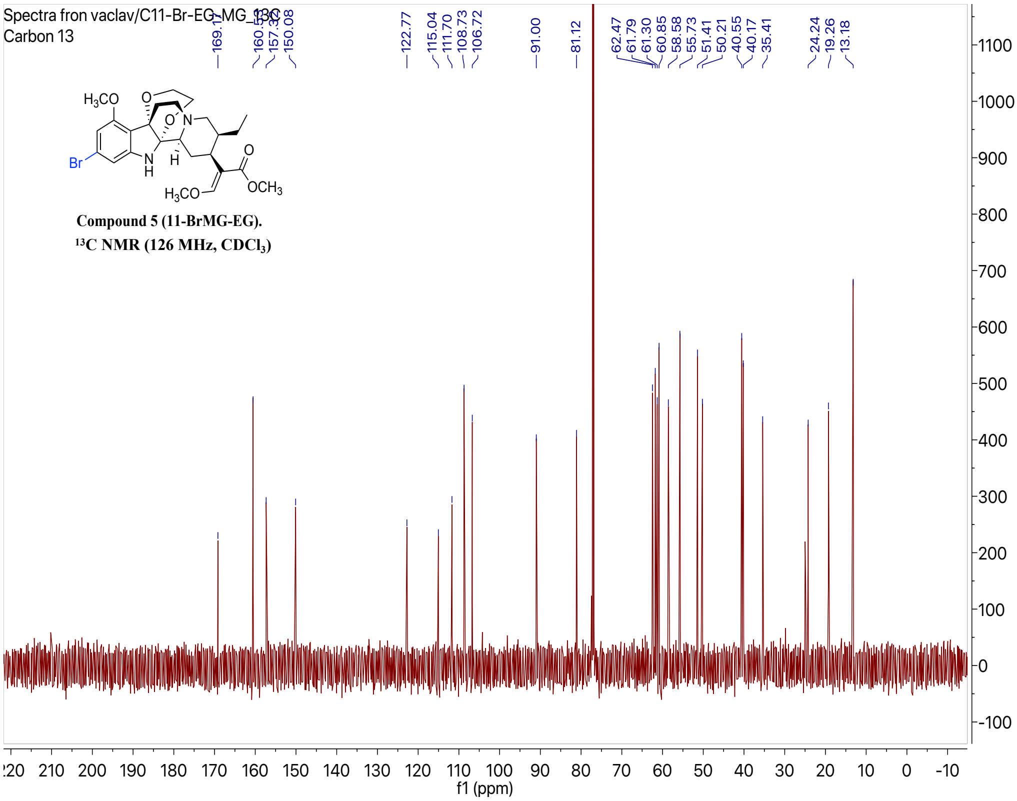

Supplementary Figure 22 : ^{1}H-NMR of 11-F-MG EG (**7**)

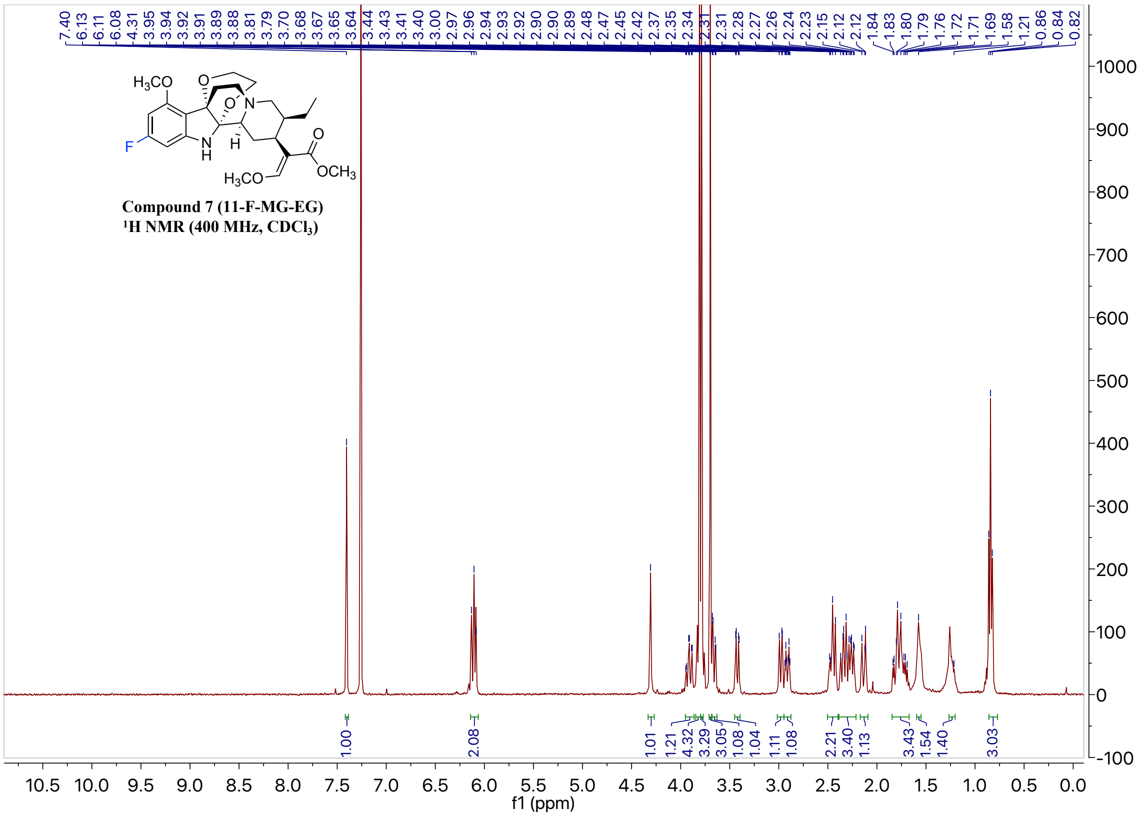

Supplementary Figure 22 : ^{13}C-NMR of 11-F-MG EG (**7**)

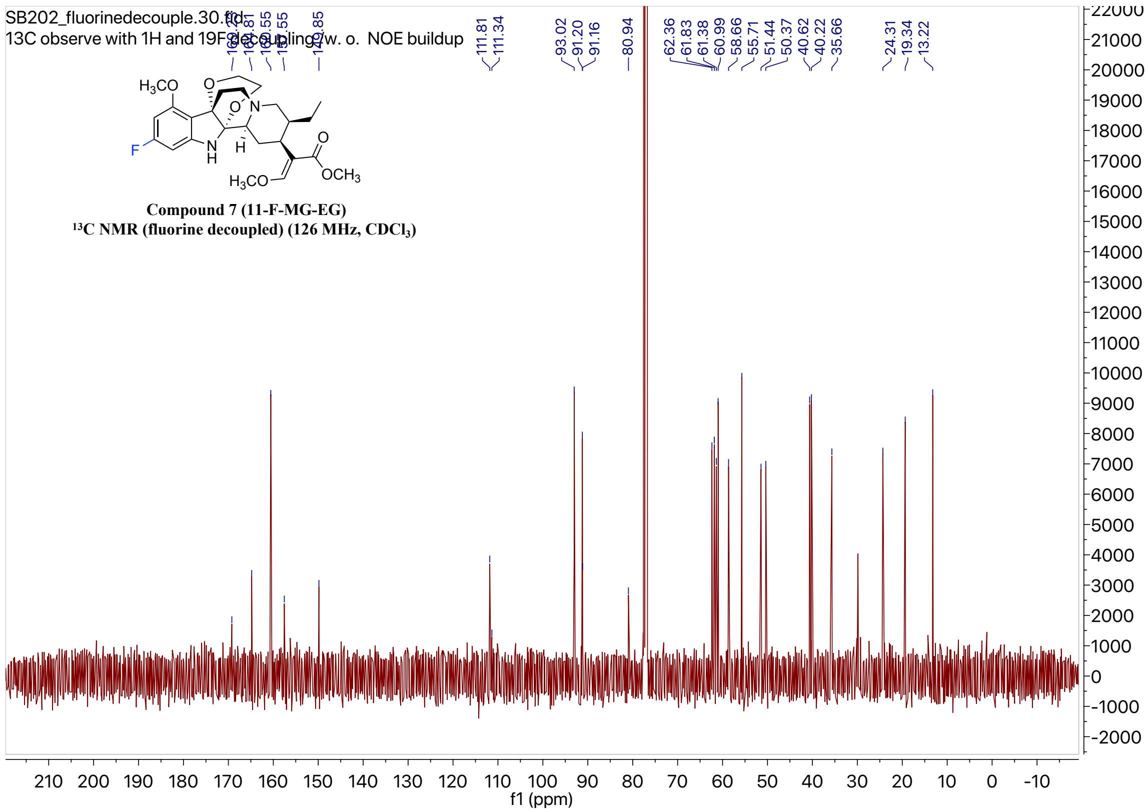

Supplementary Figure 23 : ^{19}F-NMR of 11-F-MG EG (**7**)

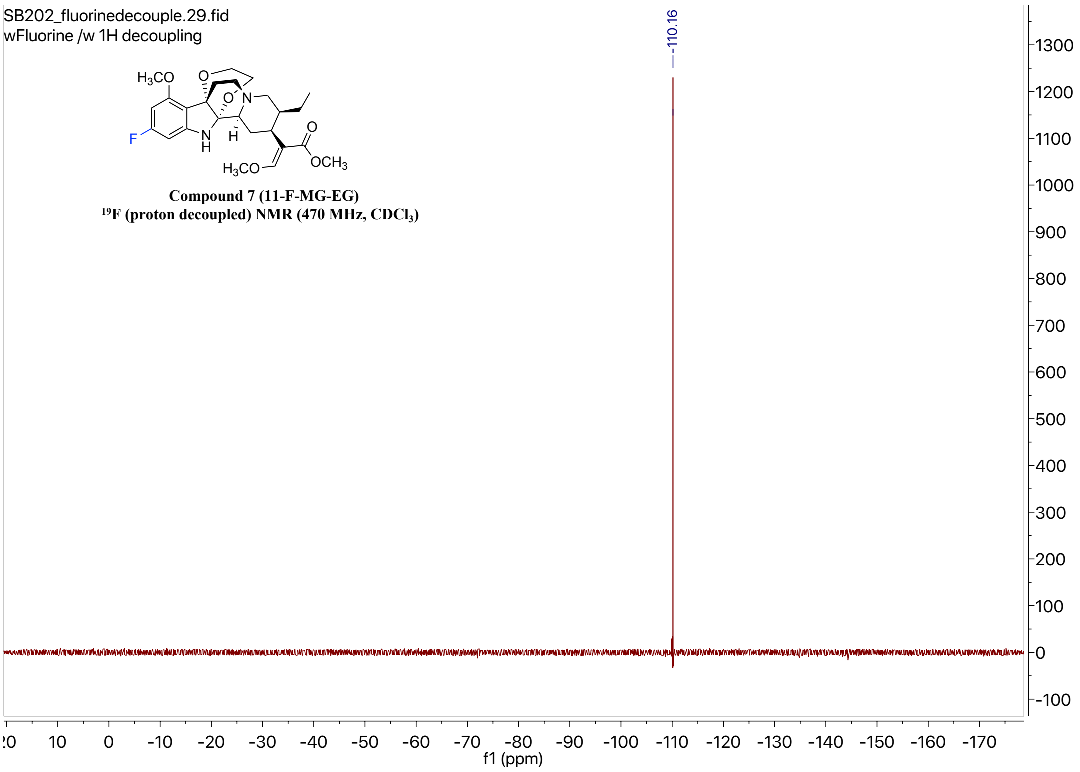

Supplementary Figure 24 : ^{1}H-NMR of 11-I-MG EG (**8**)

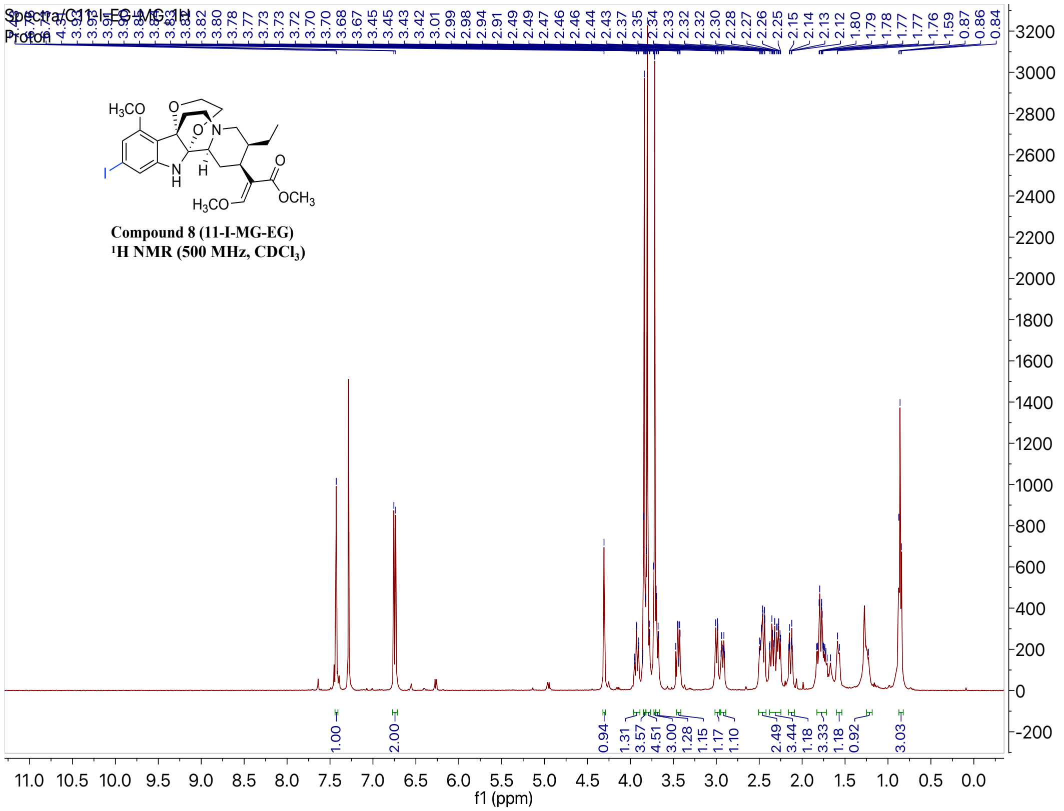

Supplementary Figure 25 : ^{13}C-NMR of 11-I-MG EG (**8**)

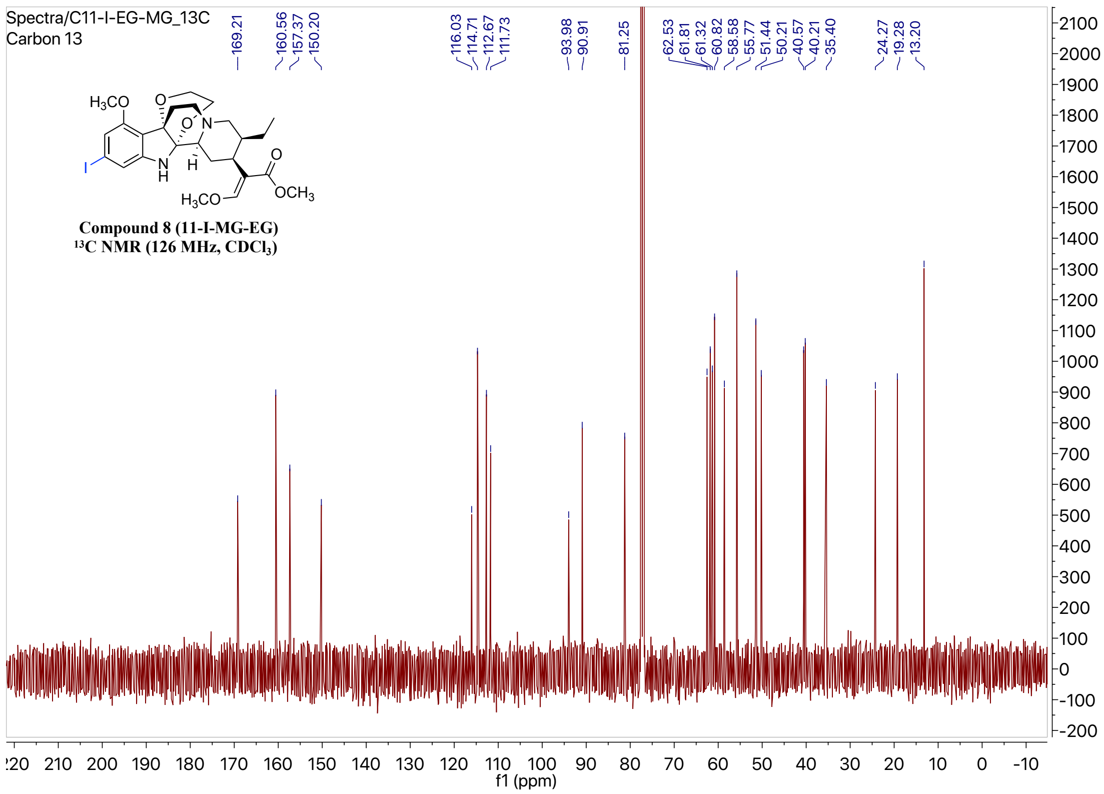

Supplementary Figure 26 : ^{1}H-NMR of 11-CH$_3$-MG EG (**9**)

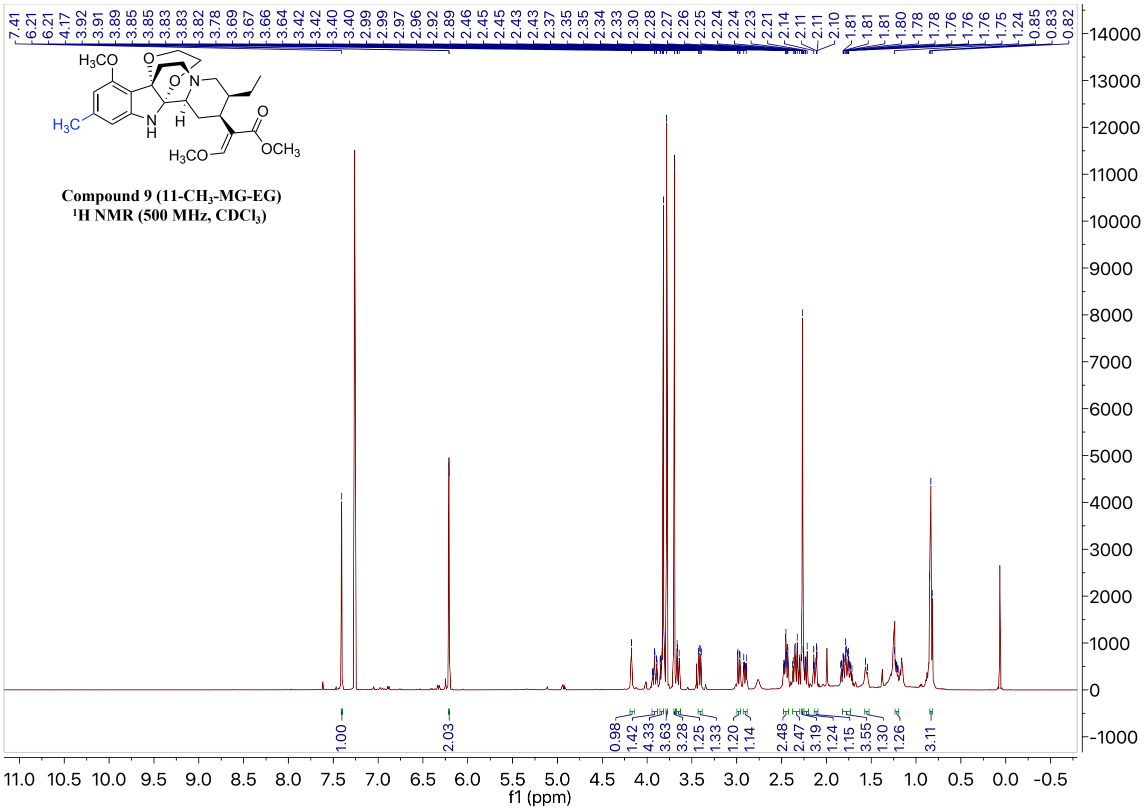

Supplementary Figure 27 : ^{13}C-NMR of 11-CH$_3$-MG EG (**9**)

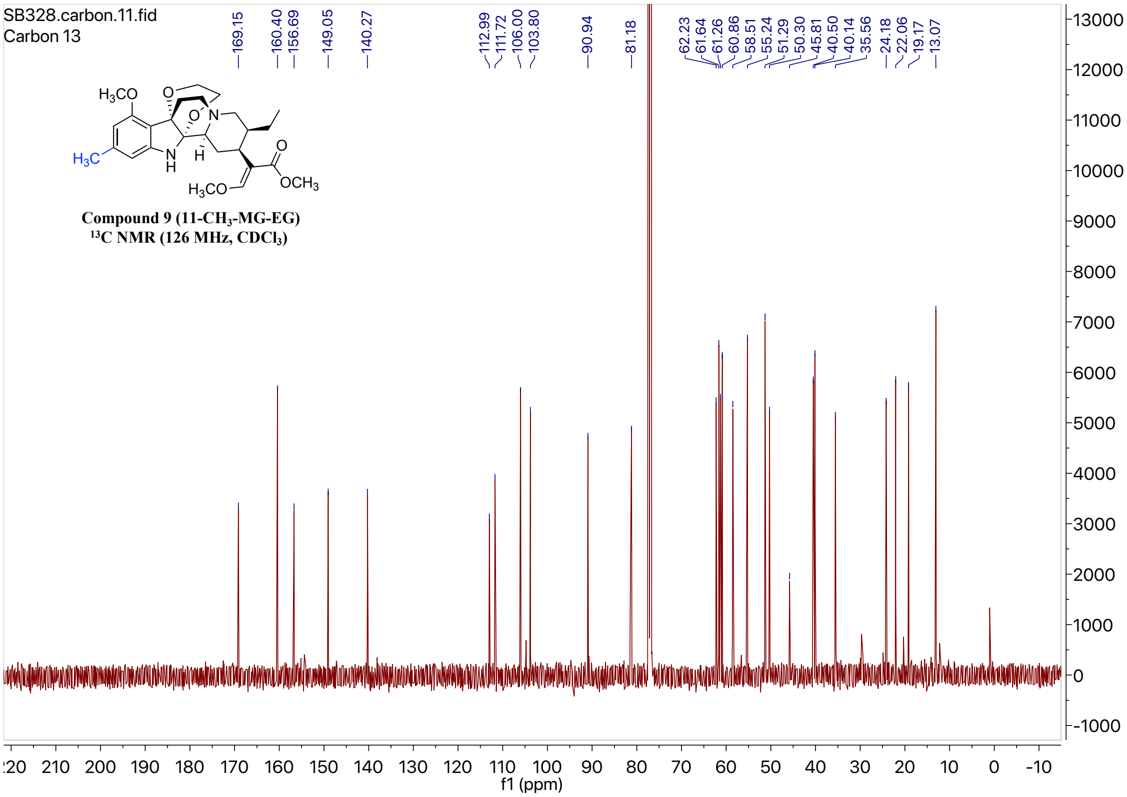

Supplementary Figure 28 : ^{1}H-NMR of 11-Ph-MG EG (**10**)

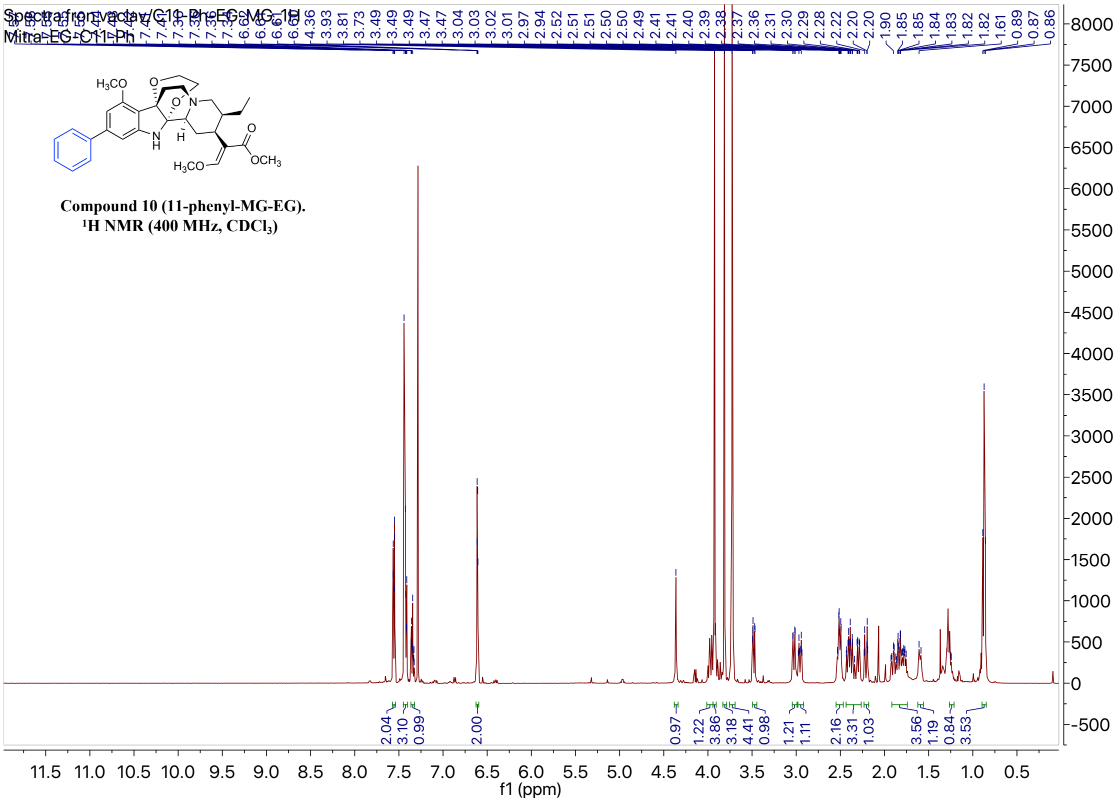

Supplementary Figure 29 : ^{13}C-NMR of 11-Ph-MG EG (**10**)

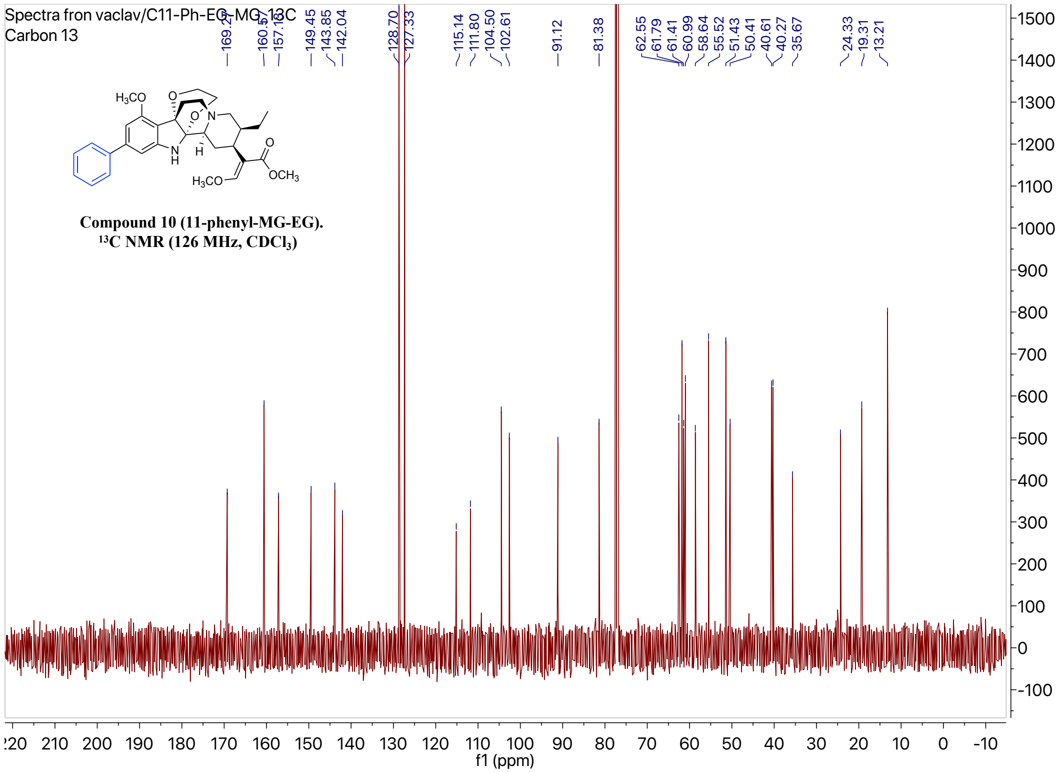

Supplementary Figure 30 : ^{1}H-NMR of 11-CONH$_2$-MG EG (**11**)

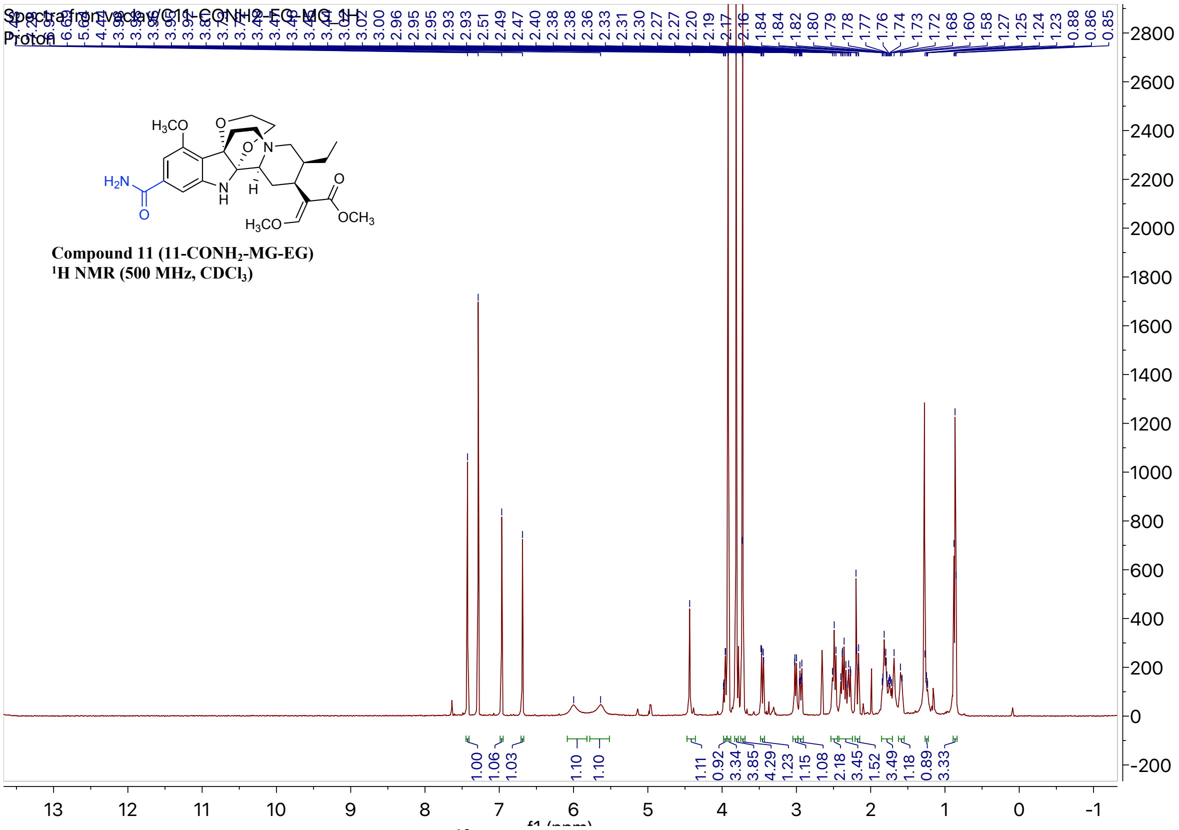

Supplementary Figure 31 : ^{13}C-NMR of 11-CONH$_2$-MG EG (**11**)

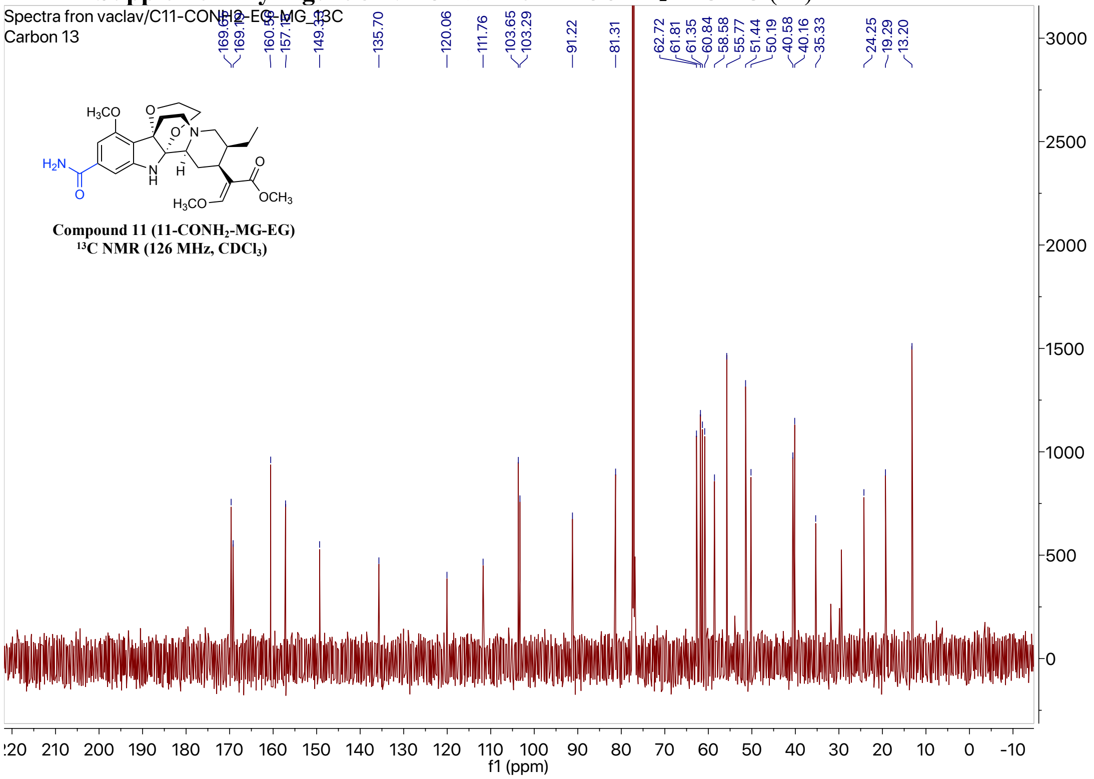

Supplementary Figure 32 : ^{1}H-NMR of 11-CN-MG EG (**12**)

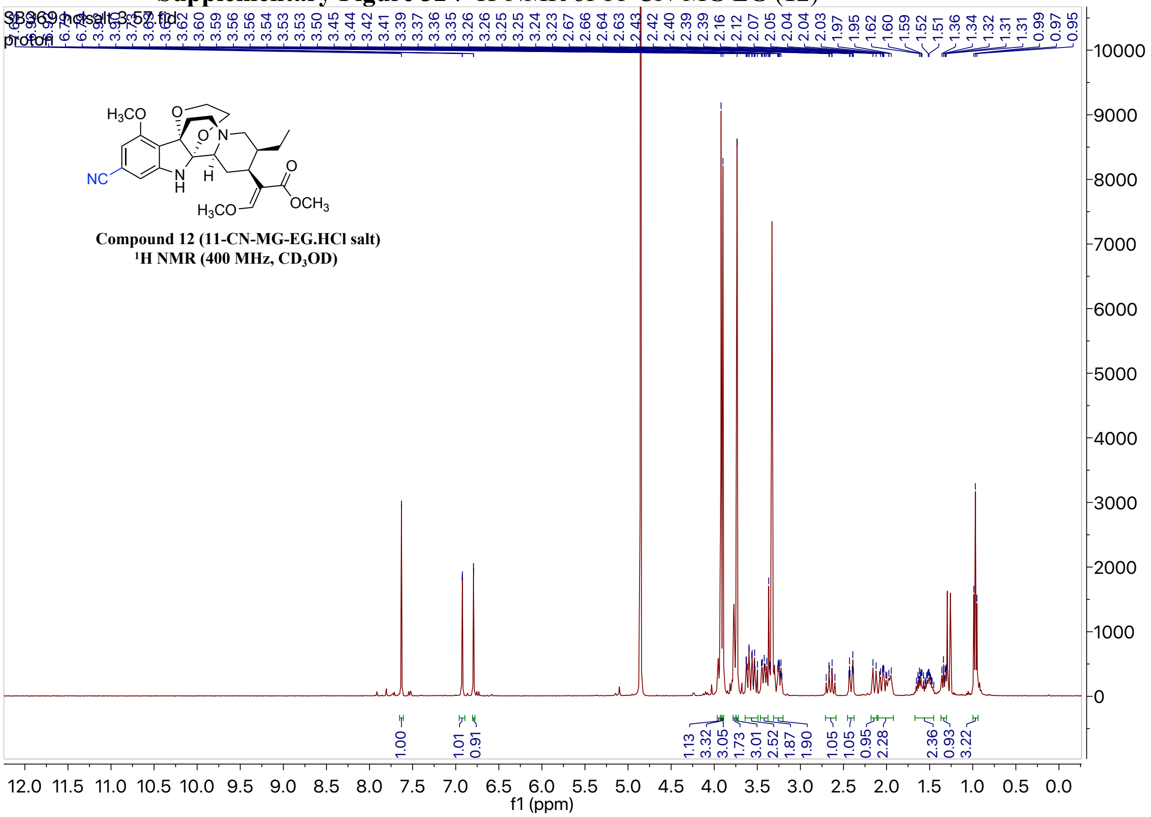

Supplementary Figure 33 : ^{13}C-NMR of 11-CN-MG EG (**12**)

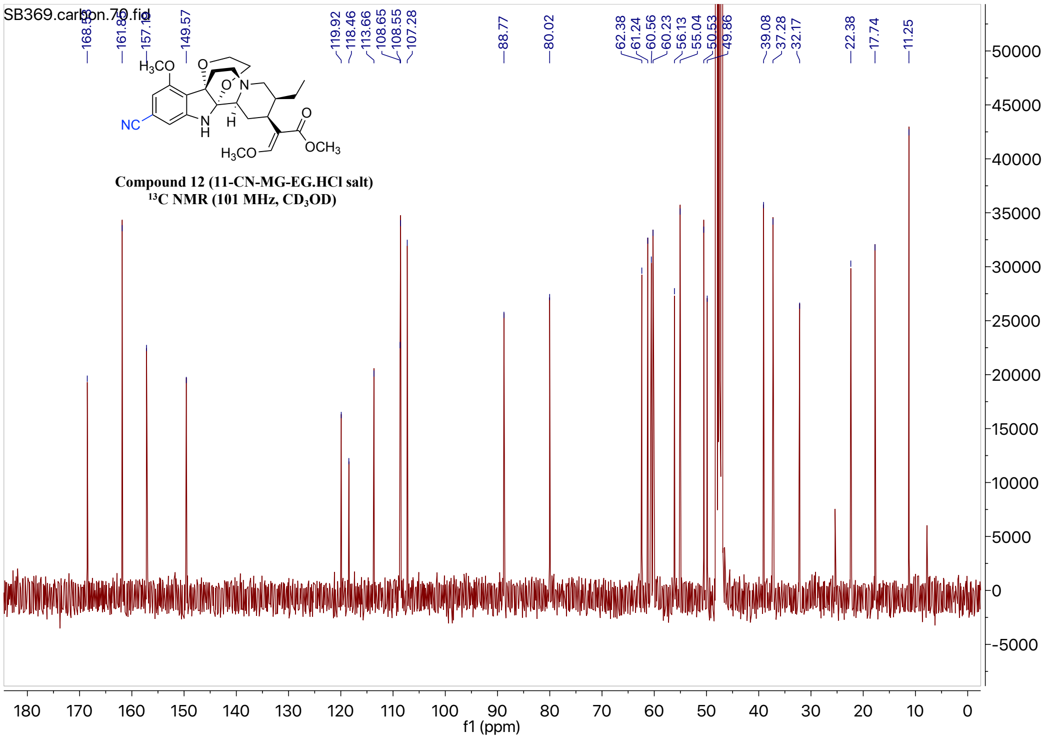

Supplementary Figure 34 : ^{1}H-NMR of 11-Cl-MG EG (**13**)

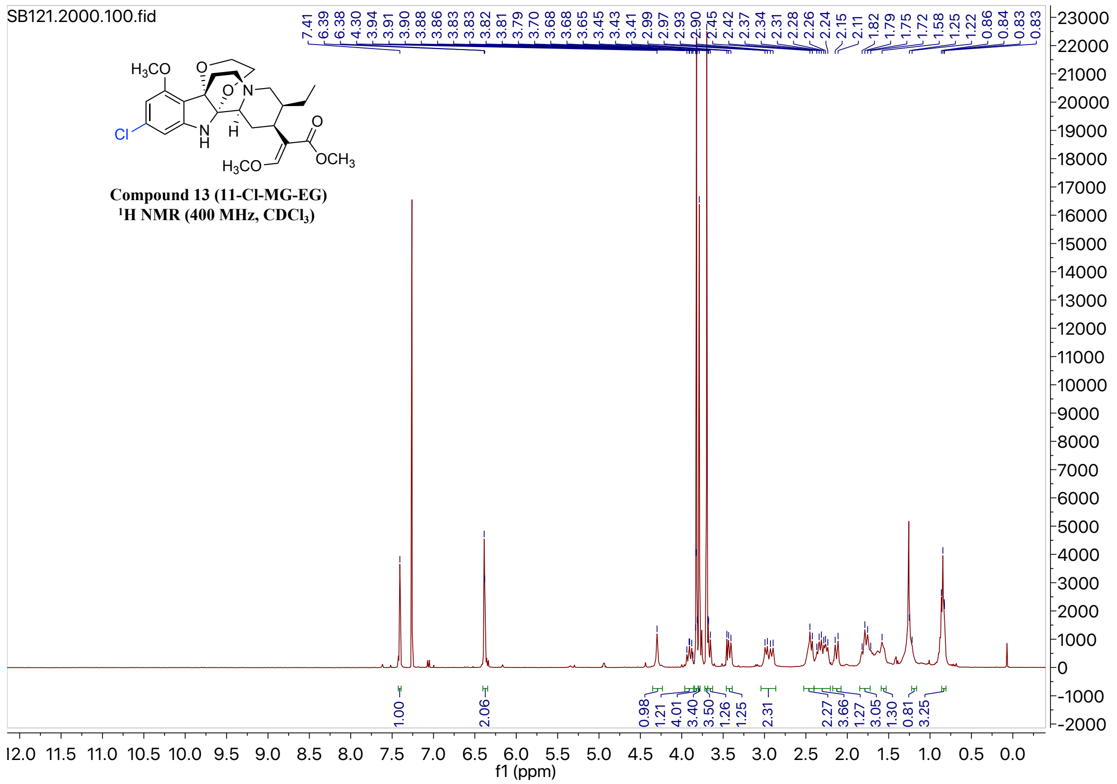

Supplementary Figure 35 : ^{13}C-NMR of 11-Cl-MG EG (**13**)

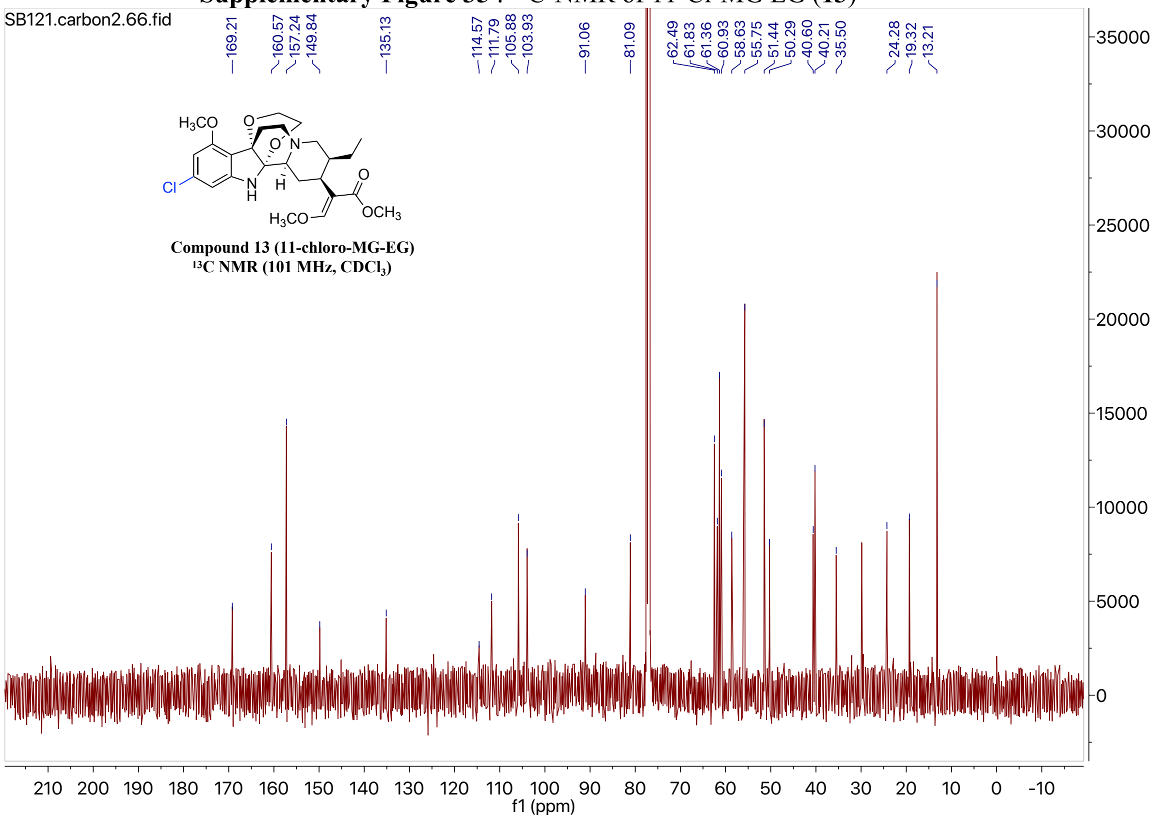

Supplementary Figure 36 : ^{1}H-NMR of 11-OH-MG EG (**14**)

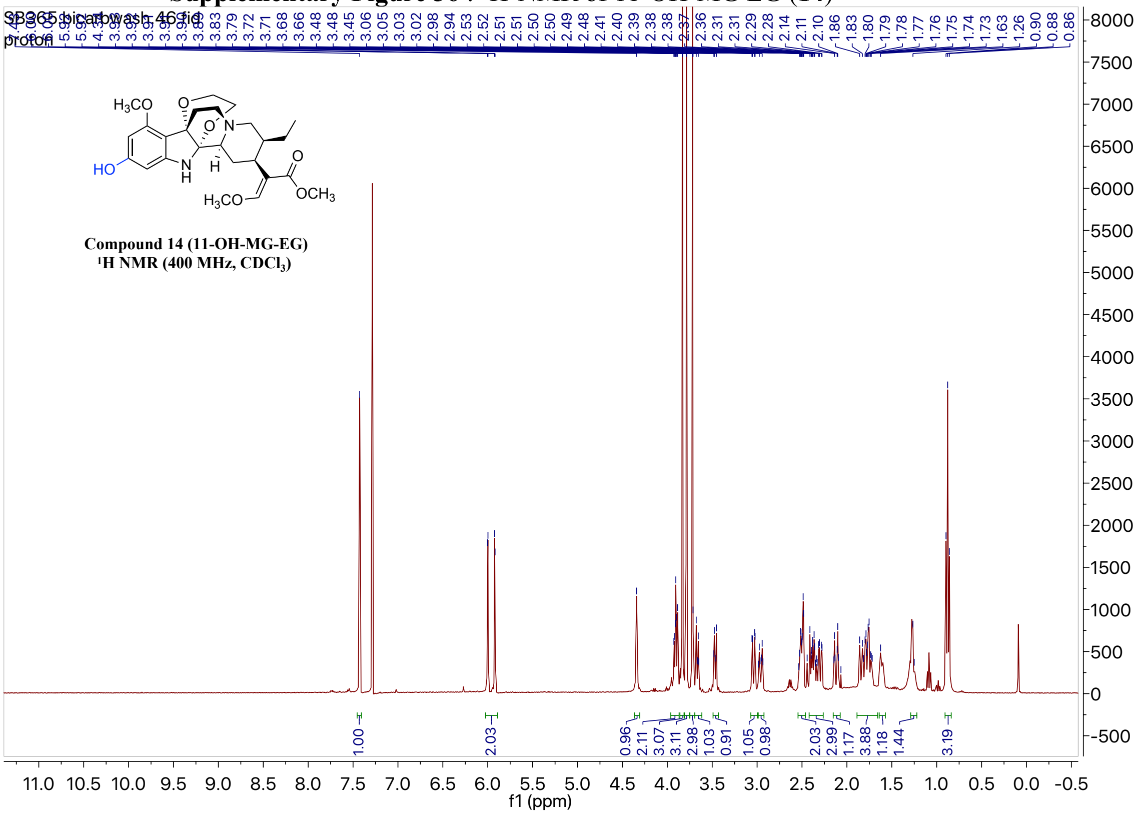

Supplementary Figure 37 : ^{13}C-NMR of 11-OH-MG EG (**14**)

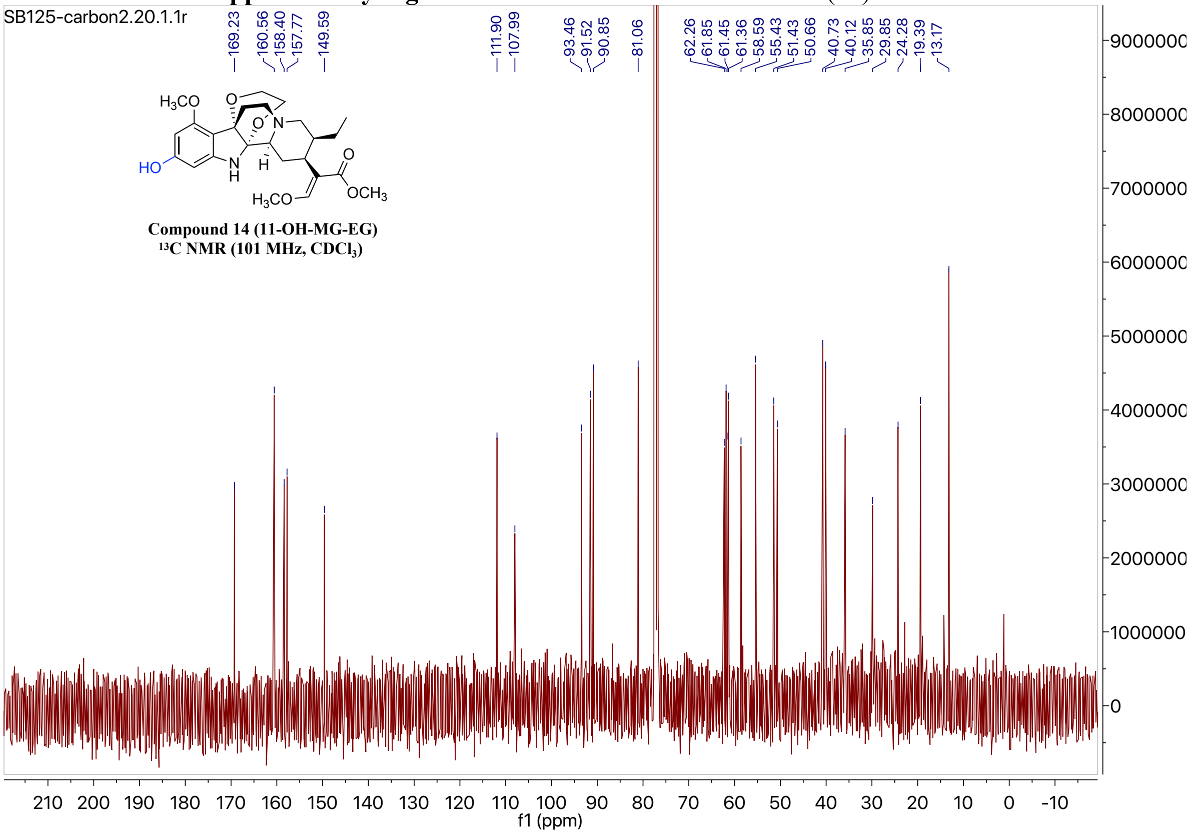

Supplementary Figure 38 : ^{1}H-NMR of 11-OTf-MG EG (**14a**)

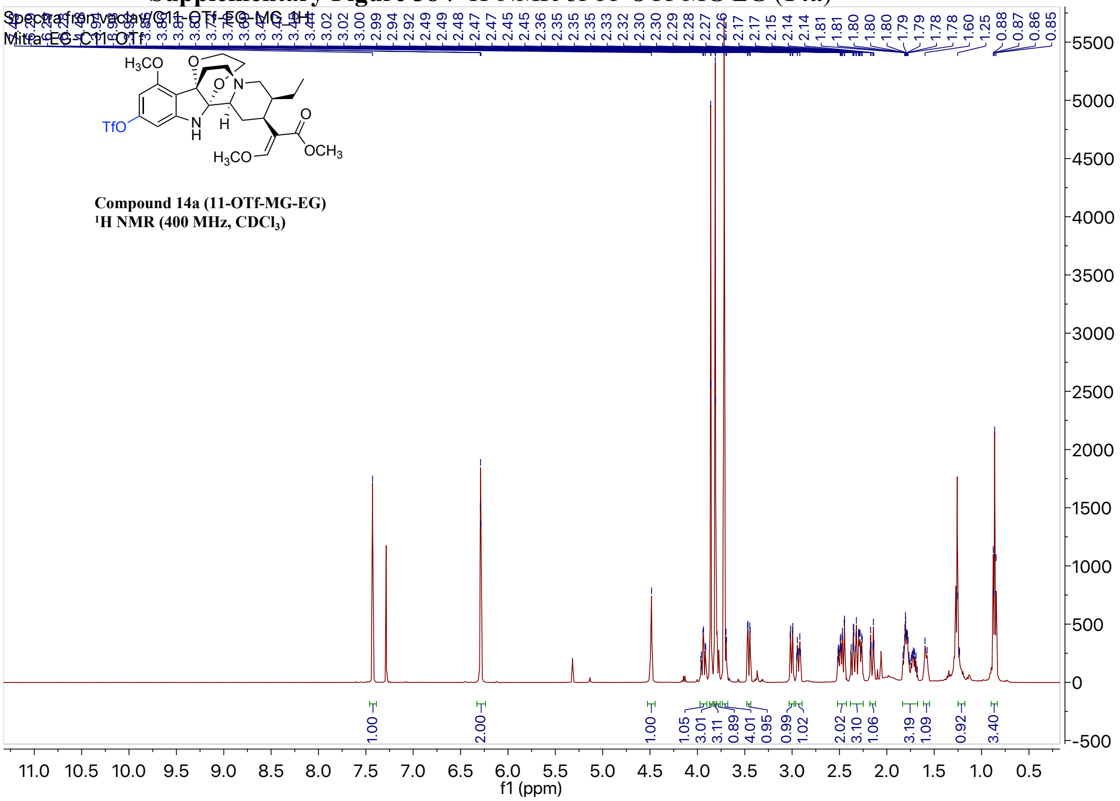

Supplementary Figure 39 : ^{13}C-NMR of 11-OTf-MG EG (**14a**)

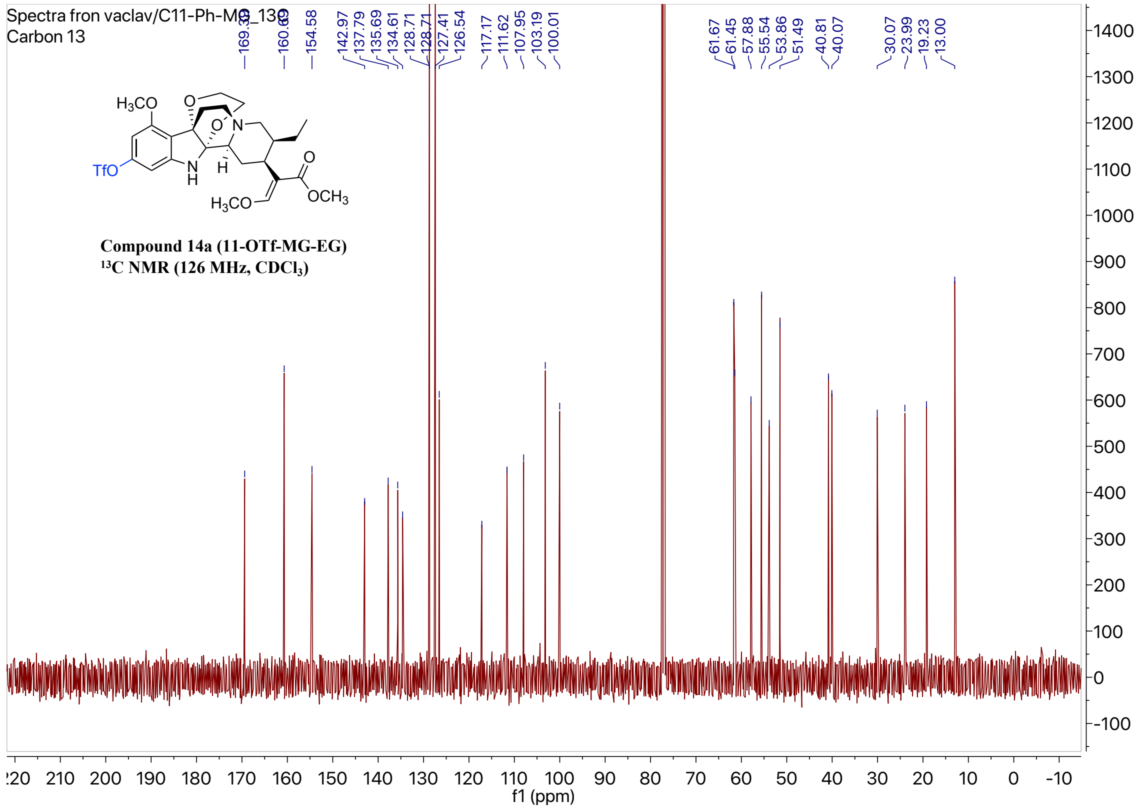

Supplementary Figure 40 : ^{19}F-NMR of 11-OTf-MG EG (**14a**)

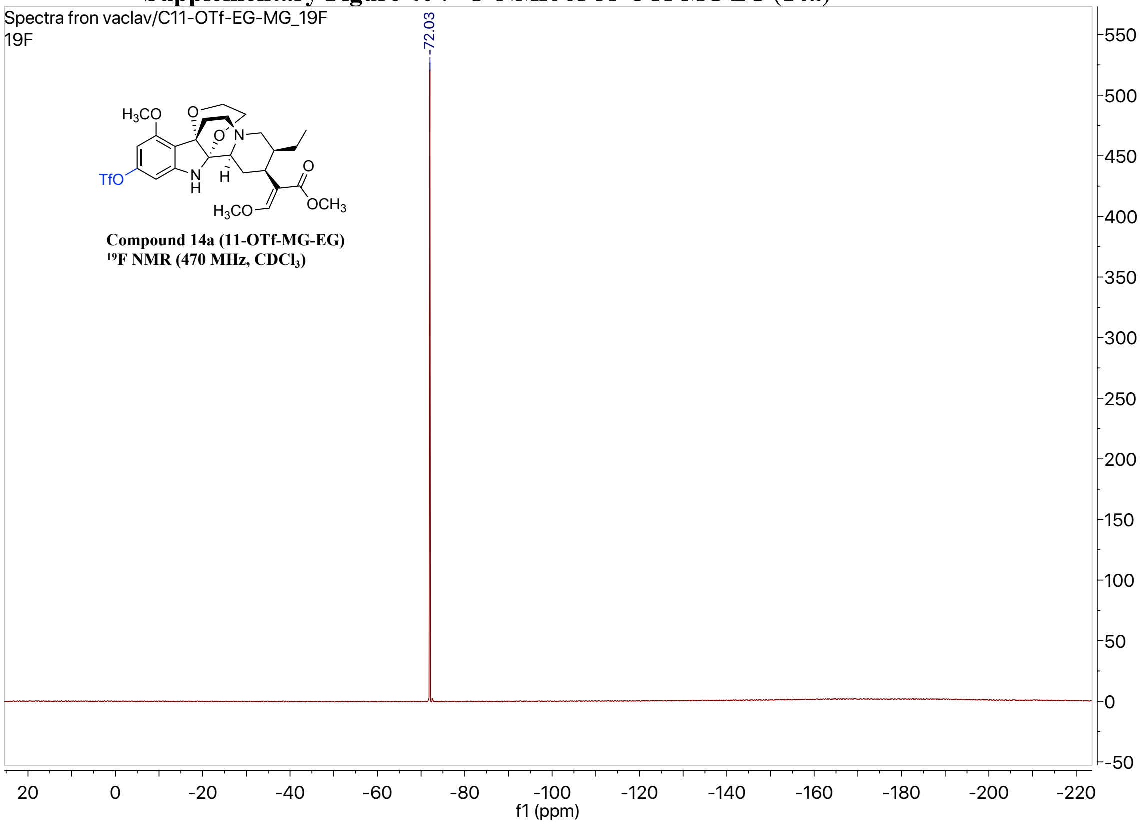

Supplementary Figure 41 : ^{1}H-NMR of 11-SnBu$_3$-MG EG (**14b**)

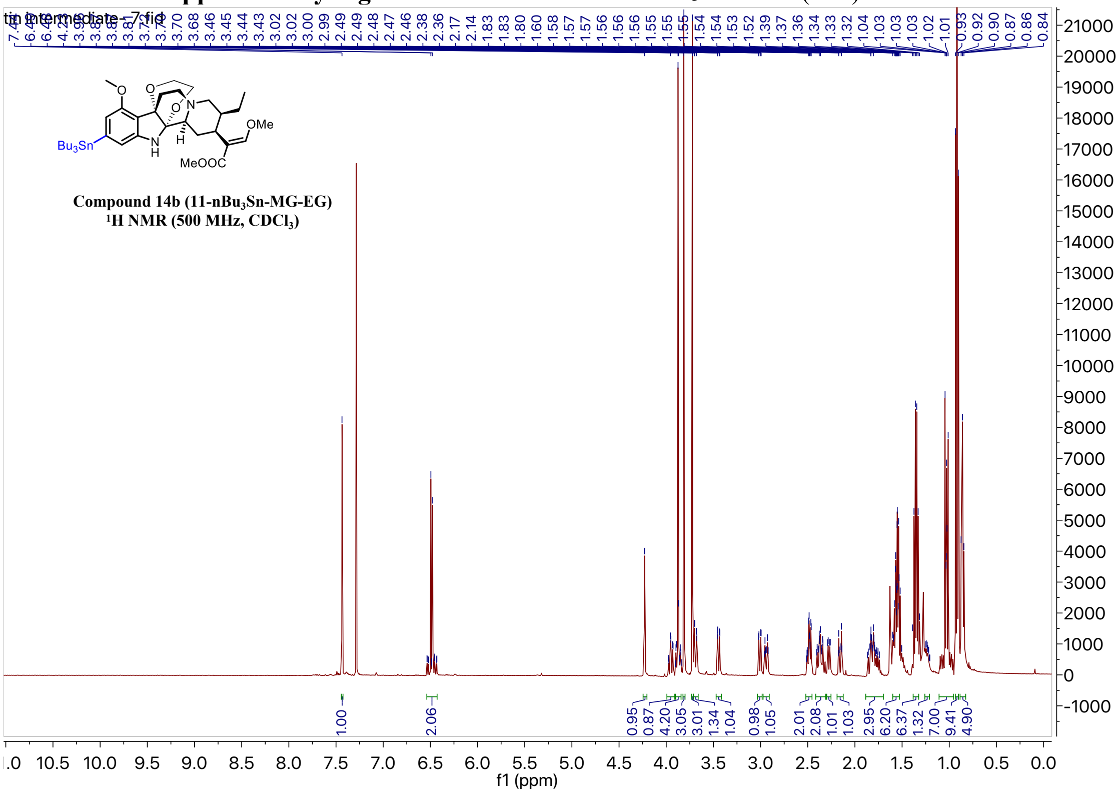

Supplementary Figure 42 : ^{13}C-NMR of 11-SnBu3-MG EG (**14b**)

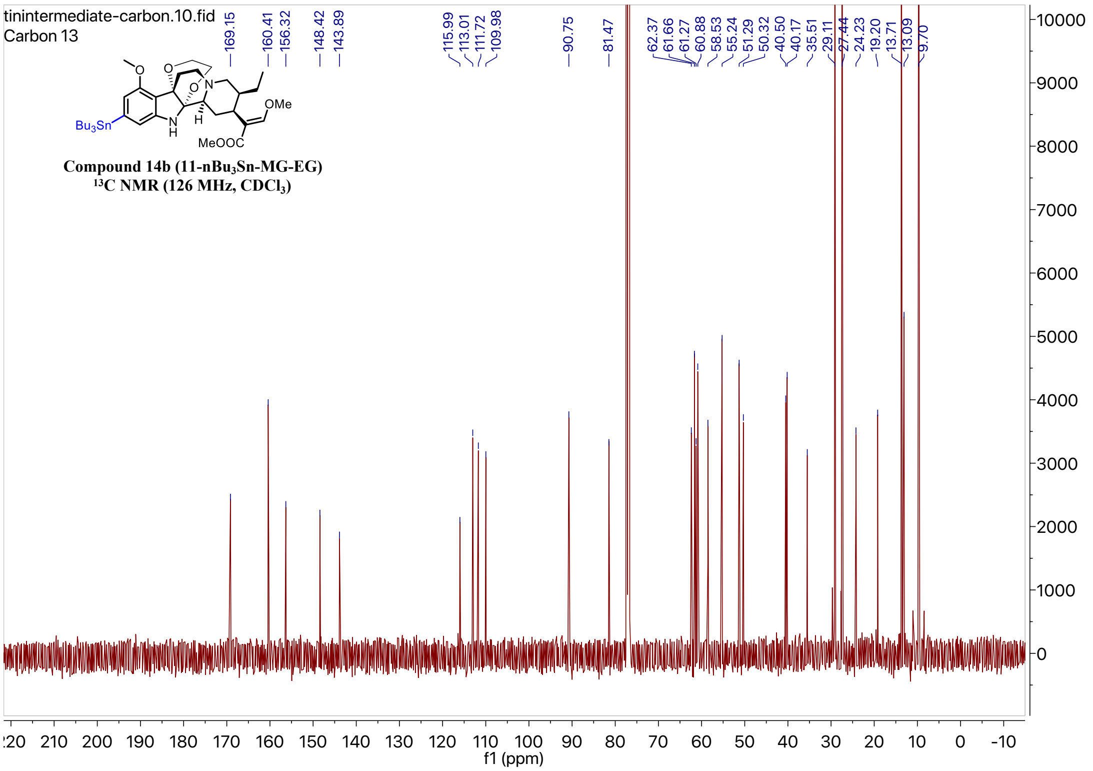

Supplementary Figure 43 : ^{1}H-NMR of 11-F-MG (**15**)

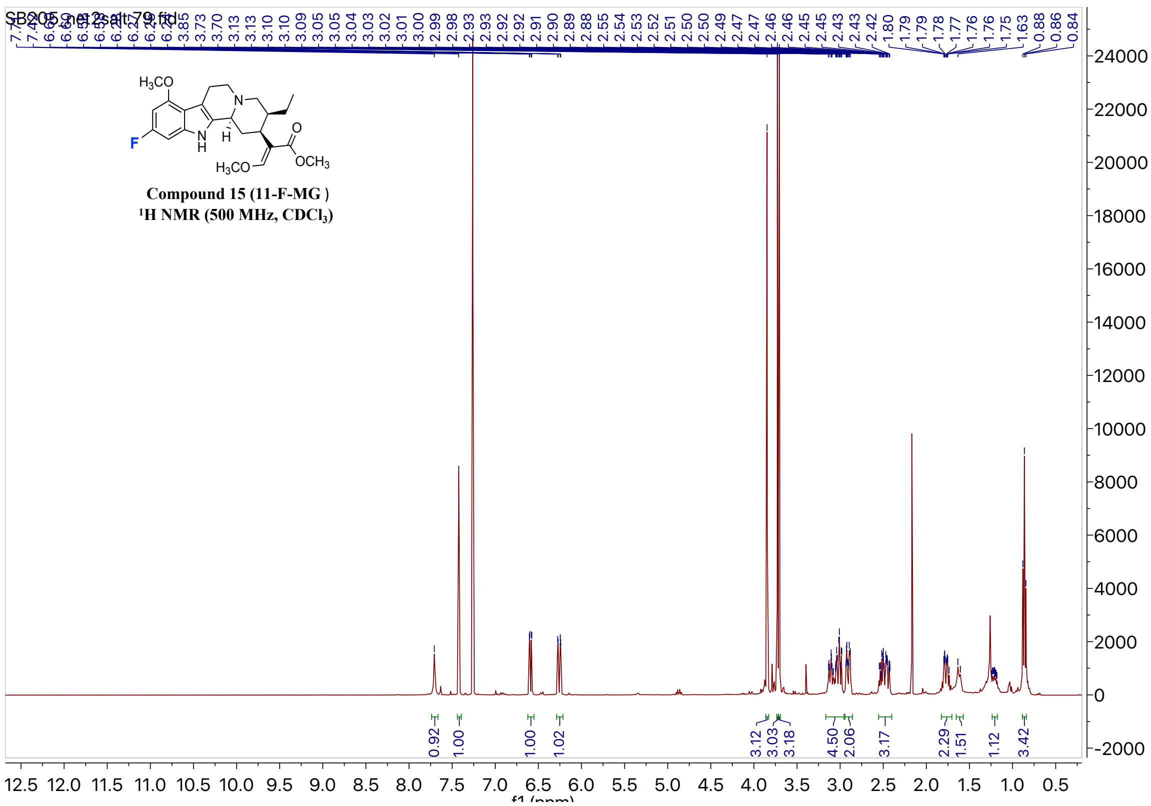

Supplementary Figure 44 : ^{13}C-NMR of 11-F-MG (**15**)

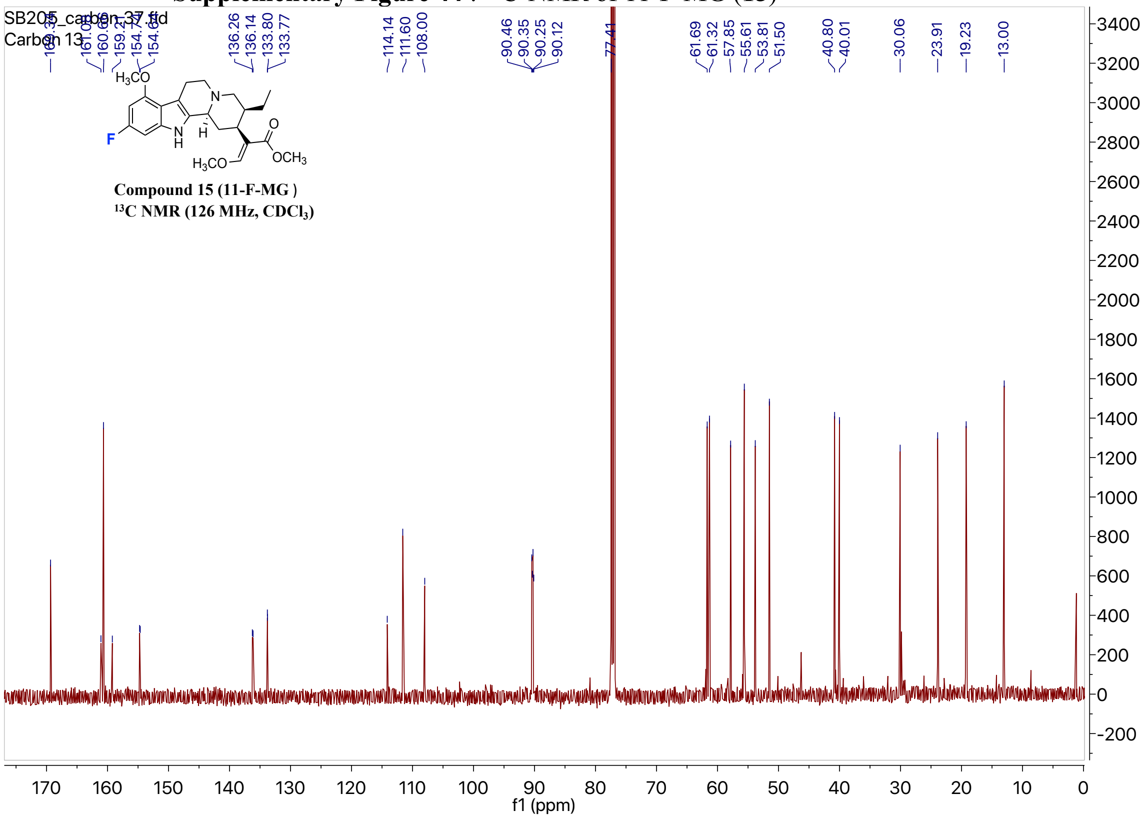

Supplementary Figure 45 : ^{19}F-NMR of 11-F-MG (**15**)

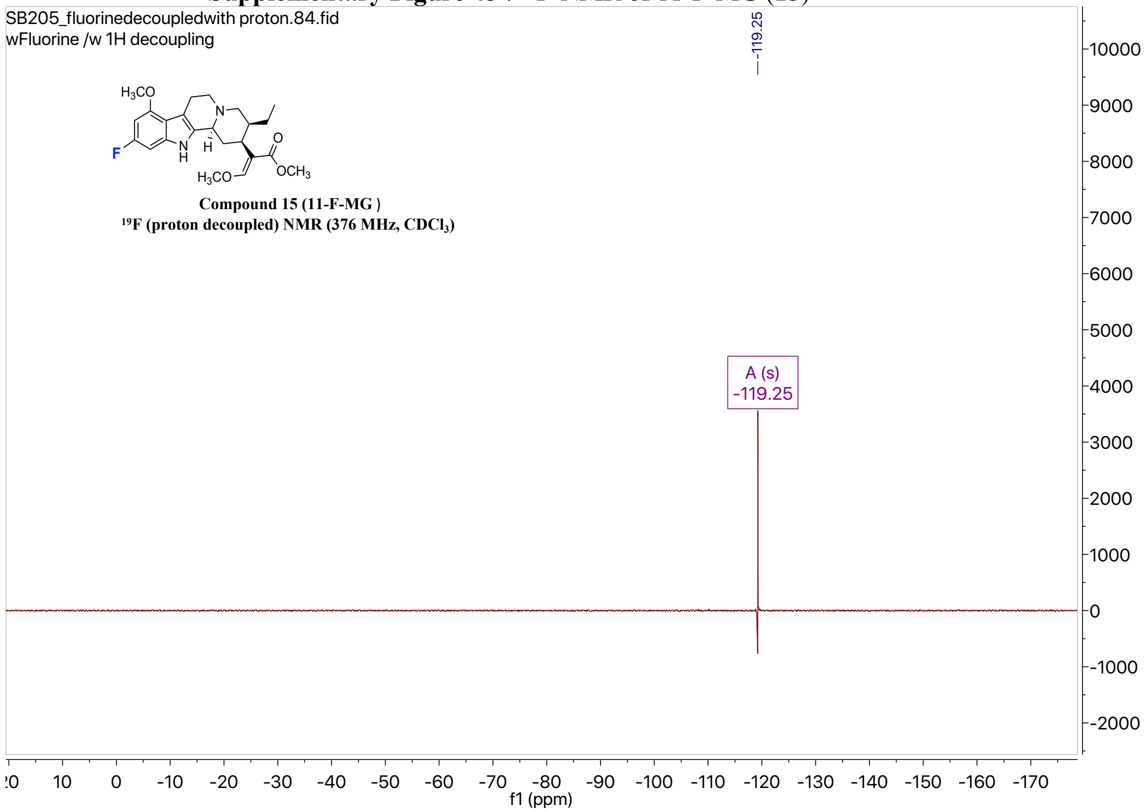

Supplementary Figure 46 : ^{1}H-NMR of 11-Cl-MG (**16**)

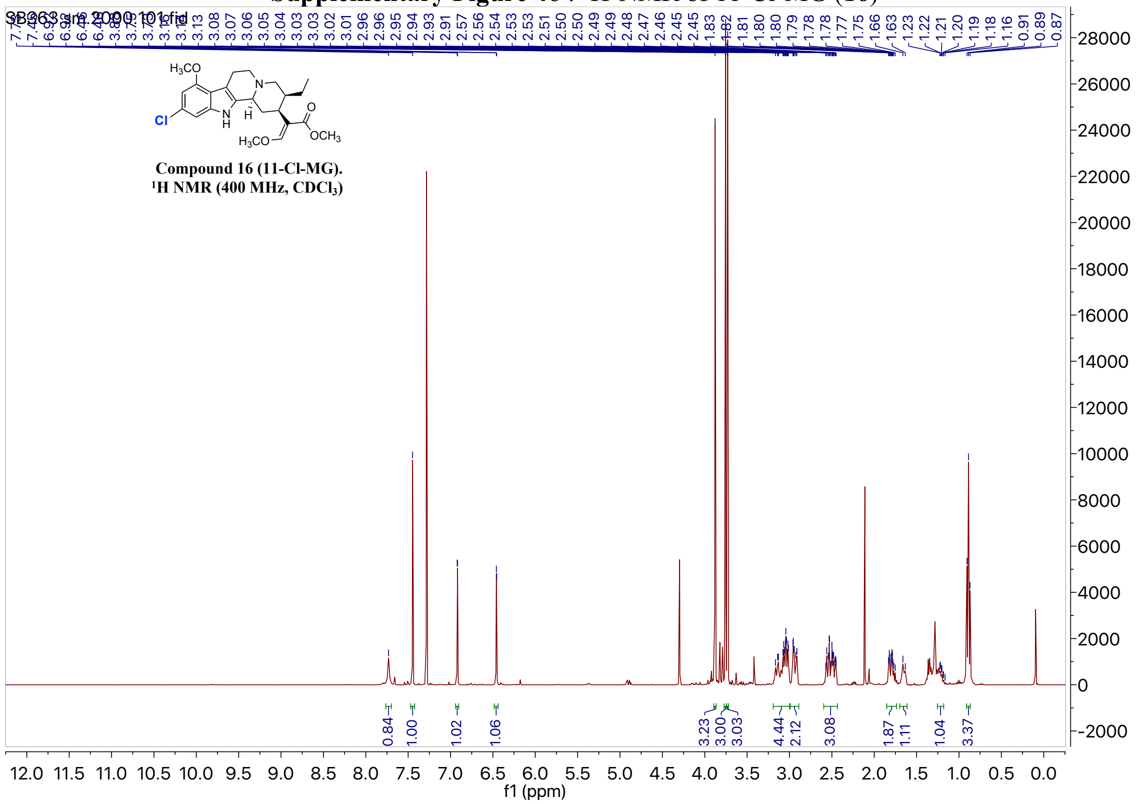

Supplementary Figure 47 : ^{13}C-NMR of 11-Cl-MG (**16**)

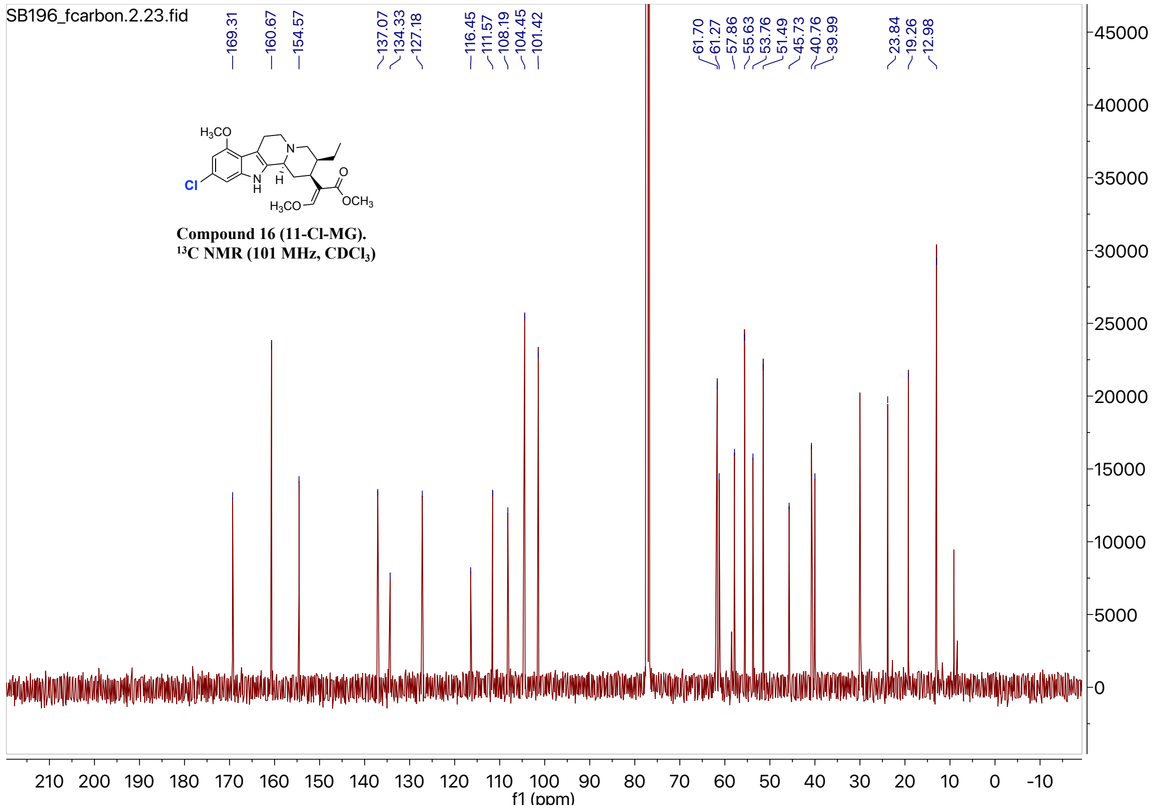

Supplementary Figure 48 : ^{1}H-NMR of 11-Br-MG (**17**)

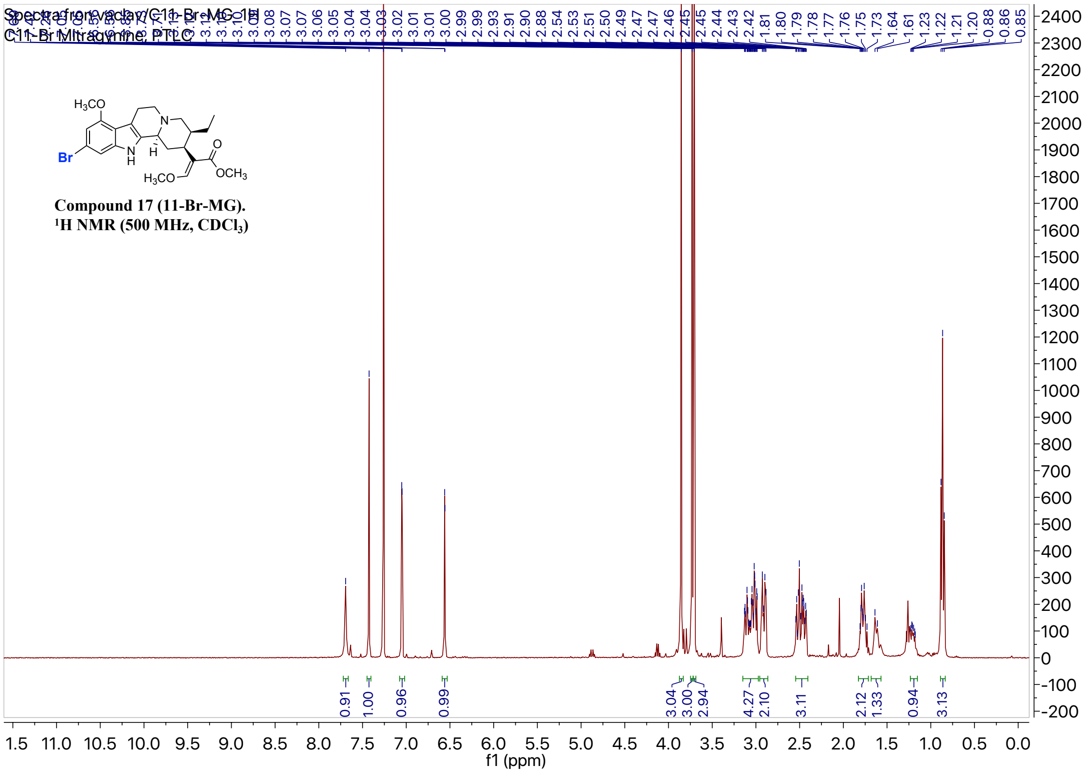

Supplementary Figure 49 : ^{13}C-NMR of 11-Br-MG (**17**)

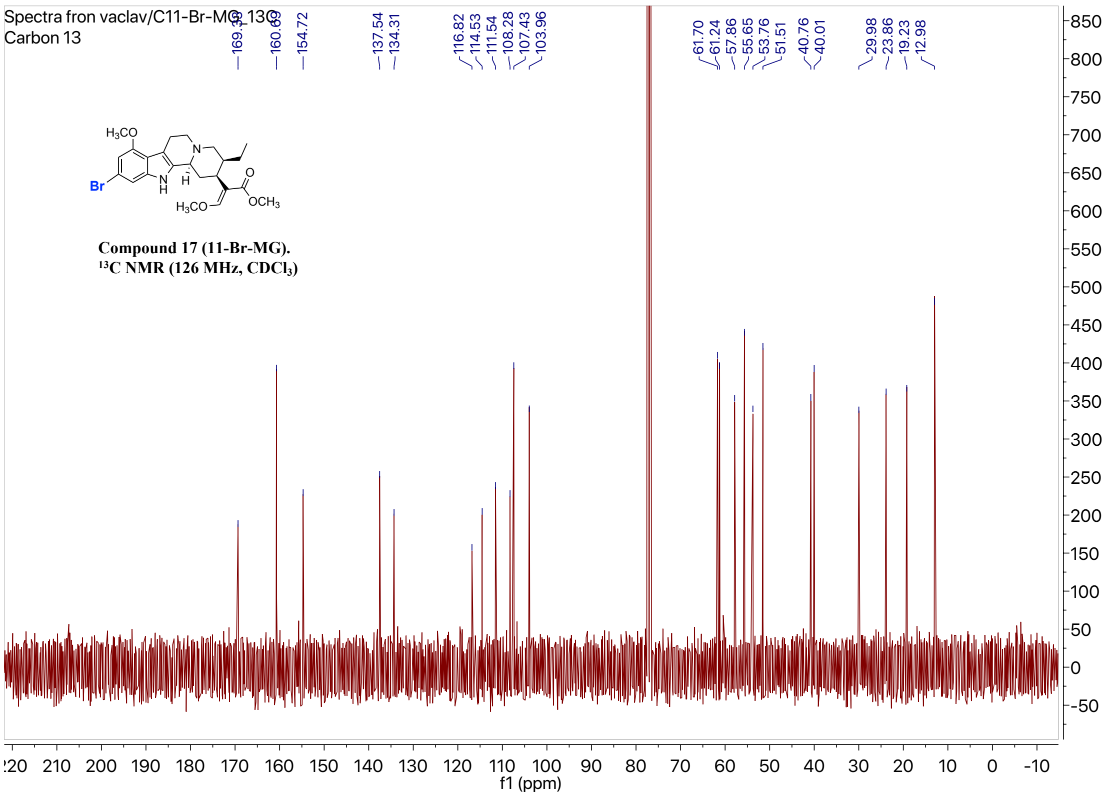

Supplementary Figure 50 : ^{1}H-NMR of 11-CH3-MG (**18**)

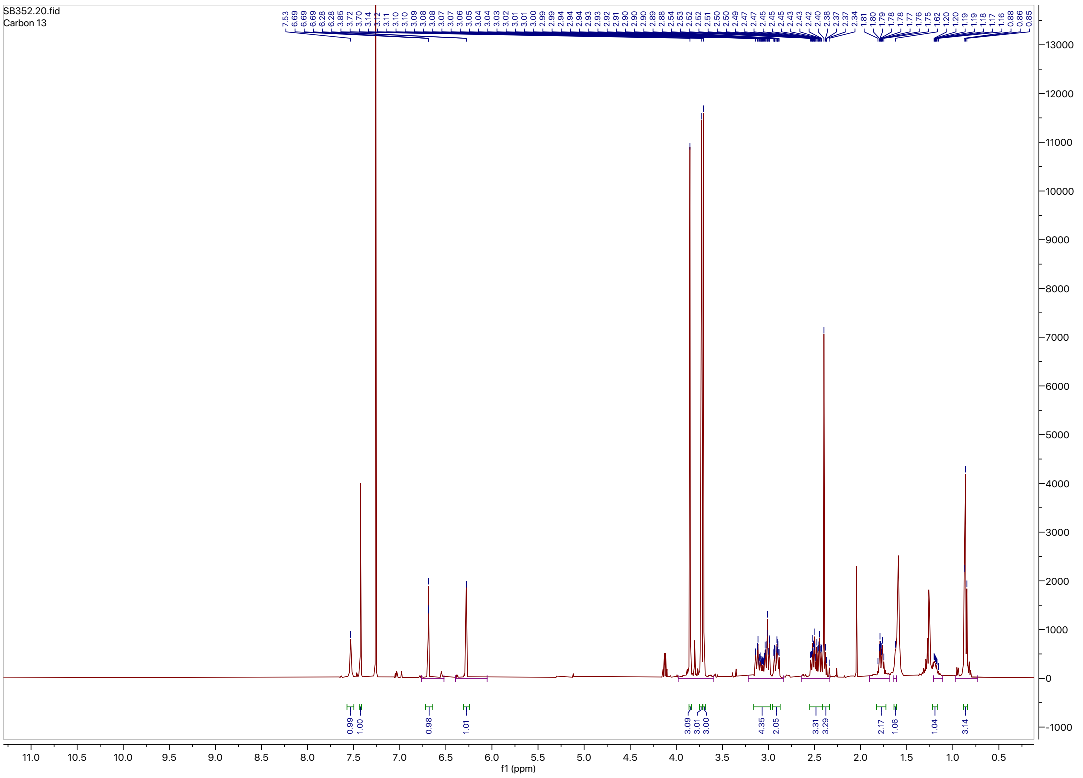

Supplementary Figure 51 : ^{13}C-NMR of 11-CH3-MG (**18**)

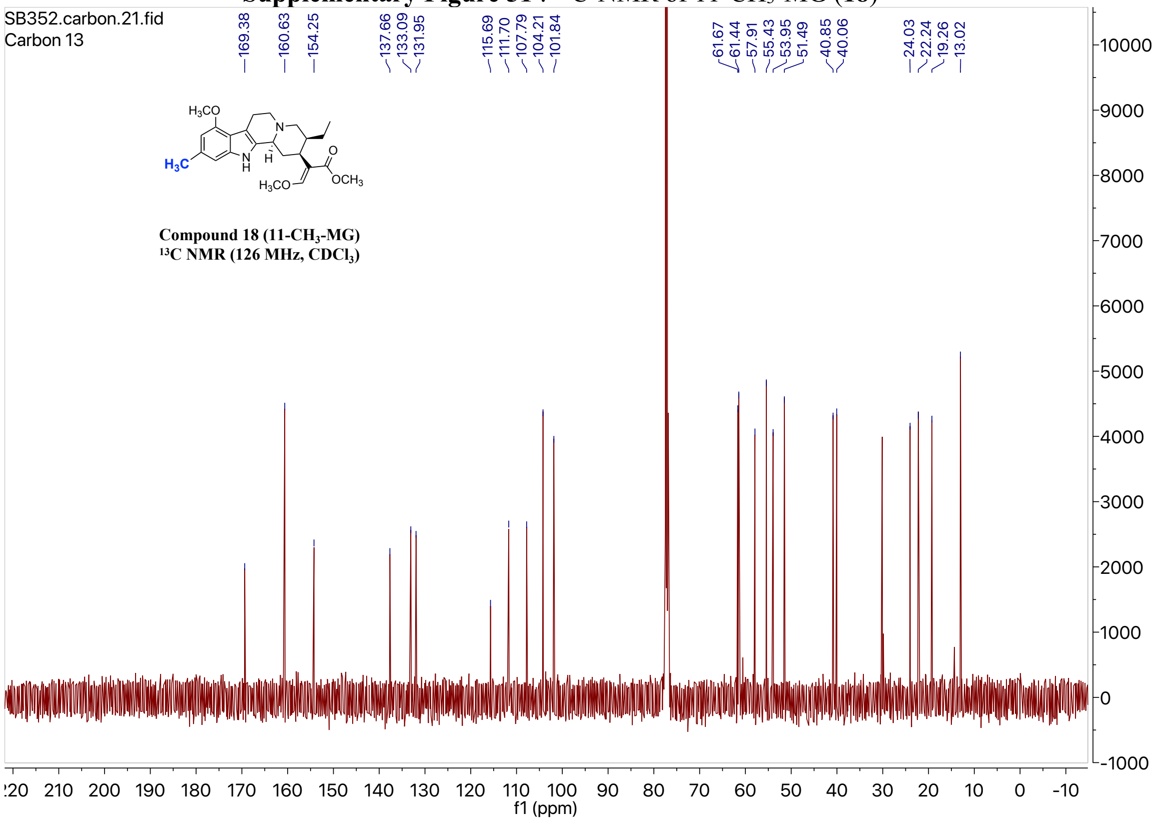

Supplementary Figure 52 : ^{1}H-NMR of 11-CH_3-MG (**19**)

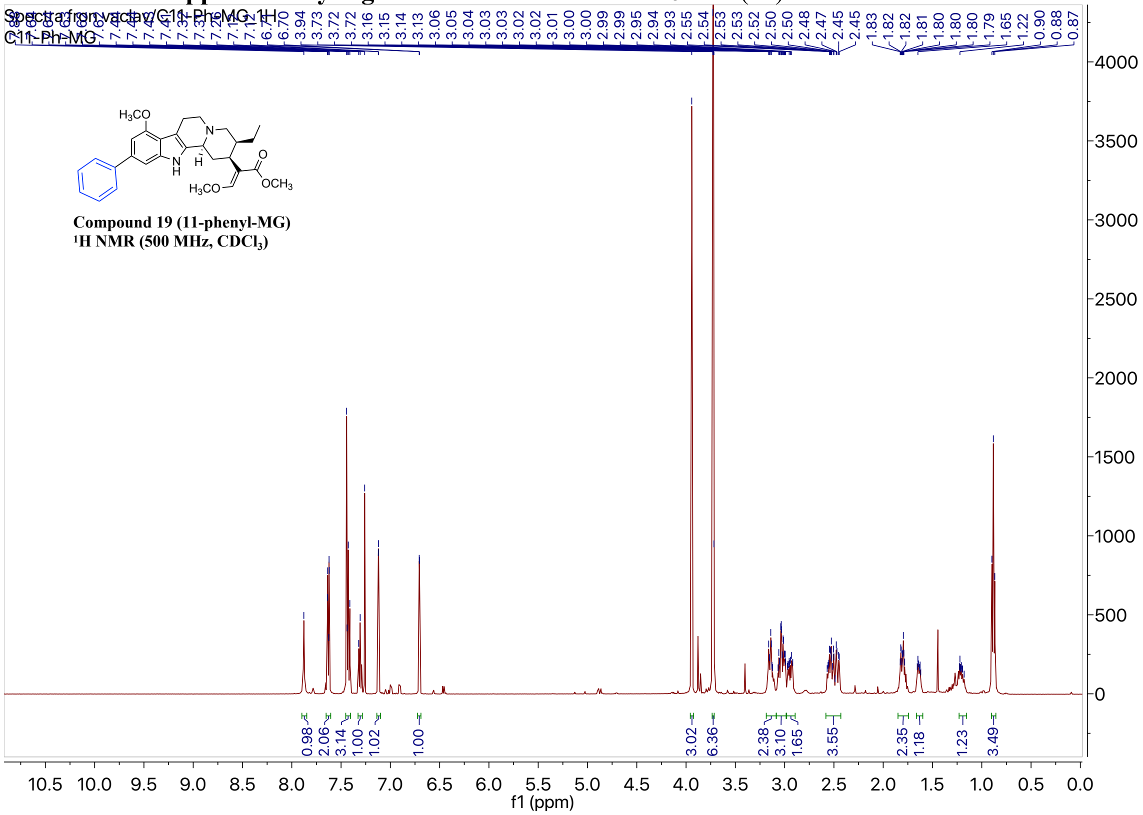

Supplementary Figure 53 : ^{13}C-NMR of 11-Ph-MG EG (**19**)

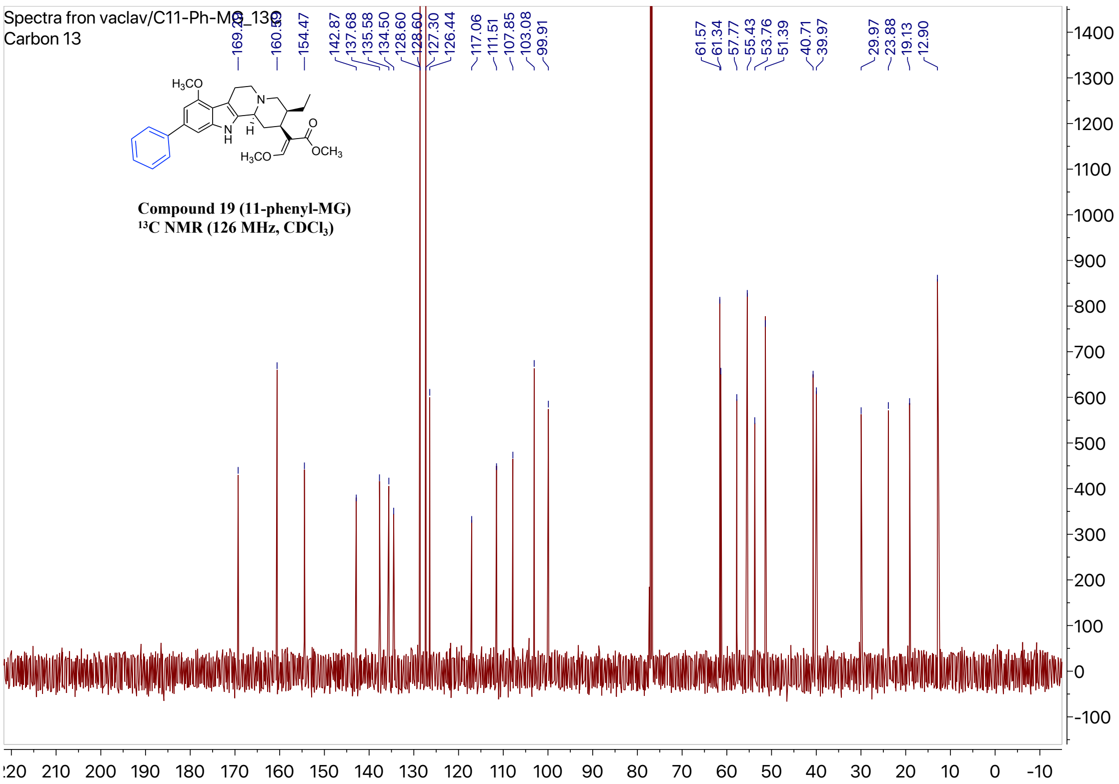

Supplementary Figure 54 : ^{1}H-NMR of 11-CONH$_2$-MG (**20**)

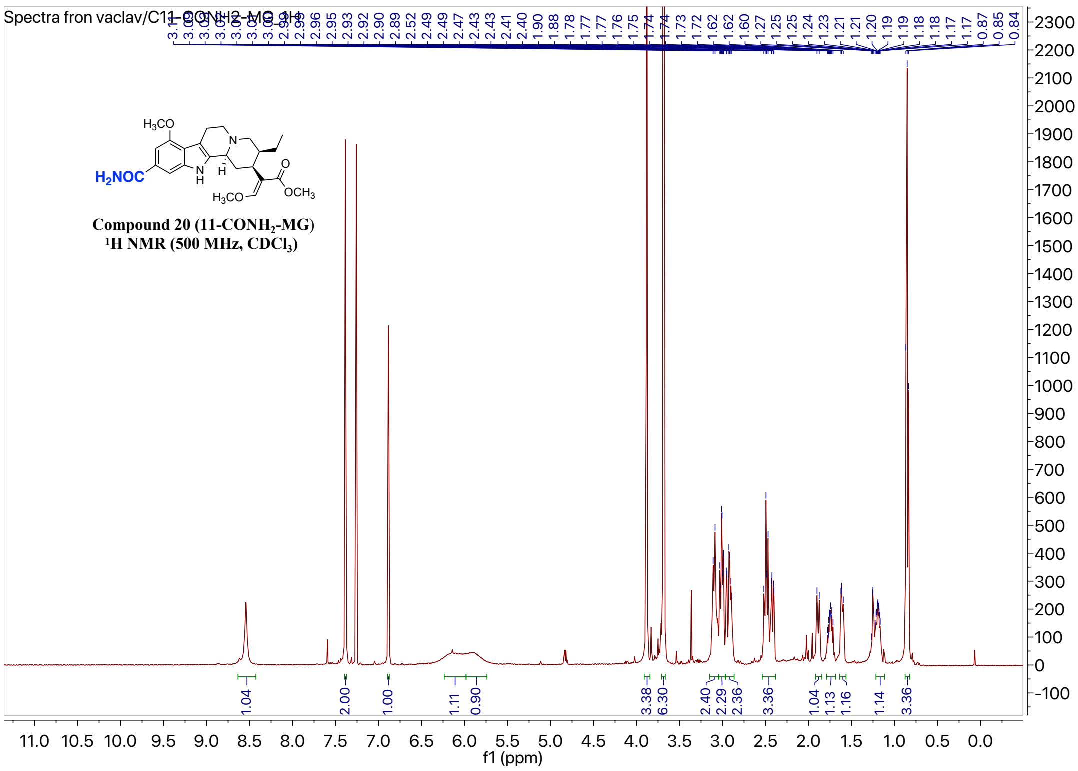

Supplementary Figure 55 : ^{13}C-NMR of 11-CONH$_2$-MG (**19**)

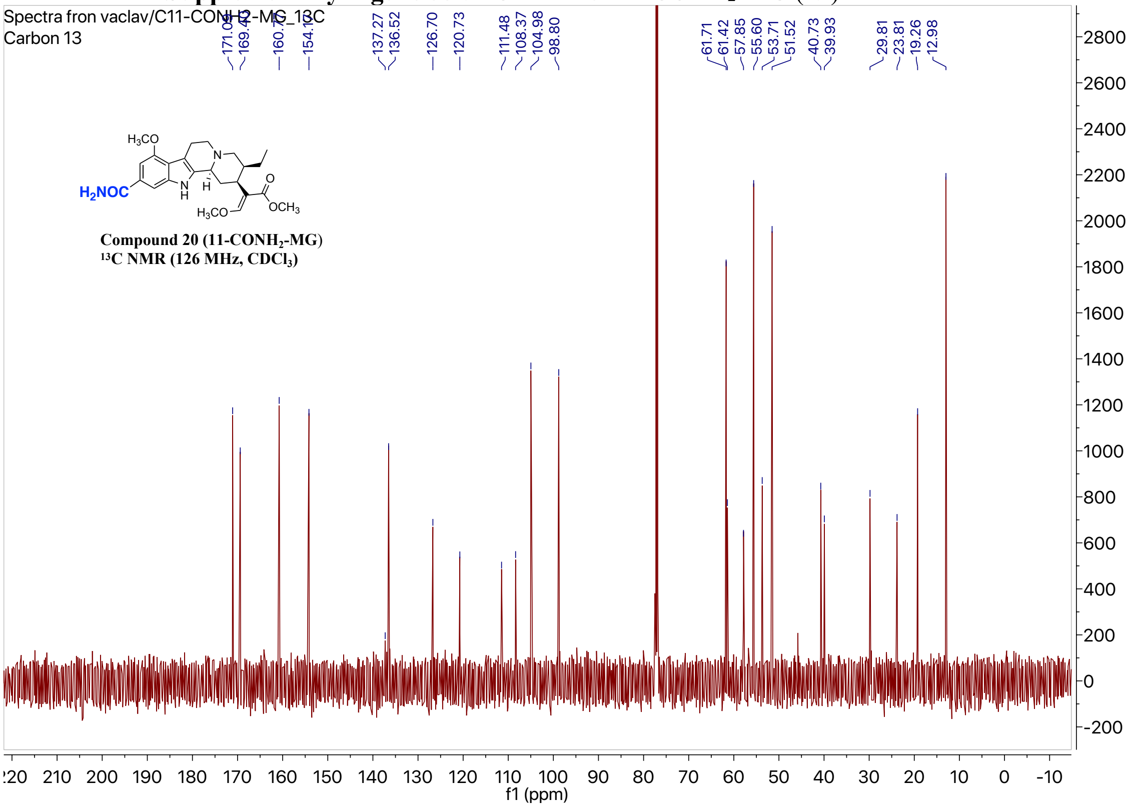

Supplementary Figure 56 : ^{1}H-NMR of 11-CN-MG (**21**)

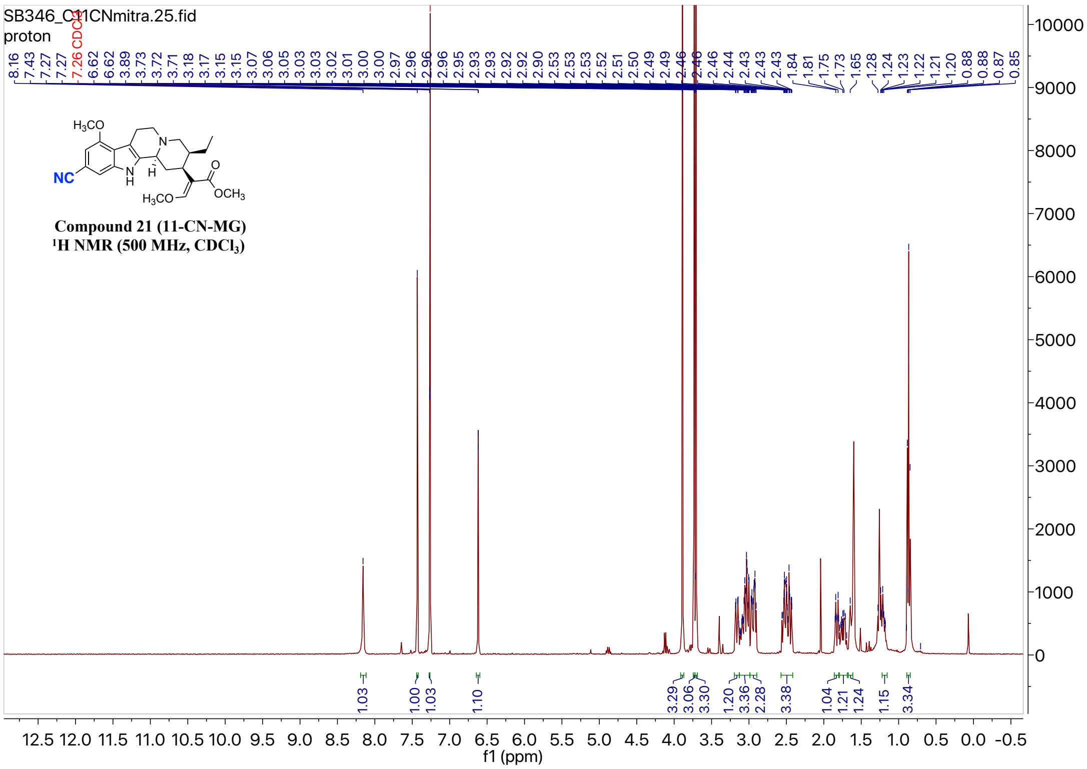

Supplementary Figure 57 : ^{13}C-NMR of 11-CN-MG (**21**)

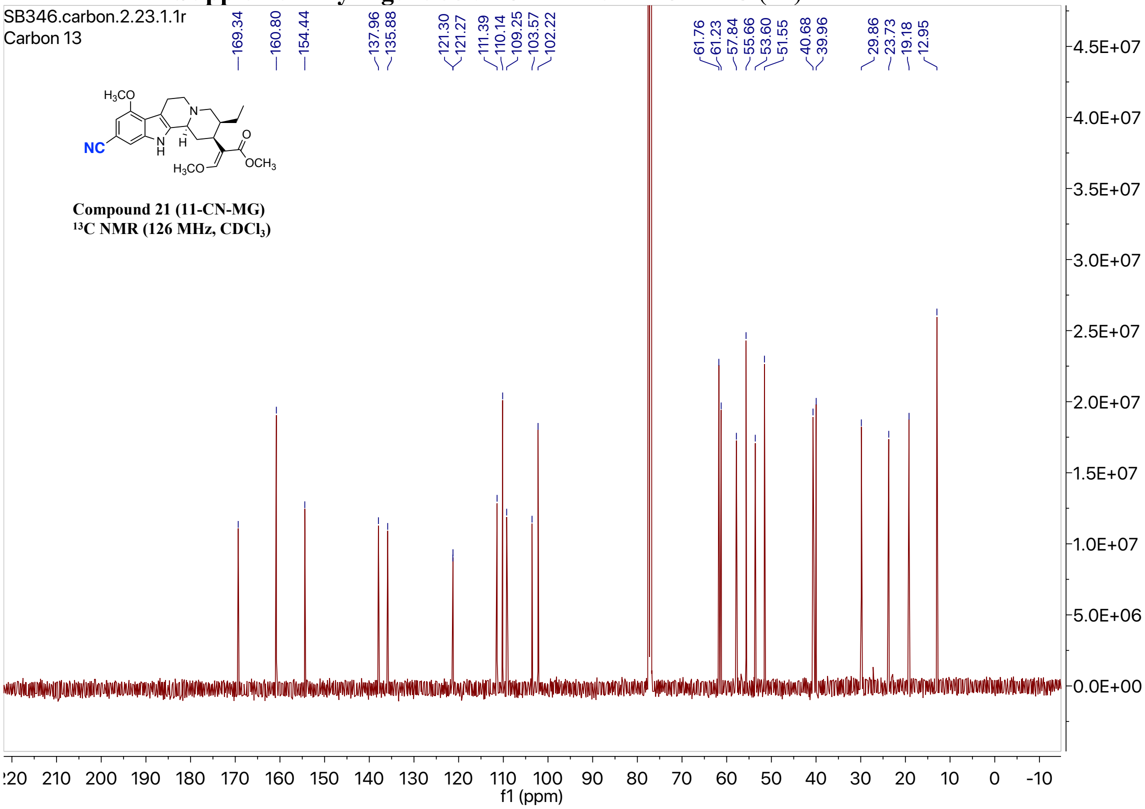

Supplementary Figure 58 : ¹H-NMR of 11-F-7OH (**22**)

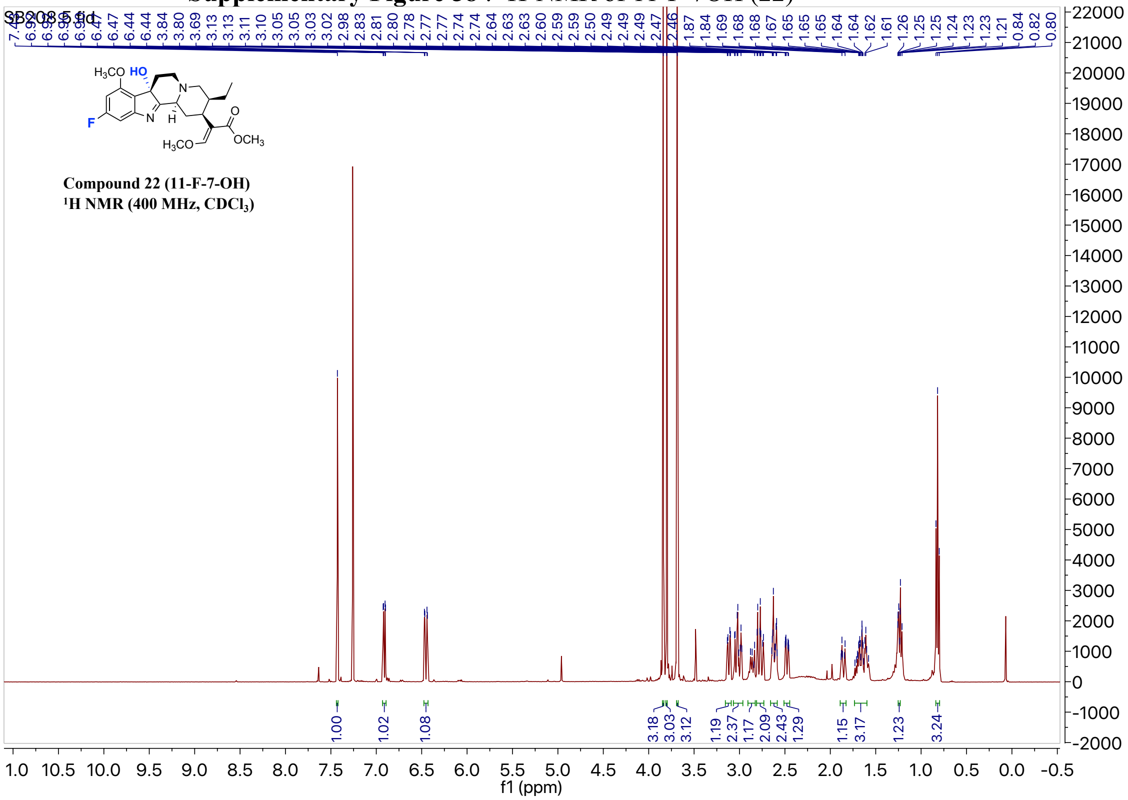

Supplementary Figure 59 : ¹³C-NMR of 11-F-7OH (**22**)

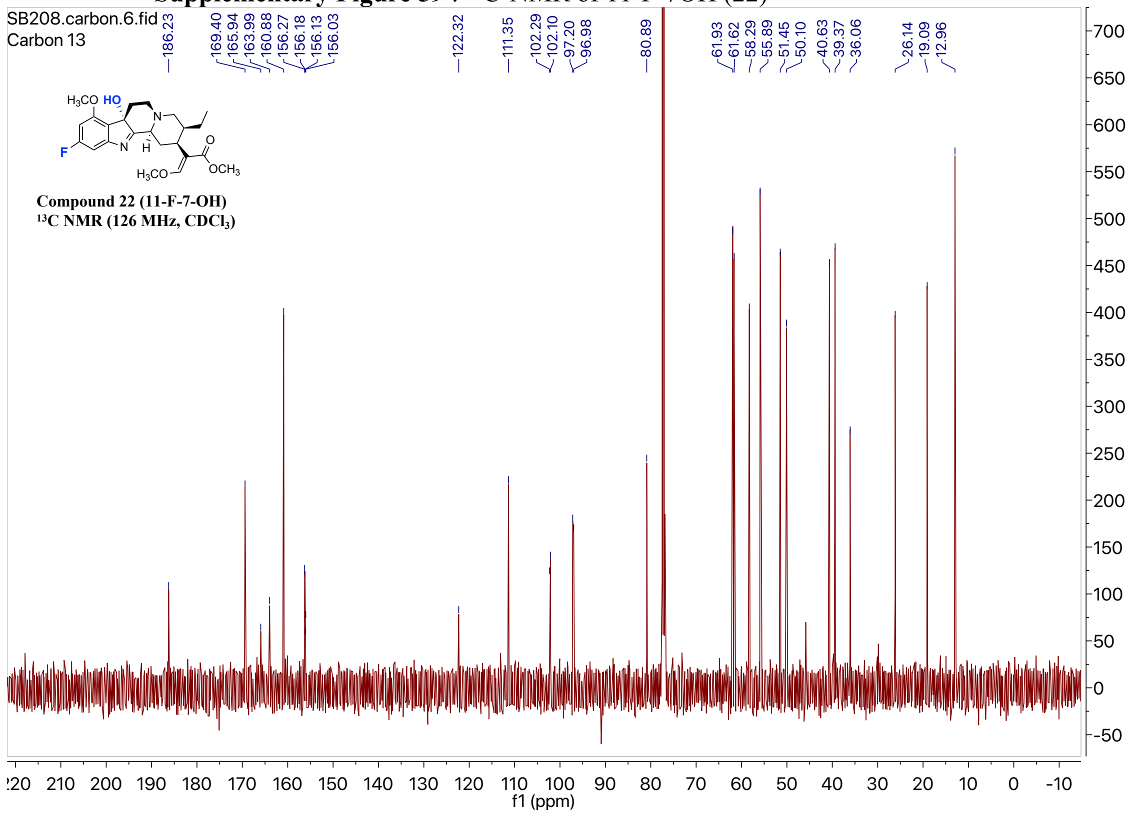

Supplementary Figure 60 : ^{19}F-NMR of 11-F-7OH (**22**)

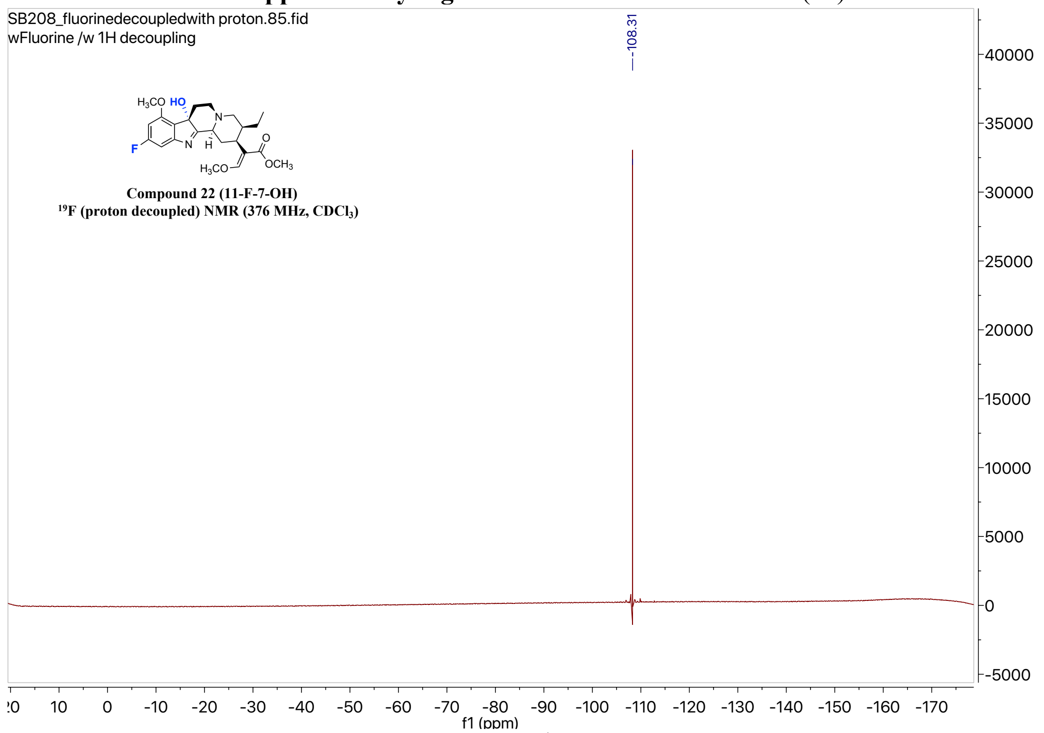

Supplementary Figure 61 : ^{1}H-NMR of 11-Cl-7OH (**23**)

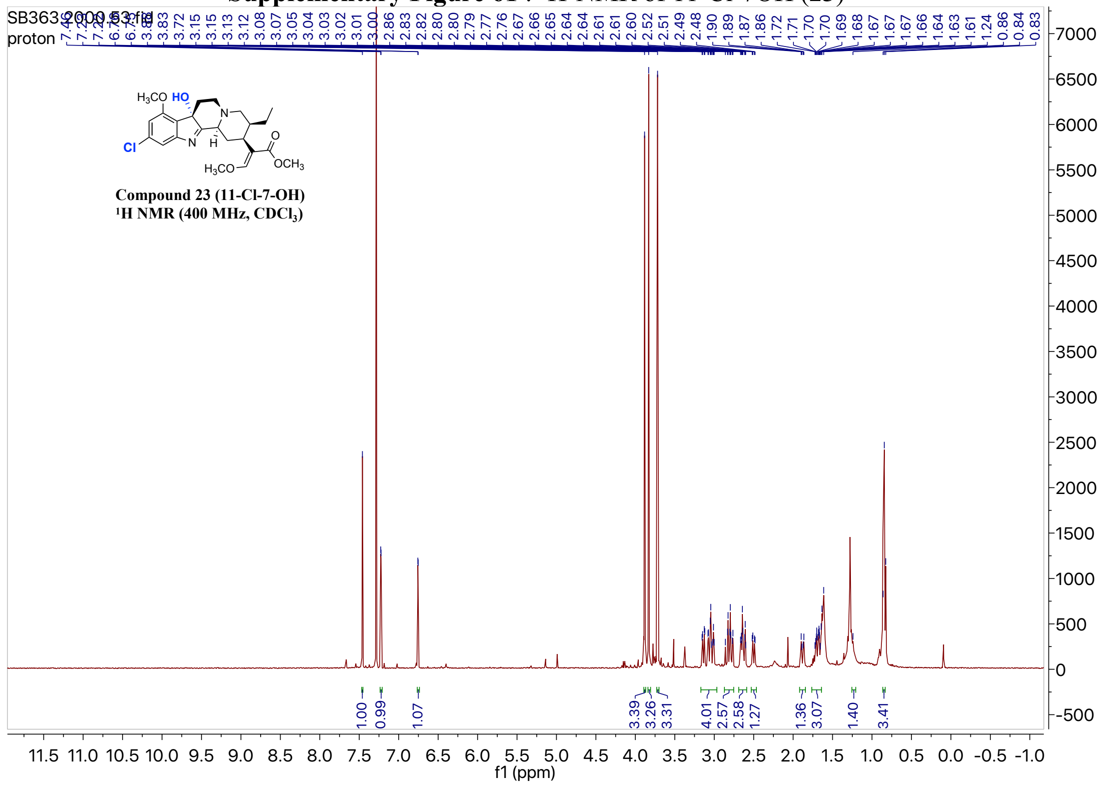

Supplementary Figure 62 : ^{13}Cl-NMR of 11-Cl-7OH (**23**)

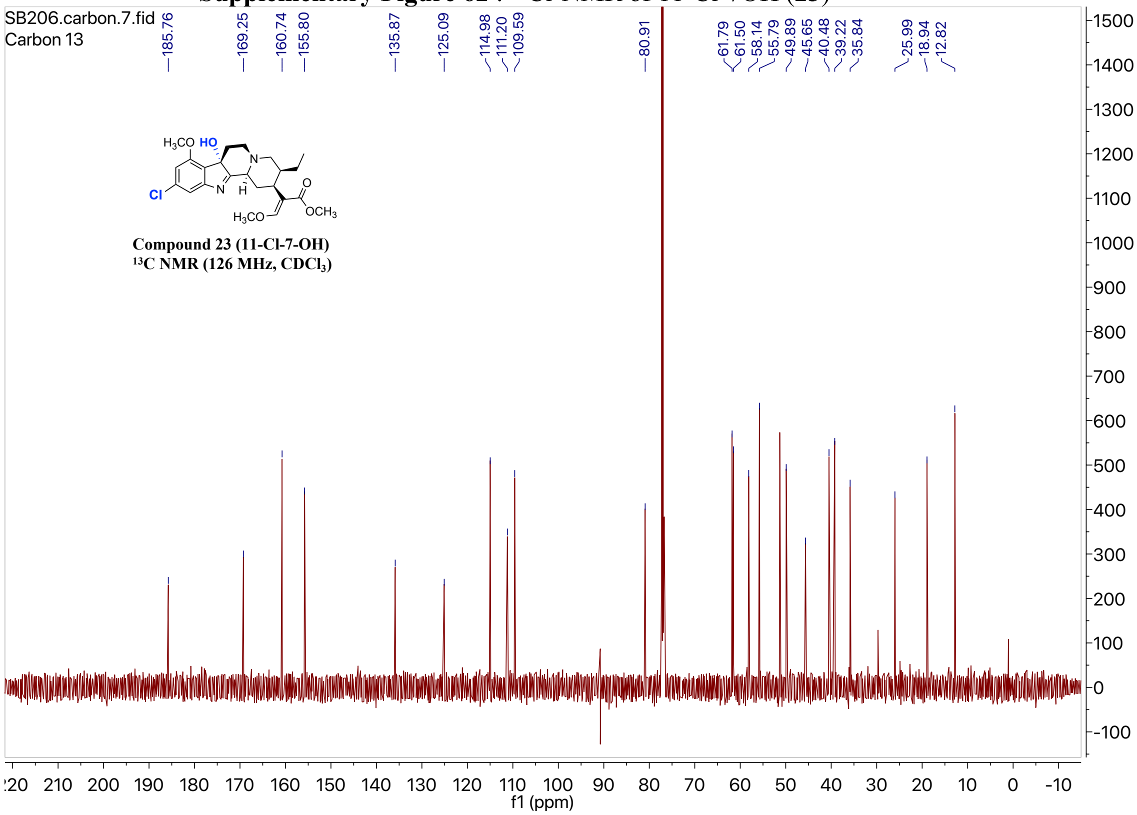

Supplementary Figure 63: ^{1}H-NMR of 11-Br-7OH (**24**)

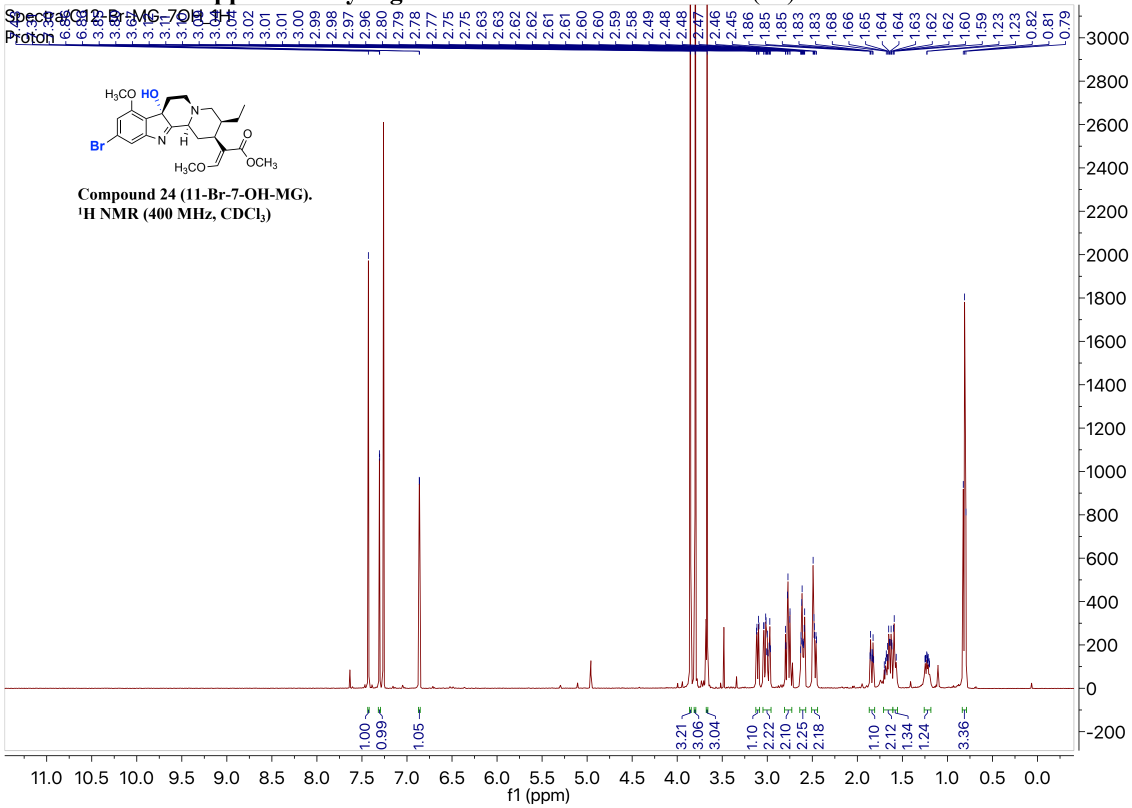

Supplementary Figure 64 : ^{13}C-NMR of 11-Cl-7OH (**23**)

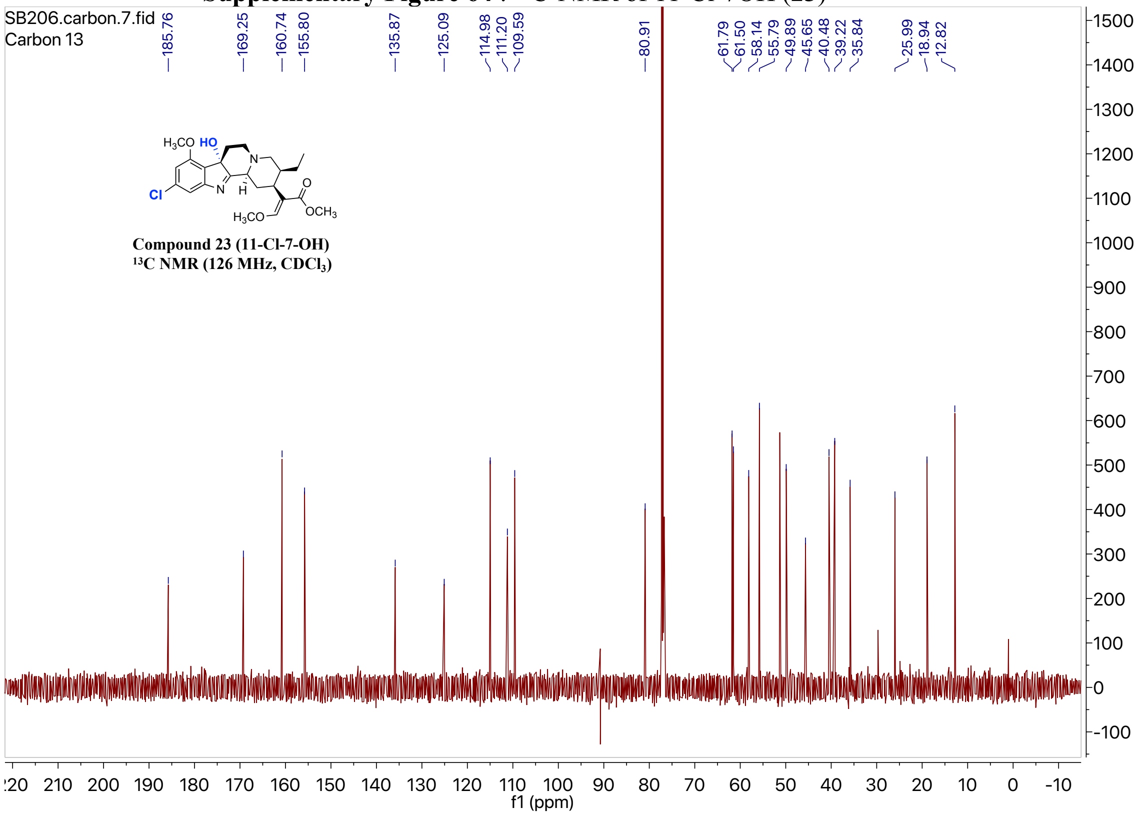

Supplementary Figure 65 : ^{13}C-NMR of 11-Br-7OH (**24**)

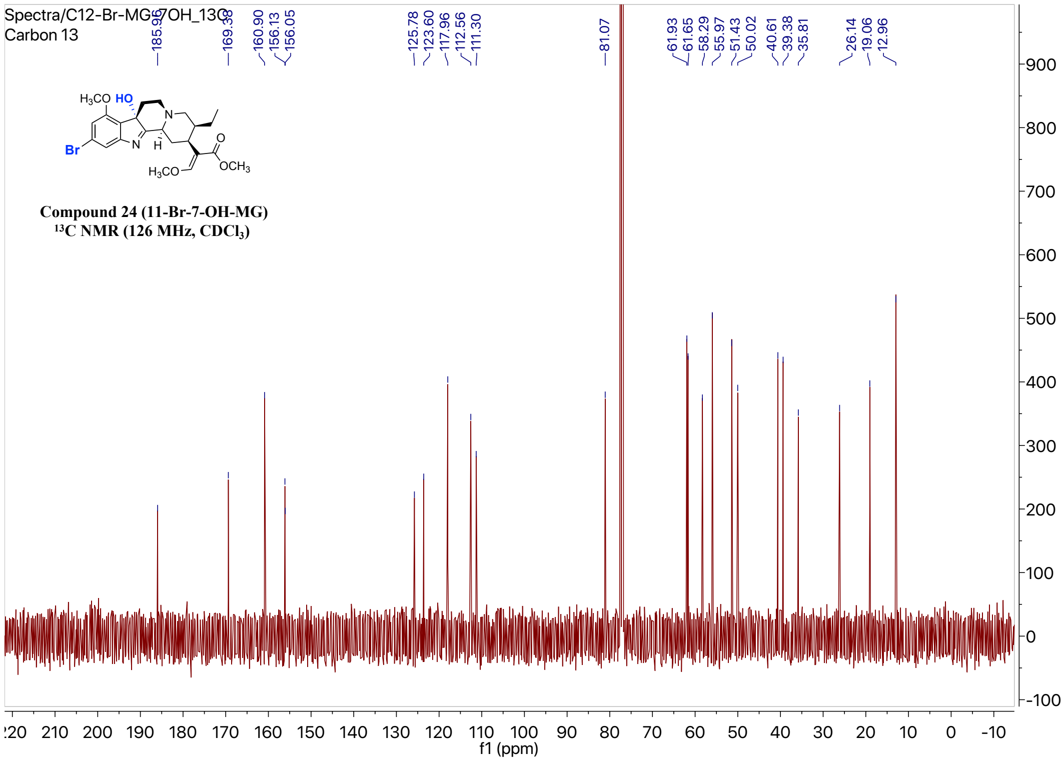

Supplementary Figure 66 : ^{1}H-NMR of 12-Br-7OH (**3**)

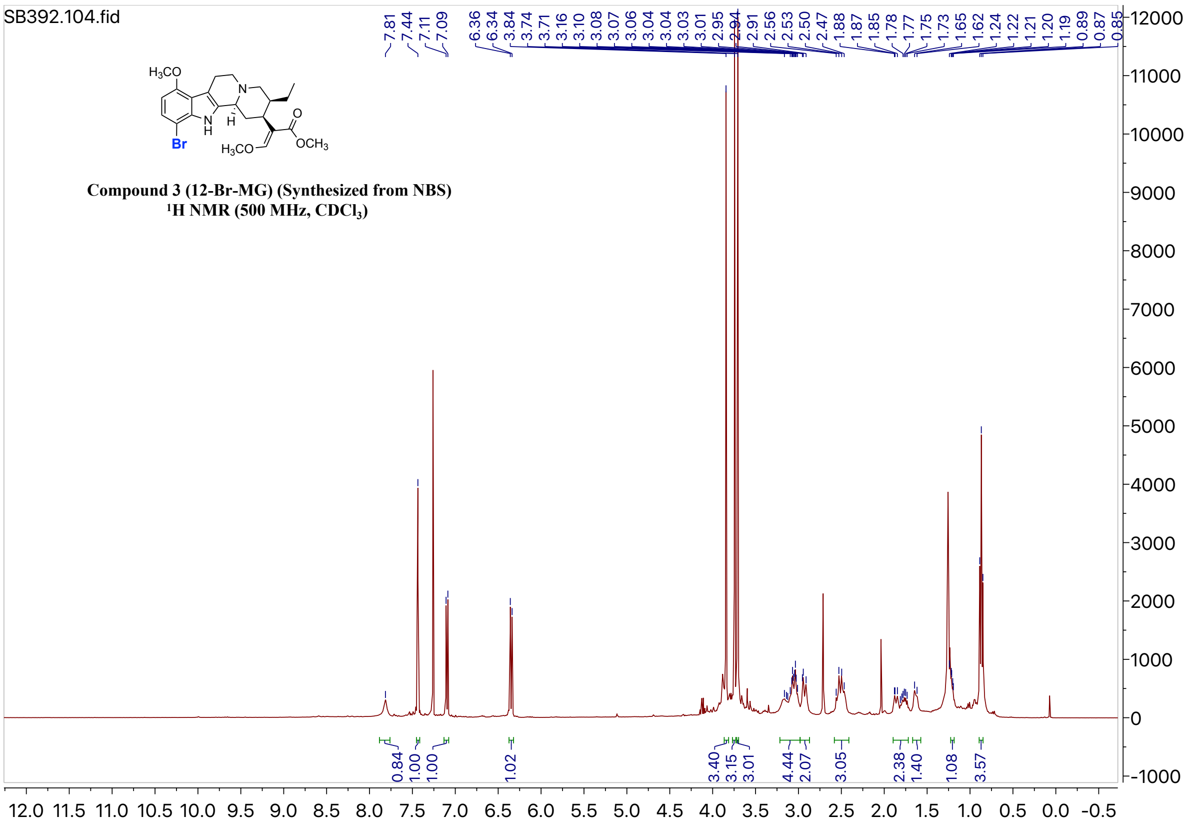

Supplementary Figure 67 : ^{13}C-NMR of 12-Br-7OH (**3**)

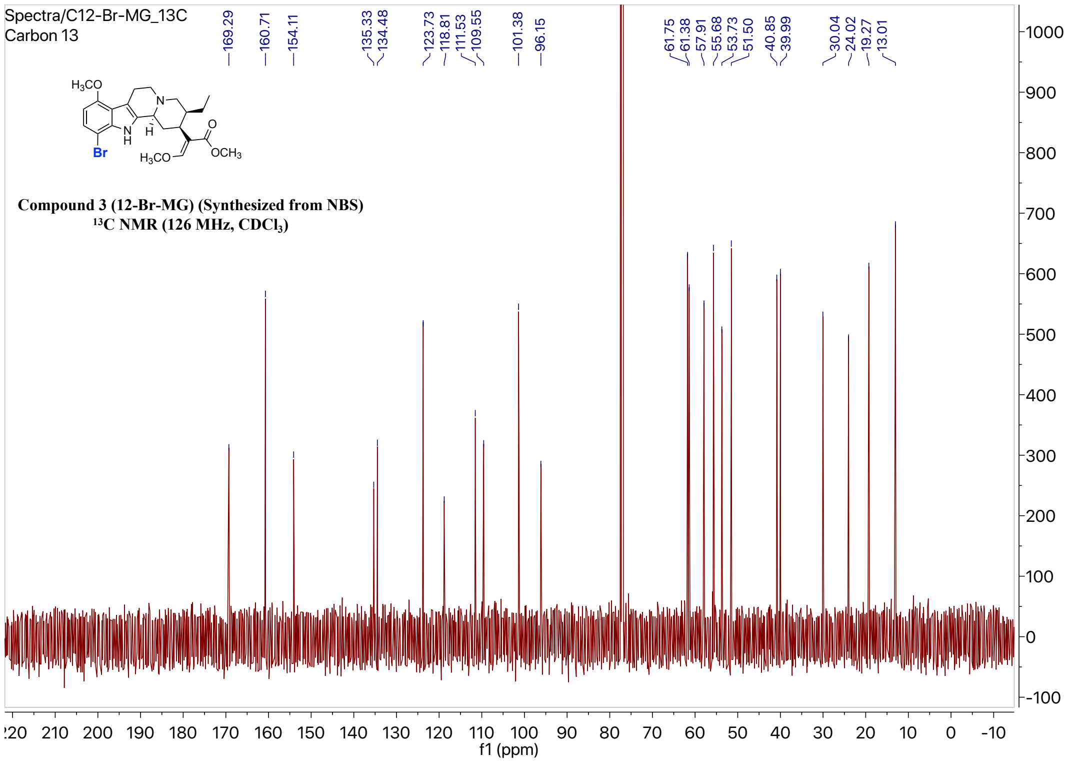

Supplement: Supplementary file 1 — Supplementary Information [file 41467_2021_23736_MOESM1_ESM.pdf]
