# Supplementary material for: Site selective C–H functionalization of Mitragyna alkaloids reveals a molecular switch for tuning opioid receptor signaling efficacy
Source: Nat Commun. 2021 Jun 22;12:3858. doi: 10.1038/s41467-021-23736-2 (PMC8219695; doi:10.1038/s41467-021-23736-2)
Supplement: Supplementary file 2 — Reporting Summary [file 41467_2021_23736_MOESM2_ESM.pdf]

nature research

Corresponding author(s): Prof. Dalibor Sames

Last updated by author(s): Apr 30, 2021

Reporting Summary

Nature Research wishes to improve the reproducibility of the work that we publish. This form provides structure for consistency and transparency in reporting. For further information on Nature Research policies, see our Editorial Policies and the Editorial Policy Checklist.

Statistics

For all statistical analyses, confirm that the following items are present in the figure legend, table legend, main text, or Methods section.

n/a	Confirmed	
☐	☒	The exact sample size (*n*) for each experimental group/condition, given as a discrete number and unit of measurement
☐	☒	A statement on whether measurements were taken from distinct samples or whether the same sample was measured repeatedly
☐	☒	The statistical test(s) used AND whether they are one- or two-sided<br>*Only common tests should be described solely by name; describe more complex techniques in the Methods section.*
☒	☐	A description of all covariates tested
☒	☐	A description of any assumptions or corrections, such as tests of normality and adjustment for multiple comparisons
☐	☒	A full description of the statistical parameters including central tendency (e.g. means) or other basic estimates (e.g. regression coefficient) AND variation (e.g. standard deviation) or associated estimates of uncertainty (e.g. confidence intervals)
☐	☒	For null hypothesis testing, the test statistic (e.g. *F*, *t*, *r*) with confidence intervals, effect sizes, degrees of freedom and *P* value noted<br>*Give P values as exact values whenever suitable.*
☒	☐	For Bayesian analysis, information on the choice of priors and Markov chain Monte Carlo settings
☒	☐	For hierarchical and complex designs, identification of the appropriate level for tests and full reporting of outcomes
☒	☐	Estimates of effect sizes (e.g. Cohen's *d*, Pearson's *r*), indicating how they were calculated

Our web collection on statistics for biologists contains articles on many of the points above.

Software and code

Policy information about availability of computer code

Data collection	Nuclear magnetic resonance spectra were recorded on 400 or 500 MHz instruments as indicated in the Supplementary Information and collected via the Bruker Topspin software (Bruker Topspin 3.5 pl 6).
Data analysis	GraphPad Prism, ver. 5 was used for all statistical analysis for the in vitro assay experiments and GraphPad Prism, ver. 8 was used for all statistical analysis for the tail flick mice assay experiments . Graphs were fit using a 3-parameter logistics equation, (nonlinear regression to fit log(dose) vs. response). Nuclear magnetic resonance spectra were analyzed using Mestronova 14.2.0 software.

For manuscripts utilizing custom algorithms or software that are central to the research but not yet described in published literature, software must be made available to editors and reviewers. We strongly encourage code deposition in a community repository (e.g. GitHub). See the Nature Research guidelines for submitting code & software for further information.

Data

Policy information about availability of data

All manuscripts must include a data availability statement. This statement should provide the following information, where applicable:

- Accession codes, unique identifiers, or web links for publicly available datasets
- A list of figures that have associated raw data
- A description of any restrictions on data availability

The authors declare that all the data supporting the findings of this study are available within the article and Supplementary Information files which contains synthetic procedures and NMR spectra for the featured compounds, additional functional data at rodent and human receptors, biological protocols for receptor binding, activity and antinociception assays. The X-ray crystallographic coordinates for structure 4 reported in this article has been deposited at the

Cambridge Crystallographic Data Center (CCDC) under deposition numbers 1905559. This data can be obtained free of charge from The Cambridge Crystallographic Data Centre via www.ccdc.cam.ac.uk/data_request/cif.A preprint version of this work has been deposited in the public depository ChemRxiv platform https://doi.org/10.26434/chemrxiv.12799787. Source data is provided along with the manuscript.

Field-specific reporting

Please select the one below that is the best fit for your research. If you are not sure, read the appropriate sections before making your selection.

[x] Life sciences [ ] Behavioural & social sciences [ ] Ecological, evolutionary & environmental sciences

For a reference copy of the document with all sections, see nature.com/documents/nr-reporting-summary-flat.pdf

Life sciences study design

All studies must disclose on these points even when the disclosure is negative.

Sample size	Number of technical replicates and biological replicates are reported in the figure and table legends. Sample size was determined based on variability of the response deviating from the mean as indicated by the standard error of the mean (SEM), which is also represented in the figures. Aiming for 95% power with an alpha significance of p=0.05, and an ANOVA analysis with repeated measures within-between interaction, G*Power 3.19.2 was used to calculate a minimum number of mice per testing condition. Analysis suggests a minimum n of 8 mice per condition will be needed for most assays.
Data exclusions	No data were excluded for this study.
Replication	Data were replicated using technical and independent replicates. See figure and table legends for specific details.
Randomization	For the behavioral studies since they were done on mice we did not require to do any randomization as they were not relevant to our study.
Blinding	The screening of analogs (-Cl/F) was done unblinded and then to confirm efficacy and receptor selectivity we ran blinded experiments in two independent labs. The analgesia experiments done on male C57BL/6 mice (22–30 g) mice were performed by blinding the experimenter to the identity of 7OH versus 11-F-7OH. The analgesia experiments done on the wild-type and MOR KO mice were also done by blinding the experimenter to the identity of 11-F-7OH.

Reporting for specific materials, systems and methods

We require information from authors about some types of materials, experimental systems and methods used in many studies. Here, indicate whether each material, system or method listed is relevant to your study. If you are not sure if a list item applies to your research, read the appropriate section before selecting a response.

Materials & experimental systems

n/a	Involved in the study	
[x]	[ ]	Antibodies
[ ]	[x]	Eukaryotic cell lines
[x]	[ ]	Palaeontology and archaeology
[ ]	[x]	Animals and other organisms
[x]	[ ]	Human research participants
[x]	[ ]	Clinical data
[x]	[ ]	Dual use research of concern

Methods

n/a	Involved in the study	
[x]	[ ]	ChIP-seq
[x]	[ ]	Flow cytometry
[x]	[ ]	MRI-based neuroimaging

Eukaryotic cell lines

Policy information about cell lines

Cell line source(s)	HEK293T (CRL-3216) was purchased on 06/08/2017 from American Type Culture Collection (Rockville, MD, USA).<br>CHO-K1 cells were obtained from the American Type Culture Collection American Type Culture Collection (Rockville, MD, USA).
Authentication	Cells have not been authenticated after purchase.
Mycoplasma contamination	Cells have not been tested for mycoplasma
Commonly misidentified lines (See ICLAC register)	No commonly misidentified cell lines were used in the study

Animals and other organisms

Policy information about studies involving animals; ARRIVE guidelines recommended for reporting animal research

Laboratory animals	For analgesic dose-response experiments, male CD1 mice (20–32 g),6-8 weeks were obtained from Charles River Laboratories and male C57BL/6 mice (22–30 g), 8–15 weeks were obtained from Jackson Lab (Bar Harbor, ME) and housed 5 mice per cage in a vivarium following an Institutional Animal Care and Use Committees-approved protocol. For male C57BL/6 mice temperature was kept constant at 22 ± 2°C, and relative humidity was maintained at 50 ± 5%. For male CD1 mice (20–32 g) mice the temperature was in the range of 20-26°C and relative humidity maintained within the range of 30-70%. Mice were given access to food and tap water ad libitum. All mice used throughout the manuscript were opioid naïve. All mice were maintained on a 12 h light/dark cycle with Purina rodent chow and water available ad libitum and housed in groups of five until testing. For analgesic testing in knockout animals, wild-type, male C57BL/6 mice (22–33 g), 10-12 weeks were purchased from the Jackson Lab (Bar Harbor, ME). These mice were kept at a constant temperature of 22 ± 2°C, and relative humidity was maintained at 40-50%. Exon-1/Exon-11 MOR-1 KO mice on a C57 background were bred in the Pintar laboratory at Rutgers University. All mice were maintained on a 12-hour light/dark cycle with food and water available ad libitum, and housed in groups of five until testing. All testing was done in the light cycle.
Wild animals	Study did not involve wild animals
Field-collected samples	Study did not involve samples collected from fields
Ethics oversight	All animal studies were preapproved by the Institutional Animal Care and Use Committees of Washington University School of Medicine and Columbia University, in accordance with the 2002 National Institutes of Health Guide for the Care and Use of Laboratory Animals.

Note that full information on the approval of the study protocol must also be provided in the manuscript.
